# AGILE platform: a deep learning powered approach to accelerate LNP development for mRNA delivery

**DOI:** 10.1038/s41467-024-50619-z

**Published:** 2024-07-26

**Authors:** Yue Xu, Shihao Ma, Haotian Cui, Jingan Chen, Shufen Xu, Fanglin Gong, Alex Golubovic, Muye Zhou, Kevin Chang Wang, Andrew Varley, Rick Xing Ze Lu, Bo Wang, Bowen Li

**Affiliations:** 1 Leslie Dan Faculty of Pharmacy, University of Toronto, Toronto, ON Canada; 2 Department of Computer Science, University of Toronto, Toronto, ON Canada; 3 Vector Institute for Artificial Intelligence, Toronto, ON Canada; 4 Peter Munk Cardiac Centre, University Health Network, Toronto, ON Canada; 5 Institute of Biomedical Engineering, University of Toronto, Toronto, ON Canada; 6 Princess Margaret Cancer Center, University Health Network, Toronto, ON Canada; 7 Department of Chemistry, University of Toronto, Toronto, ON Canada; 8 Department of Laboratory Medicine and Pathobiology, University of Toronto, Toronto, ON Canada

**Keywords:** Drug delivery, Nanoparticles, Mass spectrometry

## Abstract

Ionizable lipid nanoparticles (LNPs) are seeing widespread use in mRNA delivery, notably in SARS-CoV-2 mRNA vaccines. However, the expansion of mRNA therapies beyond COVID-19 is impeded by the absence of LNPs tailored for diverse cell types. In this study, we present the AI-Guided Ionizable Lipid Engineering (AGILE) platform, a synergistic combination of deep learning and combinatorial chemistry. AGILE streamlines ionizable lipid development with efficient library design, in silico lipid screening via deep neural networks, and adaptability to diverse cell lines. Using AGILE, we rapidly design, synthesize, and evaluate ionizable lipids for mRNA delivery, selecting from a vast library. Intriguingly, AGILE reveals cell-specific preferences for ionizable lipids, indicating tailoring for optimal delivery to varying cell types. These highlight AGILE’s potential in expediting the development of customized LNPs, addressing the complex needs of mRNA delivery in clinical practice, thereby broadening the scope and efficacy of mRNA therapies.

## Abstract (web summary)

In this work, the authors develop a platform called AGILE or AI Guided Ionizable Lipid Engineering, which streamlines ionizable lipid development. Using AGILE, they rapidly design, synthesize, and evaluate ionizable lipids for mRNA delivery.

## Introduction

Messenger RNA (mRNA) has emerged as a versatile tool with wide-ranging biomedical applications, ranging from vaccines and protein replacement therapy to cell engineering and gene editing^1,2^. This versatility has fueled widespread interest in exploiting mRNA to tackle an array of diseases^3,4^. However, the inherently unstable nature of mRNA and its susceptibility to nuclease degradation necessitates an effective delivery system, a role typically fulfilled by ionizable lipid nanoparticles (LNPs)^5^. Both Comirnaty and Spikevax, two SARS-CoV-2 vaccines approved amidst the COVID-19 pandemic, are grounded on LNP-based mRNA delivery^6,7^. Moreover, LNP technology helped the first siRNA drug (Onpattro) obtain U.S. FDA approval in 2018^8–10^. The classical LNP formulation comprises four compositions: ionizable lipids, cholesterol, helper lipids, and PEGylated lipids. Notably, each of the three FDA-approved RNA LNPs has a distinct ionizable lipid design, highlighting the pivotal role of ionizable lipids in LNP technology. Their primary functions include packaging mRNA into LNPs and facilitating its entry into the cytoplasm of target cells for ribosomal binding and subsequent protein expression^11–14^. An ionizable lipid generally consists of an ionizable amine head group and two lipid tails. This structure enables protonation at acidic pH, thereby adopting a cationic character during the LNP formulation process, facilitating the encapsulation of anionic RNA molecules. At physiological pH, the ionizable lipid remains neutrally charged, thereby circumventing potential toxicity associated with non-ionizable cationic lipids. Once the LNP encapsulating mRNA is endocytosed, ionizable lipids undergo protonation again in the acidic endosomal environment, disrupting the inner phospholipid membrane of endosomes and promoting the release of mRNA into the cytoplasm of target cells. As the COVID-19 pandemic recedes, the spectrum of mRNA applications continues to broaden beyond vaccination, thus emphasizing the necessity for a diverse array of ionizable lipids proficient in mRNA delivery to a variety of target cells and tissues.

Although previous research has provided some insight into the rational design of ionizable lipids to improve the mRNA delivery performance of LNPs, the approach often covers limited structural space, potentially overlooking some promising lipid designs. Combinatorial chemistry, employing multi-component reactions, has recently been used to enable high-throughput synthesis (HTS) of extensive and chemically diverse lipid libraries. For example, an Ugi-based three-component reaction (3-CR) was used to rapidly synthesize a combinatorial lipid library for high-throughput screening, leading to the identification of a STING-activating ionizable lipid conducive to mRNA vaccine delivery^15^. More recently, another 3-CR system based on the Michael addition was used to generate a library of over 700 ionizable lipids, resulting in the discovery of a potent lipid uniquely suited for efficient mRNA delivery to the lung epithelium^16^. While the 3-CR combinatorial chemistry has been showcased to facilitate the synthesis of ionizable lipids, constructing and testing a more extensive lipid library, comprising up to hundreds of thousands of compounds, for mRNA transfection in different cell targets remains a formidable, time-consuming, and costly task^17^. This challenge consequently restricts efforts to design and test more diverse and innovative structures. The strategies are essential to hasten the discovery and optimization of ionizable lipids for achieving desirable mRNA transfection in specific target cells. Deep learning, a subset of artificial intelligence (AI), poses a promising resolution to the challenge of exploring molecular search spaces^18–21^. With ample high-quality training data, the technique can effectively extract insights from observed molecules, capitalizing on underlying chemical structures and properties, and extrapolating to a broader array of unobserved molecules. Indeed, the rise of deep learning is reshaping chemical compound discovery, transforming this process from a trial-and-error practice to an intelligent, data-driven strategy^22–28^.

In this study, we apply advanced deep-learning methods to expedite the development of ionizable lipids for mRNA delivery, culminating in the AGILE platform. AGILE broadens the molecular range of lipid structures and reduces the development time for ionizable lipids from months or even years to mere days. Leveraging a pre-trained deep-learning neural network, AGILE assimilates structural data from millions of small molecules and analyzes a vast array of unlabeled data from a combinatorial lipid library. This self-supervised learning approach is further refined with wet-lab data, enabling the model to accurately map the relationship between the molecular structures of ionizable lipids and their mRNA transfection capabilities. This dual-phase training process allows AGILE to predict the effectiveness of ionizable lipids in specific cell lines, leading to the quick identification of potential candidates from a large lipid library. In a seminal case, AGILE, augmented with HeLa cell transfection data, identified H9 from 12,000 virtual structures, an ionizable lipid superior in mRNA delivery efficiency to industry-standard benchmarks, both in vitro and post-intramuscular (IM) injection. Furthermore, we demonstrate the versatility of AGILE in rapidly adapting to identify LNPs for different target cells, exemplified by the discovery of R6, a lipid optimized for mRNA delivery to macrophages. Key experimental findings, including the impact of non-biodegradable tail structures in macrophage transfection and the relationship between carbon chain length and mRNA transfection activity, highlight AGILE’s ability to uncover the nuanced interplay between ionizable lipids’ molecular structures and their transfection efficacy. Additionally, AGILE’s adaptability to different cell types indicates its potential to customize LNPs for specific cells, enhancing the effectiveness of mRNA therapies in diverse clinical applications.

## Results

### Overview of the AGILE platform

**Fig. 1:**
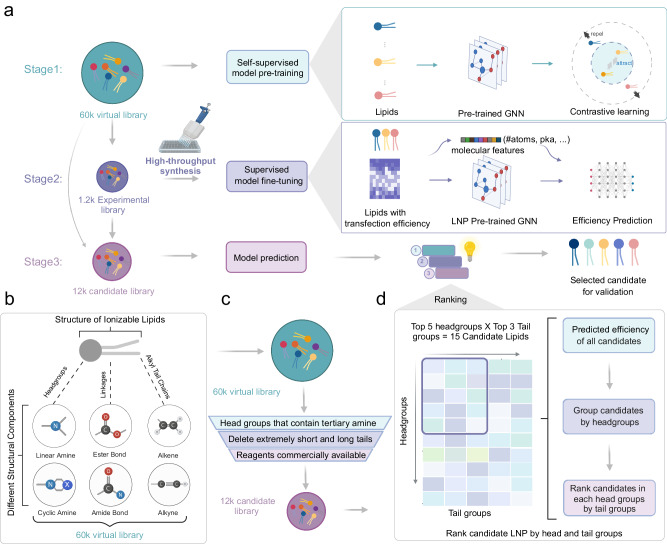
Overview of the platform design pipeline. **a** Illustration of the 3-stage workflow of the platform. Stage 1: construction of a virtual library and self-supervised pre-training of the model. Stage 2: synthesis of an experimental library for the fine-tuning of the model in a supervised manner. Stage 3: deployment of the fine-tuned model for predictive analysis on a candidate library, followed by ranking for final candidate selection (GNN graph neural network). **b** Depiction of virtual library design through the application of 3-CR (three components reaction) Ugi combinatorial chemistry. **c** Schematic representation of the rational selection process for lipid candidates, with three listed filtering criteria. **d** A comprehensive breakdown of the ranking procedure and the selection methodology for final candidates. The figure was created with BioRender.com and released under a Creative Commons Attribution-NonCommercial-NoDerivs 4.0 International license.

AGILE synergistically combines deep-learning methodologies with high-throughput combinatorial lipid synthesis chemistry to enhance the discovery of ionizable lipids essential for LNP-based mRNA delivery. At its core, the AGILE model, comprising deep learning algorithms including a graph encoder and a molecular descriptor encoder, adeptly discerns the unique characteristics and chemical properties of ionizable lipid molecular structures. The implementation of AGILE unfolds in three pivotal stages: (1) developing a virtual library and initiating self-supervised model training; (2) refining the model’s accuracy through supervised fine-tuning with empirical data from an experimental library; and (3) conducting in silico analysis on ionizable lipids in a candidate library, utilizing the refined deep learning algorithms (Fig. 1a and refer to section “Methods” for more details). Following its initial self-supervised pre-training on a virtual lipid library and further refinement with high-throughput wet-lab data, AGILE’s deep-learning neural network adeptly unravels the intricate relationship between ionizable lipids’ molecular structures and their mRNA transfection capabilities. This process is designed to enable AGILE to accurately predict the efficacy of ionizable lipids in LNP formulations for mRNA delivery, thereby streamlining the identification of viable candidates from a vast lipid library.

Specifically, Stage 1 aims to develop a graph encoder proficient in differentiating and depicting distinct lipids through pre-training on a vast collection of unlabeled lipid molecules (section “Methods”). This process begins with the construction of a graph encoder utilizing graph neural networks (GNN), primed with parameters from the MolCLR model^29^, which has undergone pre-training on a repertoire of over ten million small molecules. This “warm-starting” strategy, embedding general knowledge of small molecular structures into our algorithm, fortifies the accuracy of AGILE in subsequent stages (Supplementary Fig. S1). The graph encoder subsequently underwent continuous pre-training on a virtual library of 60,000 chemically diverse lipids through contrastive learning^30^, enabling the differentiation of atoms and bonds in each molecule, and thus capturing the disparities amongst various lipid structures (section “Methods”). The virtual library, featuring lipids with varied amine head groups and two distinct alkyl chains (Fig. 1b), is constructed using principles of 3-CR chemistry, making it suitable for high-throughput combinatorial synthesis^31^. The initial pre-training in Stage 1 endows the graph encoder with an extensive understanding of lipid structures, thereby fortifying the subsequent stages of the process (Supplementary Fig. S1).

In Stage 2, the AGILE model is further trained with wet-lab mRNA transfection data from a range of ionizable lipids. We synthesized 1200 lipids using 3-CR chemistry and assessed their mRNA transfection potency (mTP), defined here as a measure of mRNA’s cell transfection efficacy. The data collected was then utilized to refine the AGILE model through supervised learning (section “Methods”). To improve its generalizability and accuracy, a molecular descriptor encoder was integrated, which processes molecular descriptors computed by the Mordred toolbox (section “Methods”)^32^. The encoder’s output updates the representation of lipid structures in the pre-trained graph encoder. Consequently, the AGILE model is trained to minimize discrepancies between its predictions and actual wet-lab results during the fine-tuning phase. For in silico screening in Stage 3, we created a candidate library of 12,000 lipid structures, selectively curated from the Stage 1 virtual library (Fig. 1c) based on three criteria (section “Methods”): (1) exclusion of non-ionizable cationic lipids to mitigate toxicity risks^33^; (2) removal of lipids with either excessively short (<C10) or long (>C18) alkyl chains, informed by empirical findings^15^; and (3) elimination of lipids for which reagents are not available. The AGILE model then predicts the mTP of lipids in this candidate library, employing a head and tail-wise ranking method to enhance the structural diversity among the top candidates (Fig. 1d, section “Methods”). The results from AGILE guide the selective synthesis and formulation of the highest-ranked ionizable lipids into LNPs, which are further tested in the wet lab to validate their efficacy in mRNA delivery to specific target cells.

### Combinatorial synthesis and screening of lipid library for fine-tuning

**Fig. 2:**
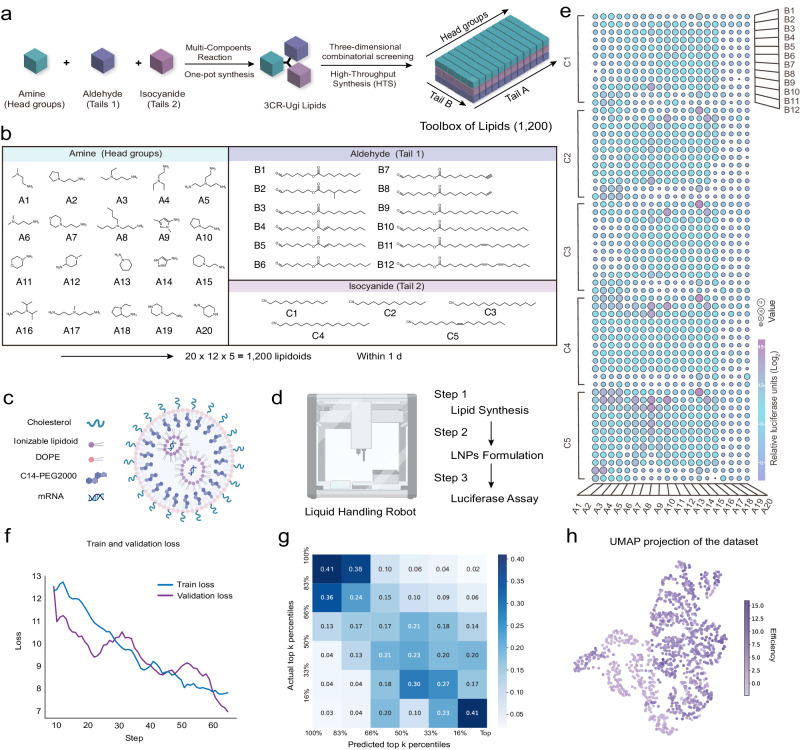
High-throughput lipids synthesis and screening platform. **a** A schematic to illustrate the high-throughput synthesis method for the toolbox of lipids (1200). **b** The combinatorial lipids library consists of three components structure (amine headgroups, aldehyde tails, and isocyanide tails). **c** A schematic diagram shows the lipid nanoparticles (LNPs) components for mRNA encapsulating. **d** Lipid synthesis, LNPs formulation, and luciferase assay are automated by a liquid handling robot. **e** The data used for the fine-tuning are depicted in a balloon plot, which involved 1200 lipids for Fluc mRNA (mFLuc) delivery and measuring the relative luciferase expression in HeLa cells. **f** The loss value on the training set and validation set against fine-tuning steps. **g** The precision matrix computed on the experimental library of 1200 lipids. The mRNA transfection potency (mTP) is divided into six equal percentiles, which are predicted and compared to the actual results. **h** Uniform Manifold Approximation and Projection (UMAP) plot of the experimental library, colored by the mTP. Panels (**c**, **d**) were created with BioRender.com and released under a Creative Commons Attribution-NonCommercial-NoDerivs 4.0 International license.

To efficiently create diverse libraries of ionizable lipids, we utilized an HTS platform, leveraging the one-pot Ugi 3-CR method (Fig. 2a and Supplementary Fig. S2). Automated robotic liquid handling facilitated the synthesis of 1200 ionizable lipids in just a day. Our experimental lipid library comprised 20 unique head groups, 12 alkyl chains with biodegradable ester linkages, and 5 alkyl chains with isocyanide functional groups^34^ (Fig. 2b). This automation also enabled rapid formulation of these ionizable lipids into LNPs using a standardized four-component formulation ratio^35,36^, reducing operator variability (Fig. 2c). The LNPs were loaded with firefly luciferase mRNA (mFluc) to quickly assess mTP in target cells, which herein was measured as the base 2 logarithm of the luminescence intensity ratio between transfected and untreated cells 24 h post-treatment, a timeframe chosen to capture peak bioluminescent signal based on mFluc expression kinetics in cells (Supplementary Fig. S3). The entire processes of lipid synthesis, LNP formulation, and luciferase assay were streamlined and automated using the liquid handling robot (Fig. 2d). To validate the suitability of the automated liquid processor for LNP formulation, we compared it against the conventional manual pipette method^37^. Analysis of the results found no significant differences between the automated liquid processor and manual pipette in terms of LNP properties (Supplementary Fig. S4).

For initial screening, mFluc-loaded LNPs, prepared from 1200 ionizable lipids, were tested in HeLa cells, and the resulting mTP data were utilized to refine AGILE (Fig. 2e, Supplementary Fig. S5). This fine-tuning stage (section “Methods”) helped the model to understand the correlation between molecular properties and mTP. We allocated 80% of this data for training, 10% for hyperparameter optimization, and 10% for internal validation. As the training progressed, we noted a consistent decrease in loss value on both the training and validation data (Fig. 2f), verifying the convergence of the model optimization. Consequently, the model checkpoint exhibiting the minimal validation loss was selected for further application upon the completion of the training. To assess prediction accuracy, we categorized the predicted and actual in vitro mTP values into six equal percentiles, and observed the model’s proficiency in identifying the highest and lowest performing lipids (Fig. 2g). For example, lipids predicted to be in the top 16% had a 0.41 probability of actually being in that tier in vitro. The AGILE model’s effectiveness was further benchmarked against traditional machine learning (ML) algorithms including Ridge regression^38^, Lasso^39^, Gradient Boosting^40^, and Support Vector Machines (SVM)^41^, where it consistently outperformed them (section “Methods”). Additionally, we projected the lipid embeddings onto two-dimensional space using UMAP (Fig. 2h)^42^. The ionizable lipids with similar mTP values were spatially grouped together in the resulting UMAP plot, demonstrating the accurate alignment between the model’s representation and the mTP in the wet lab.

### Identification of ionizable lipid by AGILE for muscle-selective mRNA delivery

**Fig. 3:**
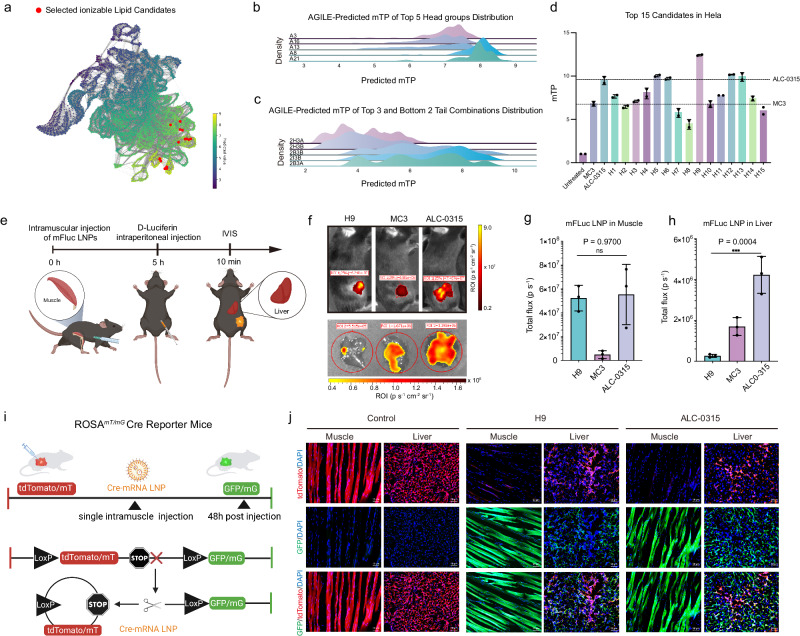
Model prediction and the validation of the gene editing potential with top-performing mRNA LNPs. **a** The Uniform Manifold Approximation and Projection (UMAP) plot of the predicted mRNA transfection potency for lipids. **b** Headgroups distribution and **c** tail combinations distribution in HeLa. **d** Transfection test of 15 lipid candidates selected by AI-Guided Ionizable Lipid Engineering (AGILE) platform in HeLa cells (*n* = 2). **e** Illustration of in vivo transfection study: mRNA transfection potency (mFluc) LNPs were intramuscularly injected into the mice followed by IVIS imaging at 6 h post-injection. **f** IVIS imaging of injection sites and livers collected from mice after intramuscular injection of mFluc LNPs, containing H9, MC3, and ALC-0315 were injected intramuscularly into mice respectively (*n* = 3 biologically independent mice/group, 0.5 mg kg^−1^ mFLuc per mouse). **g** Quantification of luminescent intensity at the intramuscular injection site in mice (*n* = 3 biologically independent mice/group). **h** Quantification of luminescent intensity in mouse livers (*n* = 3 biologically independent mice/group). **i** Illustration of the mTmG (membrane-Tomato/membrane-Green) mouse model: in the absence of Cre recombinase, tdTomato (tandem dimer Tomato) reporter is expressed; the presence of Cre recombinase deletes STOP cassettes and results in the expression of green fluorescent protein (GFP) reporter in mice. **j** Representative confocal microscopy images and quantification of tdTomato and GFP expression in histological muscle and liver sections of mTmG mice post-injection of Cre mRNA loaded LNPs by intramuscular injection. Scale bar: 50 µm. Sections from 3 mice (*n* = 5). Red represents tdTomato, Green represents GFP, and blue represents the nucleus (DAPI). Statistical significance was analyzed by a two-way analysis of variance (ANOVA) with *t*-test. Source data are provided as a Source Data file. Panels (**e**, **i**) were created with BioRender.com and released under a Creative Commons Attribution-NonCommercial-NoDerivs 4.0 International license.

Using the AGILE model, fine-tuned with mTP data from HeLa cells, we predicted potential lipids for IM mRNA delivery from a virtual candidate library. Our predictions, visualized through UMAP (Fig. 3a), demonstrated a clear distinction between high and low predicted values, underscoring the model’s capability to differentiate between more and less effective ionizable lipids in a broad screening library. Detailed analysis of the stratified distribution plots (Fig. 3b and Supplementary Fig. S5) showed that predicted potencies were distinctly sorted by combinations of head groups and tails. Notably, among the top five head groups, A8 and A21 stood out with higher predicted potencies. Although tail combinations exhibited less distinct stratification in predicted transfection potencies than head groups (Fig. 3c and Supplementary Fig. S6), the top tails remained crucial for candidate selection. The model’s preference for unsaturated alkyl chains (Supplementary Fig. S7) aligns with existing literature^43,44^. Adopting our ranking system, which emphasizes structural diversity in lipids by considering both head and tail combinations (Fig. 1d), we selected the top 15 lipid candidates for further experimental validation (Supplementary Fig. S7). We synthesized and evaluated the top 15 lipid candidates identified by AGILE, confirming their efficacy in mediating mRNA transfection in HeLa cells (Fig. 3d). Notably, the transfection potency of the standout candidate, H9 was markedly superior to other top lipids from the initial 1200-lipid set, demonstrating AGILE’s effectiveness in identifying potent ionizable lipids (Supplementary Fig. S8). To thoroughly assess H9’s effectiveness, we compared it against ALC-0315, the industry-standard ionizable lipid used in Pfizer/BioNTech’s COVID-19 mRNA vaccine, using formulations containing either DOPE or DSPC as helper lipids. In both cases, H9 demonstrated consistently superior performance compared to ALC-0315, underscoring its exceptional efficacy (Supplementary Fig. S9).

In previous studies, HeLa cells have been used as an initial model for high-throughput screening of ionizable lipid libraries in mRNA delivery to muscle tissues^15,45,46^. Here, we sought to validate whether transfection potency in HeLa cells could reliably predict lipid performance post-IM injection, as compared to using muscle cells. To do so, we assessed the correlation between the transfection potency of the top 15 lipids identified by AGILE in HeLa cells and their performance post-IM mRNA delivery. This was compared to the correlation between the transfection potency of these lipids in C2C12 mouse myoblast cells and their subsequent potency post-IM mRNA delivery. The results revealed similar Pearson correlation coefficients (PCCs) for mFluc expression in HeLa cells (0.78) and C2C12 cells (0.756) relative to their IM delivery performance in mice (Supplementary Figs. S10 and S11). This resemblance supports the use of HeLa cells as effective substitutes for muscle cells in the initial high-throughput screening of LNPs for IM mRNA delivery. Hence, before further in vivo assessment of H9-containing LNPs for IM mRNA delivery, we further optimized its formulation using a Design of Experiments (DoE) approach in HeLa cells (Supplementary Table 3 and Fig. S12). This led to a formulation that was both uniform and stable, displaying significantly higher mTP than benchmark LNPs MC3 (D-Lin-MC3-DMA)^2^ (Supplementary Figs. S13 and S14).

Next, we compared the efficacy of post-DoE H9 LNPs to industry-standard benchmark LNPs for mRNA delivery in mice via IM injection (Fig. 3e). Our findings revealed that H9 LNPs delivered mRNA to muscle tissue with an efficiency of 7.8 times greater than MC3, comparable to ALC-0315 (Fig. 3f, g). Intriguingly, H9 LNPs displayed remarkable tissue specificity, achieving substantially lower mRNA expression in major organs such as the liver and spleen compared to both MC3 and ALC-0315 LNPs (Fig. 3f, h). To further validate this finding, we administered H9 and ALC-0315 LNPs loaded with Cre recombinase mRNA to mTmG reporter mouse models^47^. These mice harbor gene mutations in the Gt(ROSA)26Sor locus, and upon Cre mRNA expression, the mT cassette is excised in Cre-expressing tissue, enabling the expression of the downstream membrane-targeted green fluorescent protein (GFP, mG) cassette (Fig. 3i). Consistent with our bioluminescence studies, we observed similar levels of GFP expression at the IM injection sites in mice for both H9 and ALC-0315 LNPs, but significantly lower GFP expression in the liver was observed with H9 LNPs (Fig. 3j and Supplementary Fig. S15). To elucidate H9 LNP’s muscle-specific transfection efficacy, we examined its biodistribution post-IM injection using Cyanine5 (Cy5) labeled mFLuc. When administered intramuscularly to C57BL/6 mice, the distribution of H9 LNPs in muscle tissue was comparable to that of ALC-0315 LNPs (Supplementary Fig. S16). However, H9 LNPs showed notably less accumulation in the liver, aligning with findings from the bioluminescence study. This suggests that H9 LNP’s muscle-specific transfection is possibly due to its reduced distribution to non-target tissues.

Further, to assess H9 LNPs’ effectiveness and safety for vaccination, we used LNPs encapsulating mRNA encoding full-length ovalbumin (mOVA) in a prime-boost vaccination schedule in BALB/c mice. The anti-OVA IgG titers induced by H9 LNPs were comparable to those elicited by ALC-0315 LNPs (Supplementary Fig. S17a, b). Notably, H9 LNP administration resulted in lower ALT/AST serum levels compared to ALC-0315 LNPs, indicating a potentially lower risk of hepatotoxicity (Supplementary Fig. S17c, d). This finding is particularly relevant in light of clinical reports associating ALC-0315-based COVID-19 mRNA vaccines (BNT162b2) with cases of autoimmune hepatitis post-vaccination^48–50^. Therefore, H9 LNPs, identified via the AGILE platform, may offer a safer alternative with reduced off-target effects and lower potential for hepatitis-related side effects. Together, these results, which include targeted transfection and favorable biodistribution in muscle tissues, position H9 LNPs as a promising and safer candidate for vaccinations, underlining the potential benefits of AGILE-driven LNP development in enhancing vaccine safety and efficacy.

In addition, we also synthesized and tested 15 lower-ranked candidates (ranked 31–45) (Supplementary Figs. S18, S19 and Supplementary Tables 1 and 2). Unlike the top 15 candidates, none in this group exceeded the mRNA delivery efficiency of the benchmark lipid ALC-0315. Furthermore, testing of the lowest-ranked lipid candidates identified by AGILE (H9138, H9239, H9252) showed only minimal mFluc expression (Supplementary Fig. S20). Overall, these mid- and bottom-tier candidates generally exhibited lower mRNA delivery efficiency compared to the top 15. This correlation between AGILE’s predictive results and the experimental performance underscores its precision in identifying effective ionizable lipids.

### AGILE-assisted discovery of ionizable lipids for mRNA delivery to macrophages

**Fig. 4:**
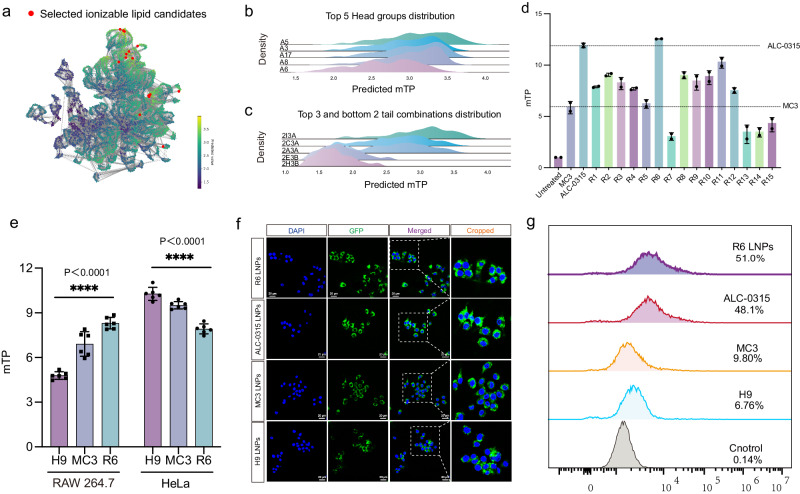
Accelerating screening of lipids for eGFP-mRNA delivery in macrophage through the platform. **a** The UMAP plot of the predicted mRNA transfection potency (mTP) for lipids. **b** Top five headgroups distribution and **c** top three and bottom two tail combinations distribution in RAW 264.7. **d** Validation of 15 lipid candidates in RAW 264.7 cells (*n* = 2). **e** Comparison of the mTP of H9, MC3, and R6 LNPs in RAW 264.7 cells and HeLa cells (*n* = 6). **f** Confocal images of RAW 264.7 cells transfected by GFP-mRNA LNPs (*n* = 3). Scale bar: 20 µm. Green represents GFP and blue represents the nucleus (DAPI). **g** Percentage of GFP positive cells on RAW 264.7 after treatment with MC3, ALC-0315, H9, and R6 LNPs. Statistical significance was analyzed by a two-way analysis of variance (ANOVA) with *t*-test. **** Represented *p* < 0.0001. Source data are provided as a Source Data file.

To evaluate AGILE’s adaptability to different cells, we gathered mTP data for the same pool of 1200 LNPs in the macrophage cell line RAW 264.7 (Supplementary Figs. S21, S22). After fine-tuning AGILE with this macrophage dataset, we used UMAP to predict and visualize mTP for lipid candidates in RAW 264.7 cells (Fig. 4a). The UMAP analysis for RAW 264.7 cells revealed a broader dispersion of top-tier ionizable lipids compared to HeLa cells, suggesting a potentially higher complexity in mTP prediction for macrophages. On the other hand, the mTP predictions for RAW 264.7 cells showed clear stratification based on head groups and tail combinations (Fig. 4b, c and Supplementary Fig. S23), similar to the results in HeLa cells. Subsequently, we synthesized and evaluated the top 15 candidates in RAW 264.7 cells, with 11 showing higher mTP than benchmark MC3 (Fig. 4d and Supplementary Fig. S24). Further, R6, the most promising lipid for macrophages among the top candidates, underwent formulation optimization using DoE methodology^51^ (Supplementary Table 4 and Supplementary Fig. S25). Guided by prior research, this optimization included substituting traditional zwitterionic helper lipids with DOTAP to potentially enhance mRNA transfection^16^. Our results (Supplementary Fig. S26a) demonstrated the mTP of R6 LNPs in macrophages could be further improved when DOTAP was included in the formulation.

To evaluate the cell-type specificity of LNPs developed using AGILE, we conducted a comparative analysis between R6 and H9 LNPs, which have identical lipid compositions but differ in their core ionizable lipids, with the conventional MC3-LNP formulation serving as a benchmark. The results R6 LNPs were more effective in mRNA delivery to RAW 264.7 cells, whereas H9 LNPs were superior in HeLa cells (Fig. 4e and Supplementary Fig. S26b, c). A cytotoxicity study confirmed that these differences in mRNA delivery efficiency were not due to cytotoxicity (Supplementary Fig. S27a). Further, when delivering GFP-mRNA to RAW 264.7 cells, R6 LNPs demonstrated a significant fivefold increase in transfection efficiency compared to H9 and MC3 LNPs (Fig. 4f, g). This highlights AGILE’s capability in identifying ionizable lipids for macrophage transfection, emphasizing its utility in creating targeted non-viral mRNA delivery vectors for immune cells.

Additionally, in line with the observed correlation between mTP in HeLa cells and IM mRNA delivery efficacy, R6 LNPs exhibited lower transfection performance in muscle tissues compared to H9 LNPs following IM injection (Supplementary Fig. S27b), with notable retention at the muscle site and limited distribution to other organs (Supplementary Fig. S28a). This suggests that while R6 LNPs are highly effective for macrophage transfection, their efficacy in muscle-specific transfection is constrained. To explore R6 LNP’s potential in mRNA vaccine applications, we immunized mice intramuscularly with mOVA-loaded R6 LNPs alongside ALC-0315 LNPs, following a prime-boost schedule. Despite showing a safety profile similar to ALC-0315 LNPs, R6 LNPs induced lower anti-OVA IgG levels (Supplementary Fig. S28b–d), highlighting the necessity for LNPs to transfect both immune cells and local muscle tissues effectively to achieve optimal mRNA vaccine efficacy.

### Interpretation of the AGILE deep learning model

**Fig. 5:**
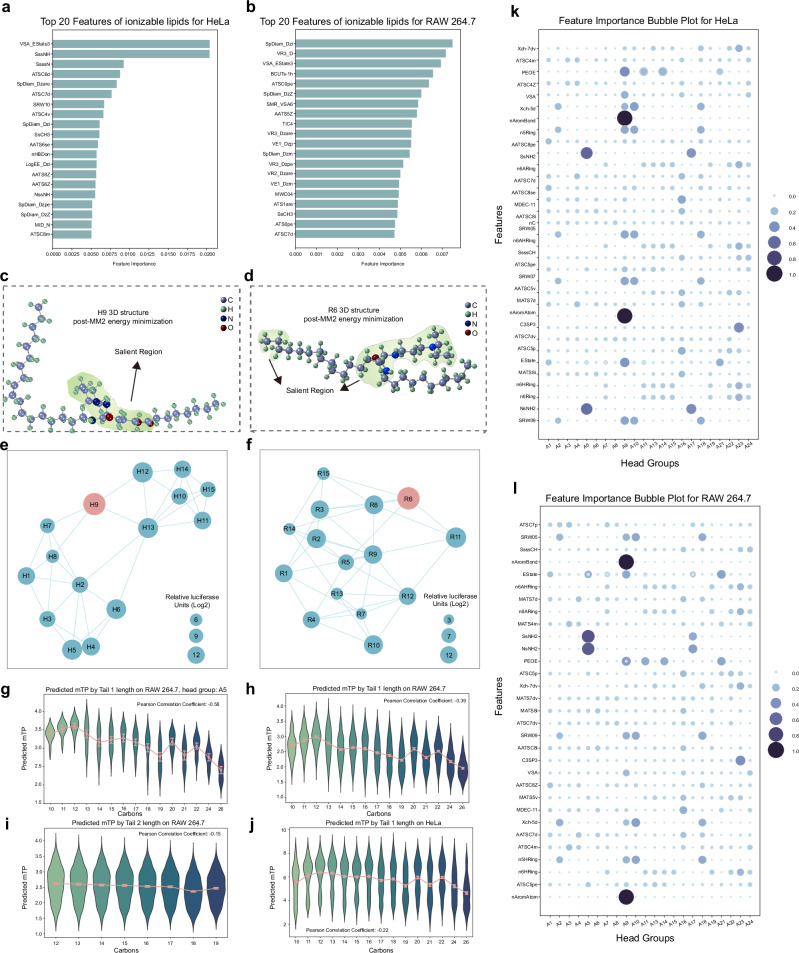
Model feature explanation and finding. **a**, **b** This model has identified the top 20 most important molecular descriptors, which have been fine-tuned for HeLa and RAW 264.7. **c**, **d** Three-dimensional visualizations of the H9 and R6 structures, with the important regions highlighted for easy identification. **e**, **f** The top 15 lipid candidates in HeLa and RAW 264.7 cells have been organized into similarity networks, with each candidate being connected to its four closest neighbors. **g** The violin plot shows how the predicted potencies are distributed among Tail 1 with varied carbons for the RAW 264.7 cells, using lipids with the most effective headgroup A5 (*n* = 9, 18, 27, 45, 54, 53, 35, 39, 45, 36, 27, 27, 40, 21, and 27, respectively). **h** A similar violin plot as in (**g**) but focusing on lipids of the entire candidate set (*n* = 198, 396, 594, 990, 1188, 1226, 830, 948, 990, 792, 594, 594, 910, 552, and 594, respectively). **i** A similar violin plot as in (**h**) but focusing on the length of Tail 2 (*n* = 1364, 1188, 1364, 1188, 1364, 1188, 2552, and 1188, respectively). **j** A similar violin plot as in (**h**) but focusing on the lipids of the entire candidate set for the HeLa cell (*n* = 198, 196, 594, 990, 1188, 1226, 830, 948, 990, 792, 594, 594, 910, 552, and 594, respectively). The box denotes the interquartile range of predicted potency change. The mean is marked by the central dot within each box. The error bars represent the 95% confidence interval in (**g**–**j**). **k**, **l** Top two most important molecular descriptors identified by this model fine-tuned for HeLa and RAW 264.7 cells, respectively, for each headgroup. Source data are provided as a Source Data file.

The AGILE model was developed through two mechanisms: (1) identification of influential molecular descriptors using a gradient-based model interpretation method, and (2) discernment of critical features within selected lipids. We applied the gradient-based interpretation method to the input of molecular descriptors, assessing their contribution to the model’s prediction. As illustrated in Figs. 5a and 5b, we have visualized the top 20 salient descriptors for both the HeLa cells and RAW 264.7. For the HeLa cell line, VSA_EState3 and SssNH emerged as the most influential molecular characteristics for predicting mTP. VSA_EState3, a descriptor quantifying the electronic and steric properties of a molecule’s surface area within a specific range^52^, along with SssNH, representing a tertiary amine, aligning well with the understanding that tertiary amine head groups are vital for lipid design. Subsequent analysis of essential features, classified by head groups, (Fig. 5k) pinpointed PEOE and Estate as the most critical descriptors for top-performing head groups (A13, A21), while SsNH2 (Sum of sNH2 E-states) and NsNH2 (Number of atoms of type sNH2) dominated in the poorest-performing groups (A5, A17) (Supplementary Fig. S29). Notably, these descriptors are strongly associated with the amide bond in the structure, a critical connection within the 3-CR Ugi Markush structure. This connection allows for various functional group attachments, influencing the lipid-like substances’ overall charge and their physicochemical properties within biological systems. Intriguingly, the model does not favor amide bond generation, potentially due to its impact on the overall physicochemical properties of lipids, such as pKa. In the context of RAW 264.7, SpDiam_Dzi and VR3_D are identified as the most influential descriptors (Fig. 5b). VSA_EState appears as the third most influential, implying its pivotal role in determining mTP to RAW 264.7, akin to HeLa cells. Interestingly, head groups that underperformed in HeLa (A5, A17) emerged as top performers in RAW 264.7, with SsNH2 and NsNH2 remaining the most critical features. In HeLa cells, the cyclized head group outperformed the linear head group in transfection efficacy. However, the opposite trend was observed in RAW 264.7 cells. These observations underscore the necessity of designing LNPs with specific lipids tailored for distinct cellular targets.

Our subsequent analysis (Fig. 5e) explicates the relationships among lipid candidates targeting HeLa cells, as identified by similarities in the AGILE model’s lipid representations. We constructed a similarity network for the chosen 15 lipids, linking each lipid to its nearest equivalents. H9, the most potent LNP, demonstrated connections not only to LNPs with an identical head group (H7, H8) but also to other high-performing candidates, as identified by mTP (H12, H13). To gain further insights, we carried out molecular explanations on H9, illuminating the most salient regions in the molecule structure that heavily influenced the graph encoder’s prediction within the AGILE model (Fig. 5c, section “Methods”). Interestingly, head group structures emerged as the most salient for H9, which aligns with our previous findings emphasizing the importance of head groups.

Likewise, we also built a similarity network for the top 15 candidates identified for RAW 264.7 cells (Fig. 5f). R6 exhibited connections with other high-performing candidates, including R3, R8, and R11. These four lipids share identical tail structures: one being a C-12 alkyl chain and the other a C18 alkyl chain. This shared characteristic suggests a strong correlation between these tail structures and the high mTP of R6, R3, R8, and R11. Interestingly, both tails are non-biodegradable, which hints at the potential importance of lipid tail stability for successful macrophage transfection. Furthermore, these high-performing lipids commonly feature asymmetrical alkyl chains, a trait shared with SM-102, which more readily facilitates the formation of an inverted cone geometry^53^. Similar to the findings for H9, head group structures were identified as an influential factor on the saliency map for R6. Additionally, the tail end was also highlighted as a salient region (Fig. 5d).

Moreover, our results highlight the importance of the carbon chain length of Tail 1 as a critical factor in predicting mTP, particularly in RAW 264.7 cells. It presents the distribution of predicted potencies relative to varied lengths of Tail 1 for lipids hailing from the top-performing head group A5 (Fig. 5g). Two distinct aspects emerge from this distribution: (1) as the length of Tail 1 increases from 10 to 12, a corresponding uptick in predicted mTP becomes apparent. Interestingly, any further length extension in Tail 1 inversely impacts the predicted mTP. (2) In addition, Tail 1 with a shorter length (C ≤ 12) correlates with less variance in predicted mTP compared to the longer counterparts (C > 12), which is corroborated by a PCC of −0.58. This trend is not limited to the top-performing head groups but resonates across all other head groups as well (Fig. 5h and Supplementary Fig. S30). Examining the distribution plot across all head groups (Fig. 5h) reveals a similar pattern concerning the length of Tail 1 and predicted mTP, albeit with a slightly attenuated PCC of −0.39. Interestingly, the importance of carbon chain lengths varies asymmetrically between the two respective tails. The correlation between the predicted transfection potencies and the lengths of Tail 2 is noticeably lower than that observed upon lengths of Tail 1 (−0.15 vs. −0.39) (Fig. 5i and Supplementary Fig. S31). These findings pertain specifically to transfection in RAW 264.7 cells. The pattern between carbon chain lengths and the predicted mTP for HeLa cells is less defined, resulting in a Pearson correlation of −0.22 (Fig. 5j and Supplementary Figs. S32, S33). Collectively, these insights hold significant implications for guiding the design of LNPs specifically tailored for macrophages.

## Discussion

In this work, we have introduced AGILE, a platform that combines deep learning with combinatorial chemistry and is trained on a vast array of both virtual and experimental wet-lab data, to predict the mTP across different cell lines. Compared to an ML-aided lipid engineering platform we recently developed^54^, a key feature of AGILE is its initial phase of self-supervised pre-training, which ingrains the model with a nuanced understanding of an array of molecular structures. This foundational phase is vital as it exposes the model to a diverse set of molecular descriptors during its training process, thereby equipping the deep learning component of AGILE with fundamental and intricate insights into the dynamics of LNP design. This includes an in-depth analysis of features such as electronic and steric properties, and variations in carbon chain lengths, all assimilated in a self-supervised manner.

The efficacy of this method is validated through our ablation experiments, which underscore the significance of this pre-training phase in enhancing the overall accuracy of AGILE’s predictions. It’s important to note, however, that the future trajectory of AGILE’s predictive accuracy is closely linked to the continued expansion of our pre-training datasets in both scale and diversity. The more comprehensive and varied the data AGILE is trained on, the more refined and precise its predictions are likely to be.

Another critical aspect of AGILE’s functionality is its reliance on large-scale datasets for fine-tuning, which are generated through our high-throughput combinatorial lipid synthesis platform. While this reliance does impose certain limitations—given that the model’s accuracy is inherently tied to the quality and breadth of data available—it also underscores a significant advancement in addressing the challenges typically associated with developing effective deep learning models for LNPs. Notably, the training data collected from crude ionizable lipids for AI model training represents a limitation of this study. Although it has been reported that Ugi-based multi-component reactions have obtained acceptable conversion yields for HTS in vitro, purifying all ionizable lipids synthesized at the HTS stage is anticipated to improve the quality of the training data, which, in turn, enhance the precision of AI model predictions^55^. Additionally, including a filtration or dialysis step after the synthesis of LNPs using the automated liquid handler and incorporating the physicochemical characterization data of all purified LNPs in a high-throughput manner might also enhance the wet-lab dataset quality, consequently boosting the accuracy of model predictions. Besides, our combinatorial library, encompassing thousands of compounds, does not allow for DoE formulation optimizations for each compound during the HTS phase. Therefore, we initially adopted the HTS approach using a fixed standard formulation ratio, followed by a DoE-based optimization for lipids that show promise. This strategy, while efficient and widely adopted in the field, does carry an inherent risk of overlooking potentially effective lipid candidates due to the standard formulation’s possible incompatibility with certain lipids^15,16,45,56^.

Despite these inherent challenges, AGILE, after its initial self-supervised pre-training on a virtual lipid library followed by subsequent fine-tuning with extensive wet-lab data, demonstrates a strong ability to accurately predict mTP by deciphering the complex interplay between ionizable lipids’ molecular structures and their mRNA transfection capabilities. A key finding of AGILE is the significant influence of molecular descriptors such as VSA_EState3 and SssNH on mTP predictions for HeLa cell lines. These descriptors, which represent the electronic and steric properties of a molecule’s surface area and a tertiary amine respectively, resonate with current understandings in lipid design. The correlation of these molecular characteristics with their impact on mTP is a testament to the power of deep learning in unveiling subtle but crucial molecular features, thereby bridging the gap between expert knowledge and computational model interpretations. Moreover, AGILE’s analysis of the RAW 264.7 cell line identified different influential descriptors, namely SpDiam_Dzi and VR3_D, highlighting the distinct physicochemical properties preferred by different cell types. This finding is pivotal as it sheds light on the inherent complexities in crafting LNPs tailored to specific cell types. The variance in influential descriptors across different cell types underscores the inadequacy of a universal LNP design, advocating for a more nuanced, cell-specific approach in ionizable lipid design. Furthermore, AGILE’s findings illuminate the critical role played by the carbon chain length of Tail 1 in influencing the mTP of ionizable lipids, particularly in the context of RAW 264.7 cells. This insight underscores the importance of meticulous optimization of elements like Tail 1’s length to enhance transfection potency in certain cell types. The observed discrepancy in correlations between transfection potencies and the lengths of Tails 1 and 2 suggests an asymmetrical significance of the two lipid tails, reinforcing the notion that ionizable lipid design for mRNA delivery is a sophisticated, multifaceted process that depends on a complex interplay of various factors. In comparison to conventional approaches for developing ionizable lipids, AGILE has effectively tackled limitations in chemical diversity and screening efficiency. Notably, the structures of lipids H9 and R6, which were identified by AGILE, have not been previously reported.

To further elevate AGILE’s potential, it is imperative to enhance the quality of the data for training. This is particularly crucial given the discrepancies often noted between cell-based assays and in vivo results in prior studies^57^. Certain vectors may exhibit limited activity in vitro but demonstrate significant efficacy in vivo. Addressing this discrepancy necessitates the diversification of our mRNA transfection dataset with data sourced from animal models and human-derived samples like organoids. Such an expansion of the dataset is expected to markedly improve the in vivo delivery performance of ionizable lipids identified by AGILE, thereby facilitating their clinical advancement in mRNA LNP therapies. Additionally, the incorporation of a wider range of combinatorial chemistry methods^58^ and the inclusion of varied wet-lab data, such as biofunctional data, will further broaden the chemical diversity available for AGILE’s training. This strategy could empower AGILE to identify ionizable lipids with specific functionalities, such as immunostimulatory ionizable lipids for mRNA vaccine delivery and cancer immunotherapy^15,59^.

It is important to clarify that the current deep learning model in AGILE is not generative in nature as it does not create ionizable lipid structures. Instead, its strength lies in elucidating the structure–activity relationships of ionizable lipids, predicting the mTP of lipid structures, and aiding in the identification of promising lipid candidates from a vast virtual library for experimental validation in mRNA delivery. Looking ahead, the integration of advanced generative models, such as diffusion networks^60,61^, into AGILE might potentially enable the generation of ionizable lipids tailored for specific applications. In addition, we acknowledge that the current AGILE model, fine-tuned with data from 1200 lipids synthesized via the 3-CR method, may have limitations in accurately predicting the performance of lipid structures beyond those in its training set. This highlights the importance of ensuring that the training data encompass a broad representation of possible molecular configurations to maintain accuracy across diverse test scenarios. The pre-training phase is also introduced specifically in consideration to relieve the above constraint to an extent: by the self-supervised learning over vast amounts of lipid-like structures not limited to the experimented ones, the model is expected to learn more generalizable features. We indeed observed the higher accuracy of the pre-trained, fine-tuned model in comparison to the model without pre-training in our ablation experiments. For future direction, we expect the expansion of both pre-training and fine-tuning data to address this challenge more thoroughly.

In conclusion, AGILE represents a groundbreaking fusion of combinatorial chemistry and deep learning, illuminating the intricate dynamics of LNP design and broadening these insights for diverse applications. Crucially, AGILE offers a viable solution to the expensive and labor-intensive challenges associated with lipid synthesis and screening. By enhancing the likelihood of identifying effective lipids, AGILE substantially streamlines the process of developing LNPs for clinical use, significantly reducing both resource and time commitments. Furthermore, AGILE’s capacity to guide the creation of tailor-made ionizable lipids and accelerate the discovery of potent LNPs heralds an era in the development and application of mRNA-based therapeutics in various clinical settings. This work illustrates the transformative impact of integrating HTS with sophisticated computational techniques, paving the way for overcoming traditional barriers in nanomedicine research.

## Methods

Details of the chemical synthesis for the compound libraries can be found in the Supplementary Information file.

### Data preparation

#### Virtual library

We utilized the Ugi combinatorial chemistry method to design diverse head groups, connecting groups, and two distinct alkyl chains. To be specific, we used the Markush Editor in the ChemAxon Marvin Suite (Marvin 23.4.0, ChemAxon, https://www.chemaxon.com). The resulting virtual library contained ~60,000 lipid structures which were then exported into SMILES strings. This virtual library compromises multiple carbon chains, from C6 to C26. In addition, the presence or absence of ester bonds and their position in the carbon chain are used to improve the chemical diversity of the virtual library. The surface charge of LNP is usually determined by the lipids’ head groups. In addition, the head group is critical for mRNA binding. Amine groups are commonly used as lipids’ head groups to form hydrogen bonds with mRNA, especially those containing tertiary amine.

#### Experimental library

Our experimental library contains 20 head groups, 12 carbon chains with ester bonds, and 5 carbon chains with isocyanide head groups. We selected 1200 lipids for chemical synthesis and in vitro mTP experiments in HeLa and RAW 264.7 cell lines. We label the corresponding mTP in cells to each compound for the 1200 lipids library. And these data are generated by ChemAxon Marvin Suite into SMILE files (SMILE files in SI). The dataset is split based on Murcko scaffolds^62^ to ensure a robust validation of our model. This is achieved by extracting the core scaffold of each molecule in the dataset using the Murcko Scaffold method in RDkit, which can optionally include chiral centers.

#### Candidate library

The final library used for model prediction is a filtered subset of the virtual library. The filtering contains three steps based on availability and rationality. First, we retained the lipids containing tertiary amine structures. Second, we removed tail chains that were too long (>C18) or too short (<C10) based on expert knowledge of plausible ionizable lipid design^15^. Last, we select only those reagents commercially available for further validation of the model. Upon completion of the filtering process, the final candidate library comprises ~12,000 lipids (SMILE files in SI), with 22 unique head groups (Supplementary Fig. S29), and there are 9 unique tail types of Tail 1 and 2 different types of Tail 2 in this arrangement (Supplementary Fig. S34). In the prediction step of the platform, the model selects the most promising lipids based on the ranking of compounds in the candidate library.

#### Molecular graph construction

Molecular structures can be naturally represented as graphs where atoms are nodes and bonds are edges. For each molecule, the SMILES representation is converted into a molecular graph using RDKit^63^, and later input to the neural network model in the platform. This representation captures the topological structure and properties of a molecule effectively. An ionizable lipid molecule graph $G$ is defined as $G=\left(V,{E}\right)$, where nodes $V$ represent the atoms and edges $E$ represent chemical bonds. The atom node features include the atom type (as on the periodic table) and a flag indicating whether the whole molecule it belongs to is chiral. For a node $v$, the features are constructed in a two-dimensional vector, ${h}_{v}\in \,{{N}}^{2}$. Edge features are constructed based on respective chemical bond types (i.e., single, double, triple, or aromatic bonds) and the stereochemical directionality (i.e., the rdchem.BondDir) in RDKit. Similarly, the edge features form another two-dimensional vector for each bond between atom $v$ and $u$, ${\epsilon }_{v,u}\in \,{{N}}^{2}$.

#### The model architecture

The deep learning model in AGILE comprises three major components: (1) the embedding layers to project node and edge features into learnable vectors, (2) the graph encoder for modeling molecular structures, and (3) the descriptor encoder for modeling molecular properties.

#### Embedding layers

The embedding layers project the integer features in ${h}_{v}$ and ${\epsilon }_{v,u}$ to learnable feature vectors ${h}_{v}^{\left(0\right)}\,{{{\rm{and}}}}\,{\epsilon }_{v,u}^{\left(0\right)},$ which can be optimized later during the training of the whole neural network. Here, both ${h}_{v}^{\left(0\right)}\,{{{\rm{and}}}}\,{\epsilon }_{v,u}^{\left(0\right)}$ are ${R}^{d}$ vectors, and $d$ is a predefined size of embedding dimensions. To be specific, we first obtained the embedding vectors for both atom type and charity features in ${h}_{v}$, and added the two vectors elementwise to output the ${h}_{v}^{\left(0\right)}$:

1 $${h}_{v}^{\left(0\right)}={{Emb}}_{h,0}^{\left(0\right)}\left({h}_{v}\left[0\right]\right)+\,{{Emb}}_{h,1}^{\left(0\right)}\left({h}_{v}\left[1\right]\right),$$

here [i] denotes the *i*-th element in the vector. ${{{\rm{Emb}}}}$ is the embedding layer projection. In this work, we use the PyTorch embedding layers (https://pytorch.org/docs/stable/generated/torch.nn.Embedding.html). Similarly, the ${\epsilon }_{v,u}^{\left(0\right)}$ is computed as:

2 $${\epsilon }_{v,u}^{\left(0\right)}={{Emb}}_{\epsilon,0}^{\left(0\right)}\left({\epsilon }_{v,u}\left[0\right]\right)+\,{{Emb}}_{\epsilon,1}^{\left(0\right)}\left({\epsilon }_{v,u}\left[1\right]\right).$$

#### Graph encoder

We used graph isomorphism network (GIN)^64^, a type of GNN, to operate on the input molecule graphs and to learn a representation vector for each ionizable lipid molecule. GIN can directly propagate messages among nodes and edges on a graph structure and thus is suitable for processing molecular graphs. Additionally, the advantage of GIN over other GNNs is its ability to distinguish between different graph structures, including isomorphic graphs. This makes GIN more expressive than many other GNNs and a suitable tool for tasks involving molecular graph data. It is worth noting that the implemented GIN model follows similar structures used in MolCLR, so that we can benefit from the general pre-trained molecular model of MolCLR as a warm start for the platform (section “Methods”). The update rule of GIN for a node representation on the ${k}{{{\rm{th}}}}$ layer is given as:

3 $${h}_{v}^{\left(k\right)}={{MLP}}^{\left(k\right)}\left(\left(1+{\varepsilon }^{\left(k\right)}\right)\cdot \,{h}_{v}^{\left(k-1\right)}+\,{\sum }_{u\in N\left(v\right)}{m}_{u}^{\left(k-1\right)}\right),$$

where ${h}_{v}^{\left(k\right)}$ is the representation of node $v$ at the ${k}{{{\rm{th}}}}$ layer and $N\left(v\right)$ denotes the set of neighbors of node $v$, and $\varepsilon$ is a learnable parameter. MLP denotes the stacked fully connected neural network layers. The ${m}_{u}^{\left(k-1\right)}$ is the message propagated between a neighbor $u$ to the current node. It is computed as the sum of node and edge contributions:

4 $$\begin{array}{c}{m}_{u}^{\left(k-1\right)}={h}_{u}^{\left(k-1\right)}+\,{\epsilon }_{v,u}^{\left(k-1\right)},\\ {\epsilon }_{v,u}^{\left(k-1\right)}=\,{{Emb}}_{\epsilon,0}^{\left(k-1\right)}\left({\epsilon }_{v,u}\left[0\right]\right)+\,{{Emb}}_{\epsilon,1}^{\left(k-1\right)}\left({\epsilon }_{v,u}\left[1\right]\right).\end{array}$$

Notably, we use ${h}_{v}^{\left(0\right)}$ *and* ${\epsilon }_{v,u}^{\left(0\right)}$ from Eq. (1) and Eq. (2) for the first GIN layer.

We stack a total of *K* GIN layers for the entire graph encoder. To extract the feature of the whole molecular graph ${h}_{G}$, we implemented the mean pooling operation on the final layer to integrate all the node features:

5 $${h}_{G}={{Mean}}\left(\left\{{h}_{v}^{\left(K\right)}:v\,\in G\right\}\right).$$

Another fully connected layer is used to transform ${h}_{G}$ to the final lipid representation ${z}_{G}$:

6 $${z}_{G}={{MLP}}\left({h}_{G}\right).$$

#### Molecular descriptor encoder

In addition to the structure features encoded by the GIN, the platform utilizes another descriptor encoder to explicitly model molecular properties. In our experiment, we found this contributes to a more stabilized training optimization. We hypothesize that this benefit comes from the straight-forward utilization of computed properties during the optimization, which relieves the model from learning all information from the structure alone. In the implementation of the platform, the molecular descriptors derived from Mordred^32^ calculations were used, which contain over 1000 common descriptors for each molecule, including the number of atoms, bonds, etc. These features are encoded by gully connected layers into a representation for these properties, ${z}_{p}\in {R}^{{d}_{p}}$:

7 $${z}_{p}={{MLP}\, \left({descriptors}\right)}.$$

The final representation of the molecule is the concatenation of the structure and property representations:

8 $$z=\left[{z}_{G},\, {z}_{p}\right],$$

where [,] denotes the concatenation of two vectors.

#### Model pre-training

The model pre-training aims to learn generalizable lipid representation that can benefit the downstream mTP prediction task. Before our lipid-oriented pre-training, we first initialized the model parameters with the general pre-trained model from MolCLR, which has been trained on over ten million distinct small molecules. The rationale for this initialization is to provide a warm start to a model that already has been trained to capture molecular structures. Next, we perform continuous pre-training on the 60,000 lipids in the virtual library (section “Methods”) using contrastive learning to optimize the model’s performance within the lipid domain.

#### Contrastive learning objective

Our pre-training objective is to learn ionizable lipid representation through contrasting positive data pairs against negative pairs. The model is trained to minimize the following loss:

9 $$\begin{array}{c}{L}_{i,j}=-log \frac{{{{\rm{ex}}}}{{{\rm{p}}}}\left(\frac{{sim}\left({z}_{i},\, {z}_{j}\right)}{\tau }\right)}{{\sum }_{k=1}^{2N}{\mathbb{l}}\left\{k\ne i\right\}{{{\rm{ex}}}}{{{\rm{p}}}}\left(\frac{{sim}\left({z}_{i},\, {z}_{k}\right)}{\tau }\right)},\\ {sim}\left({z}_{i},\, {z}_{j}\right)=\frac{{z}_{i}{z}_{j}}{{{{\rm{||}}}}{z}_{i}{{{{\rm{||}}}}}_{2}{{||z}}_{j}{{||}}_{2}},\end{array}$$

where ${z}_{i}$ and ${z}_{j}$ are the learned lipid representation vectors extracted from a positive data pair, $N$ is the batch size, and $\tau$ is the temperature parameter set manually. In this pre-training step, we omitted the descriptor encoder, so the lipid representation only contains the graph structure representation ${z}_{G}$ as in Eq. (6). To construct the positive data pair, each input lipid molecule graph is transformed into two different but correlated molecule graphs using graph augmentation. The molecule graphs augmented from the same molecule are denoted as a positive pair, and those from different molecules are denoted as negative pairs within each batch. During training, the model learns to maximize the agreement of positive pairs while minimizing the agreement of negative ones.

### Data augmentation

We used two augmentation strategies inherited from the MolCLR pre-training workflow at the atom and bond levels. In the continuous pre-training of lipid molecules, three molecular graph data augmentation strategies are consistently employed. (1) Atom masking: within the lipid molecular graph, atoms are randomly masked according to a specified ratio. This process compels the model to assimilate chemical information, such as atom types and corresponding chemical bond varieties within lipid molecules. (2) Bond deletion: chemical bonds interconnecting atoms are randomly removed in accordance with a designated ratio. As the formation and dissociation of chemical bonds dictate the properties of ionizable lipid molecules during chemical reactions, bond deletion facilitates the model’s learning of correlations between ionizable lipid molecule involvement in various reactions.

### Model fine-tuning

The lipid-oriented pre-trained model (section “Methods”) serves as the starting point of the fine-tuning stage. During the fine-tuning, we included the molecular descriptor encoder and used the combined output $z$ in Eq. (8) as the molecule representation. For the property descriptor input, a series of preprocessing procedures are executed, aiming to isolate pertinent features. Initially, descriptors with a standard deviation of zero are eliminated, followed by the selection of descriptors exhibiting correlation with the experimentally determined mTP in both HeLa and RAW 264.7 cells (score of *R*^2^ > 0.006), resulting in the identification of 813 salient descriptors (Supplementary Fig. S35). Subsequently, log transformation is applied to descriptors possessing extensive data ranges, with normalization conducted accordingly. The preprocessing steps enacted on the fine-tuning dataset are documented and replicated for the 12,000 lipids in the candidate library in anticipation of the model prediction phase (section “Methods”).

The model is fine-tuned utilizing the 1200 lipids of the experiment library to perform regression on mTP. The mean squared loss between the predicted and ground-truth potency is used to optimize the model parameters:

10 $${L}_{{mse}}=\frac{1}{n}{\sum }_{i=1}^{n}{\left({Pred}\left({z}_{i}\right)-{y}_{i}\right)}^{2},$$

where ${Pred}\left(\cdot \right)$ denotes the fully connected layers that perform the mTP prediction, and ${y}_{i}$ is the actual mTP recorded in vitro.

A scaffold-based 80%–10%–10% train–valid–test split is performed on the experimental library. We fine-tune the model on the training set only and evaluate the performance on the validation set using root mean squared error (RMSE) and Pearson correlation with the ground-truth mTP.

### Model ensemble prediction and candidate ranking

To enhance the model’s robustness and generalizability, the fine-tuning process is carried out ten times, from which the top five models are selected based on RMSE and Pearson correlation performance on the testing set. These five models are subsequently employed for ensemble prediction on the 12,000-member candidate set. We first get the mTP predictions from each model and calculate the average and standard deviation of the five predicted values for each candidate molecule. The mean predicted values are then subtracted from the standard deviation, and the resulting predicted score is used to rank the candidates. We observed that the predicted potencies exhibit distinct stratification based on combinations of headgroups and tails, and the predicted mTP differences between molecules with the same headgroups and similar tails are relatively minor (Supplementary Fig. S36). To increase the diversity of selected candidates, we implement a ranking scheme that sorts candidate lipids by headgroups and tail combinations (Supplementary Fig. S37). Given the predicted values, candidates are first organized by headgroups and subsequently ranked in descending order. Candidates within each head group are then ranked by tail combinations following the same schema. Ultimately, we select the top five head groups and the top three tail combinations from each headgroup, resulting in a final candidate set of 15 lipids.

### Implementation details

The graph encoder in the model consists of a five-layer GIN with ReLU activation. To extract a 512-dimensional lipid representation, an average pooling layer is applied to each lipid molecular graph. A single hidden layer MLP is then employed to map the representation into a 256-dimensional latent space. During model pre-training, the contrastive loss is optimized using the Adam optimizer^65^, with a weight decay of 10^−5^, and the temperature is set to 0.1. The pre-training process involves a batch size of 512 for 100 epochs.

For model fine-tuning, an additional MLP with one hidden layer is introduced to map the molecular descriptors into 100-dimensional latent vectors. These vectors are concatenated with the 256-dimensional lipid representation obtained from the GNN encoder. Subsequently, a two-layer MLP is utilized to derive the final prediction value from the concatenated vector. The fine-tuning process employs the Adam optimizer with a weight decay of 10^−6^ to optimize the loss (Eq. (10)). Each fine-tuned model is trained using a batch size of 128 for 30 epochs.

### Comparison to other methods

To assess the precision and reliability of predicted mTP, AGILE was benchmarked against traditional ML algorithms, including Ridge regression^38^, Lasso^39^, Gradient Boosting^40^, and SVM^41^. To ensure the fairness of comparison, all models were trained and tested using the mTP results derived from the wet-lab experiment involving the 1200 LNPs in HeLa cells. Specifically, we allocated 80% of the data for model training, 10% for optimal hyperparameter selection, and the remaining 10% for result evaluation. Notably, we only used molecular feature descriptors as the input for the above-mentioned traditional ML algorithms since they are not able to process molecular structure data. We used *R*^2^ and PCC as metrics to evaluate the models’ robustness and accuracy. These evaluation results are included in Supplementary Table 5. AGILE outperforms other methods with the highest *R*^2^ and PCC scores of 0.249 and 0.573, respectively. In contrast, Ridge struggles with *R*^2^ of −1.035 and shows PCC of 0.514. SVM has *R*^2^ of 0.07 and PCC of 0.409. Gradient boosting has *R*^2^ of 0.09 and PCC of 0.308. Lasso performs with a lower *R*^2^ of −0.01 and PCC of 0.04. AGILE’s outperforming performance can be attributed to the combined representation of both lipid structures and molecular descriptors, making it more generalizable and robust for this intricate task of mTP prediction. The overall modest scores across models underscore the inherent challenges of the task, yet AGILE demonstrates great potential given its superior performance.

### Model interpretation

#### Salient molecular descriptors calculation

In our study, we employed the Integrated Gradients^66^ methodology featured in the Captum^67^ Python package to interpret the significance of molecular descriptors. The process involves approximating the integral of molecular descriptor gradients in relation to their respective predicted mTP for each ionizable lipid within the candidate library. A molecular descriptor’s prominence is proportionate to the absolute value of its integrated gradient. We implemented computations across all five ensemble models for each target cell line. To calculate an overall significance for each feature, we initially averaged the computed gradients across all input samples on each model, subsequently normalizing these important scores. The final step involved computing the mean of these important scores across all five models. The top 20 critical features were selected and visualized based on the calculated importance scores. When assessing feature significance in the context of headgroups, we averaged the integrated gradients for each headgroup and then proceeded to normalization. Following this, we averaged the results across the five models for each respective headgroup. The top two significant features for each headgroup were then selected, and their scores were visualized across all headgroups.

### Construction of the similarity network on the selected candidates

We constructed a similarity network for the 15 selected candidates respective to each target cell line, with the aim of elucidating the similarities among the candidates. Utilizing the vector representations provided by the corresponding fine-tuned model, we computed the cosine similarities for each candidate pair and chose the four most similar neighbors for each. This generated similarity network was then visualized, with the node sizes representing the relative luciferase units.

### Molecular structure interpretation

To ascertain the critical areas within the lipid structure that contribute significantly to the model’s predictions, we engaged the Model Agnostic Counterfactual Compounds Generation feature present in the ExMol Python package^68^. This is accomplished by generating molecular counterfactuals and investigating the alterations required in the lipid molecule to modify its predicted mTP (Supplementary Fig. S38). The molecular counterfactuals produced are designed to retain as much similarity to the input lipid molecule as feasible. If modifications in particular regions result in either an increase or decrease in the mTP, such areas are deemed essential regions. The critical areas identified through this process were visualized for both H9 and R6.

### Materials and lipid library synthesis

All materials were prepared and processed without nucleases throughout the synthesis and formulation steps. mFLuc (Translate), Cre recombinase mRNA (TriLink BioTechnologies), mOVA (TriLink BioTechnologies), and EGFP-mRNA (TriLink BioTechnologies) were directly purchased from vendors. PrestoBlue™ Cell Viability Reagent and Quant-it™ RiboGreen RNA Assay Kit were purchased from Thermo Fisher Scientific Inc. All mRNAs were stored at −80 °C and were allowed to thaw on ice before use. DLin-MC3-DMA and ALC-0315 were purchased from Echelon Biosciences. Amine headgroups and starting compounds were purchased from Sigma-Aldrich and TCI America to synthesize ionizable lipids. The tails were purified through flash column chromatography, and their final structures were confirmed using ^1^H 400 MHz NMR spectrometry with CDCl_3_ and tetramethylsilane as a standard at UHN Nuclear Magnetic Resonance Core Facility. To synthesize compounds in each well of a 96-well plate with glass inserts, we added 10 μL from a stock solution containing amines, tails, and catalyst that had been mixed and pre-stirred overnight. Specifically, the 350 μM stock solution was prepared by combining the amine and tail components in a 1:1:1 ratio and dissolving them in a 2:1 mixture of methanol and 0.2 equivalents of the phenyl hypophosphoric acid catalyst. This stock solution was then added to each well of the 96-well plate. The covered plates were placed on a shaker and stirred overnight to allow the reactions to proceed. To further analyze our materials, we obtained high-resolution mass spectra using an LC-Mass spectrophotometer at the Centre for Pharmaceutical Oncology of the University of Toronto.

### LNP formulation and characterization

Before preparing LNPs, we estimated the average final concentration of ionizable lipids after the final reaction was completed and used the molar ratio of ionizable lipids/DOPE/Chol/C14-PEG2000 was 35/16/46.5/2.5 to formulate LNPs based on previous work^69^.

In HTS, LNPs were created by employing an automated liquid handler (OT-2) to mix an aqueous phase with an ethanol phase at a volume ratio of 3:1. The ethanol phase incorporated a crude mixture of ionizable lipids, DOPE (Avanti), cholesterol (Chol, Sigma-Aldrich), and C14-PEG2000 (Avanti), dissolved in ethanol at a pre-established molar ratio. Concurrently, the aqueous phase was formulated in a 10 mM citrate buffer including mFluc. At the high-throughput screening stage, the synthesized ionizable lipids were not purified before LNP formulation and the concentrations of crude lipids were estimated based on average reaction yields obtained from preliminary studies^15,16,45,56^.

For additional in vitro and in vivo HTS, LNPs were generated by manual pipetting to mix an aqueous phase with an ethanol phase, keeping the same 3:1 volume ratio. The aqueous phase was composed in a 10 mM citrate buffer containing the relevant mRNA. The ethanol phase involved a mixture of ionizable lipid and helper phospholipids (DOTAP, DOPE, cholesterol, and C14-PEG2000), dissolved at pre-determined molar ratios maintaining an ionizable lipid/mRNA weight ratio of 10:1. MC3-LNP and ALC-0315-LNP were formulated at molar ratios of 50:10:38.5:1.5 (MC3:DSPC:cholesterol:DMG-PEG2000) and 46.3:9.4:42.7:1.6 (ALC-0315:DSPC:cholesterol:ALC0159 [Echelon Biosciences]), respectively.

For in vitro and in vivo studies, excluding the high-throughput screening phase, LNPs were dialyzed against 1× PBS in a 20,000 MWCO cassette (Thermo Fisher) at 4 °C for 6 h before testing in cells and animals. During high-throughput screening, LNPs prepared using an automated liquid handler (OT2) were directly subjected to cellular assays without additional dialysis steps. The optimization of H9 and R6 LNP formulations for subsequent experiments was achieved using a DoE approach. This process involved the use of JMP 16 statistical software (SAS Institute) to analyze the experimental data. The application of a four-factor Box-Behnken design facilitated the development of second-order models across 17 preparation runs. This approach is widely acknowledged as an effective experimental design for the identification of key factors. The design encompassed five factors: lipid/mRNA weight ratio, ionizable lipid molar ratio, helper lipid or cationic molar ratio, PEG molar ratio, and cholesterol molar ratio, each of which was examined at low, middle, and high levels. Before formulation optimization, H9 LNP and R6 LNP were formulated with the same ratio of 35/16/46.5/2.5 (ionizable lipid:DOTAP:cholesterol:DMG-PEG2000). The top-performing H9 LNP and R6 LNP were formulated at molar ratios of 50:10:38.5:1.5 (H9:DOPE:cholesterol:DMG-PEG2000) and 60:15:42.7:1.6 (R6:DOTAP:cholesterol:DMG-PEG2000), respectively. The size, polydispersity index (PDI) and zeta potentials of LNPs were measured using Zetasizer Nano ZS (Malvern Instruments). mRNA encapsulation efficiency (EE) was measured by Ribogreen assay as previously described^70^. Briefly, a 100 mM stock solution of citric acid, sodium monobasic phosphate, and sodium bicarbonate was prepared. Using a 1 M sodium hydroxide stock solution, each buffer stock was aliquoted to create a total of 16 individual buffers with pH values ranging from 2 to 11. Citrate buffers ranged from pH 2 to 6, sodium phosphate buffers from pH 6 to 8, and bicarbonate buffers from pH 8 to 11. Separately, a stock solution of TNS in water was prepared at 600 µM, and LNPs were prepared at a concentration of 0.1 mg/ml mRNA. In a black 96-well plate, 100 µl of buffer, 10 µl of LNP, and 2 µl of TNS stock were added to each well. The fluorescence of each well was measured with excitation and emission wavelengths of 325 and 435 nm, respectively. The half-maximal point of the resulting fluorescence vs. pH plot was calculated as the LNP pKa.

### LNP cytotoxicity and stability assay

To evaluate the cytotoxicity of two different post-DoE LNP formulations, H9 LNP and R6 LNP (0.1 µg) were tested in HeLa cells. For the toxicity assay, HeLa cells were seeded in 96-well plates at a density of 10,000 cells/well and incubated overnight to allow attachment. After 6, 12, 24, and 48 h, 20 μl of cell culture supernatant was added with 180 μl of QUANTI-Blue™ Solution was added to each well. Plates were then incubated at 37 °C for 15 min to allow metabolic reduction of resazurin by viable cells. The optical density value at 600 nm was immediately read on a Cytation microplate reader at 630 nm to evaluate the relative cytotoxicity induced by each empty LNP.

To study post-DoE LNP stability, the sizes, PDI, and EE were monitored for 1 week during storage in PBS at −20 °C. Similar to the storage condition of the lipid used by the Pfizer-BioNTech Comirnaty COVID-19 vaccine, 10% sucrose was added to the H9 LNP solution before the stability test. The luminescence was measured 6 h after injection.

### In vitro high-throughput screening

Freshly prepared LNPs containing 0.1 μg of mFLuc, were added to pre-seeded HeLa and Raw 264.7 cells in 96-well plates. Following overnight incubation, the transfection of mFLuc was measured using the One-Glo Luciferase Assay System (Promega), following the manufacturer’s instructions. The luminescence was quantified using the Cytation imaging reader (BioTek). The mTP value is a measure of how effectively mRNA is able to transfect cells. It is calculated as the base 2 logarithm of the ratio of mean luminescence intensity between transfected cells and untreated cells at 24-h post-treatment. Specifically, the mTP value is defined as:

11 $${{mTP}}={{Log}}_{2}\left(\frac{{{Mean}}\; {{luminescence}}\; {{intensity}}\; {{of}}\; {{transfected}}\; {{cells}}}{{{Mean}}\; {{luminescence}}\; {{intensity}}\; {{of}}\; {{untreated}}\; {{cells}}}\right)$$

To summarize, all LNPs used in the HTS were prepared using liquid handling systems without any purification. The remaining LNPs were purified and dialysis. Finally, the resulting bioluminescence values are assigned to each SMILE string.

### Animals experiment

All animal studies were approved and conducted in compliance with the University Health Network Animal Resources Centre guidelines (AUP#: 6842). Female and male C57BL/6 and ROSA^*mT*/*mG*^ Cre reporter mice (4–8 weeks) were purchased from the Jackson Laboratory. The mice were maintained in a controlled environment with a 12-h light/dark cycle. The ambient temperature was maintained at 22–24 °C, and the humidity level was kept at 40–60%. Each cage housed a maximum of five mice to maintain appropriate social interaction and minimize stress levels among the animals.

### In vivo luciferase mRNA for bioluminescence

At 6 h after the IM administration of the mRNA LNPs, mice were injected intraperitoneally with 0.2 ml d-luciferin (10 mg/ml in PBS). The mice were anesthetized in a ventilated anesthesia chamber with 1.5% isoflurane in oxygen and imaged 10 min after the injection with an in vivo imaging system (IVIS, PerkinElmer). Luminescence and fluorescence imaging were quantified using the Living Image software (PerkinElmer). For the bioluminescence assay, the exposure time (10 s), binning (medium), f/stop (1), and excitation (not applicable) emission filters (560 nm). For fluorescence imaging assay, the exposure time (30 s), binning (medium), f/stop (1), excitation wavelength 568 nm with emission filter 580 nm for GFP, excitation wavelength 488 nm with emission filter 505 nm for tdTomato, excitation wavelength 647 nm with emission filter 660 nm for Cy5. C57BL/6 mice (*n* = 3/group, 4–8 weeks, female) were purchased from the Jackson Laboratories.

### ROSA^*mT*/*mG*^ Cre reporter mice transfection analysis

For gene recombinant Cre mRNA delivery, LNPs co-formulated with Cre mRNA (0.5 mg kg^−1^) were IM injected into ROSA^*mT*/*mG*^ Cre reporter mice (*n* = 3/group, 4–8 weeks, female, from the Jackson Laboratory). After 7d, mice were killed, and major organs were collected and imaged using an IVIS imaging system (PerkinElmer). For direct fluorescence imaging, organs and muscle tissues were fixed in 4% buffered paraformaldehyde overnight at 4 °C, then equilibrated in 30% sucrose overnight at 4 °C before freezing in OCT. Three nonconsecutive sections from each organ sample were mounted with DAPI to visualize nuclei and imaged for DAPI, tdTomato, and GFP. Sectioned into 10 μm depth, and further imaged using a Fluorescence microscope (Zeiss AXIO Observer 7 Inverted LED Fluorescence Motorized Microscope).

### Transfection test in RAW 264.7 cells

LNPs containing 500 ng GFP-mRNA were added to 24-well plates pre-seeded with RAW 264.7 macrophages for 48 h incubation at 37 °C. A fluorescence microscope (Zeiss AXIO Observer 7 Inverted LED Fluorescence Motorized Microscope) and Flow cytometer (cytoFLEX S) were used to evaluate the GFP expression.

### Statistical analysis

The data were subjected to statistical analyses using GraphPad Prism 9 (GraphPad Software). A two-tailed unpaired Student’s *t*-test was conducted to assess the significance of the comparisons as indicated. Data are expressed as mean ± s.d. *P* values < 0.05 (*), *P* < 0.01 (**), *P* < 0.001 (***), and *P* < 0.0001 (****) were statistically significant.

### Reporting summary

Further information on research design is available in the Nature Portfolio Reporting Summary linked to this article.

## Supplementary information

Supplementary Information

Peer Review File

Reporting Summary

## Source data

Source Data

## Data Availability

The data generated in this study are provided in the Supplementary Information/Source Data file. Source data are provided with this paper.

## Code availability

The codebase data and code used in this study are available in the Zenodo repository^71^ (https://zenodo.org/records/21422889 and https://zenodo.org/records/11556457) and Github repository (https://github.com/bowang-lab/AGILE) with the MIT License.
