# Supplementary material for: AGILE platform: a deep learning powered approach to accelerate LNP development for mRNA delivery
Source: Nat Commun. 2024 Jul 26;15:6305. doi: 10.1038/s41467-024-50619-z (PMC11282250; doi:10.1038/s41467-024-50619-z)
Supplement: Supplementary file 1 — Supplementary Information [file 41467_2024_50619_MOESM1_ESM.pdf]

Supplementary Note 1.

Lipid Tails Synthesis for High-throughput Screening (1,200)

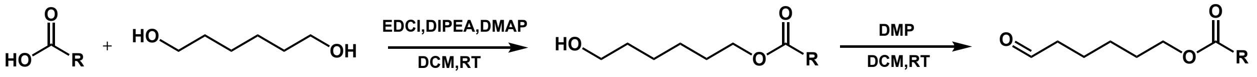

1’ 2’ 3’ 4’

General Synthesis Route of Tail A

Acid (17.7 mmol), *N*-(3-dimethyl aminopropyl)- *N′*-ethyl carbodiimide hydrochloride (EDC HCl, 26.6 mmol), hexane-1,6-diol (88.5 mmol), 4-(dimethylamino) pyridine (DMAP, 8.9 mmol) and *N, N*-diisopropylethylamine (DIPEA, 35.4 mmol) were dissolved in dichloromethane (200 ml). The reaction was stirred at room temperature under nitrogen for 18 h, then washed with a saturated aqueous sodium bicarbonate solution. The organic layer was separated, washed with brine, dried over Na2SO4, filtered, and the filtrate was evaporated under a vacuum. The residue was purified by silica gel chromatography (0–50% ethyl acetate in hexanes) to give compound **3’**. **3’** (15.2 mmol) was dissolved in dichloromethane 300 ml followed by Dess-Martin Periodinane (9.6 g, 22.8 mmol). The reaction was stirred under nitrogen at room temperature for 2 h. After confirmation of reaction completion by thin layer chromatography, sodium thiosulfate pentahydrate (50% w/v, 200 ml) was added to the reaction and left to stir for an additional 15 mins. The organic layer was thereafter separated, washed with brine, dried over Na_2SO_4, filtered, and the filtrate was evaporated under a vacuum. The residue was purified by silica gel chromatography (0–50% ethyl acetate in hexanes) to give compound **4’**.

<u>Synthesis of Tail 3’</u>

<u>Synthesis of Tail 6-hydroxyhexyl nonanoate</u>

Follow the synthesis method above. ^{1}H NMR (400 MHz, $CDCl_3$) δ 4.02 (t, *J* = 6.7 Hz, 2H), 3.59 (t, *J* = 6.6 Hz, 2H), 2.25 (t, *J* = 7.6 Hz, 2H), 1.67 – 1.47 (m, 6H), 1.35 (p, *J* = 4.0 Hz, 4H), 1.30 – 1.13 (m, 10H), 0.91 – 0.76 (m, 3H).

<u>Synthesis of Tail 6-hydroxyhexyl 4-methylnonanoate</u>

Follow the synthesis method above. ^{1}H NMR (400 MHz, $CDCl_3$) δ 4.02 (dd, *J* = 7.2, 6.3 Hz, 2H), 3.59 (t, *J* = 6.6 Hz, 2H), 2.36 – 2.12 (m, 2H), 1.64 – 1.47 (m, 5H), 1.40 – 1.15 (m, 13H), 1.10 – 0.71 (m, 6H).

<u>Synthesis of Tail 6-hydroxyhexyl decanoate</u>

Follow the synthesis method above. ^{1}H NMR (400 MHz, $CDCl_3$) δ 4.02 (t, *J* = 6.7 Hz, 2H), 3.58 (t, *J* = 6.6 Hz, 2H), 2.24 (t, *J* = 7.6 Hz, 2H), 1.73 – 1.46 (m, 6H), 1.39 – 1.31 (m, 4H), 1.26 – 1.17 (m, 11H), 0.85 – 0.80 (m, 3H).

<u>Synthesis of Tail 6-hydroxyhexyl undec-2-enoate</u>

Follow the synthesis method above. ^{1}H NMR (400 MHz, $CDCl_3$) δ 6.92 (dt, *J* = 15.6, 7.0 Hz, 1H), 5.87 – 5.41 (m, 1H), 4.05 (dt, *J* = 19.2, 6.7 Hz, 2H), 3.58 (t, *J* = 6.6 Hz, 2H), 2.15 (qd, *J* = 7.1, 1.6 Hz, 2H), 1.72 – 1.46 (m, 4H), 1.45 – 1.31 (m, 6H), 1.25 – 1.21 (m, 6H), 0.94 – 0.69 (m, 3H).

<u>Synthesis of Tail 6-hydroxyhexyl (*E*)-dec-2-enoate</u>

Follow the synthesis method above. ^{1}H NMR (400 MHz, $CDCl_3$) δ 6.91 (dt, *J* = 15.7, 7.0 Hz, 1H), 5.76 (dt, *J* = 15.6, 1.6 Hz, 1H), 4.04 (dt, *J* = 23.5, 6.7 Hz, 2H), 3.58 (t, *J* = 6.6 Hz, 2H), 2.27 – 2.06 (m, 2H), 1.76 – 1.46 (m, 5H), 1.45 – 1.30 (m, 6H), 1.24 – 1.19 (m, 6H), 0.89 – 0.76 (m, 3H).

<u>Synthesis of Tail 6-hydroxyhexyl undecanoate</u>
Follow the synthesis method above. ^{1}H NMR (400 MHz, $CDCl_3$) δ 4.02 (t, *J* = 6.7 Hz, 2H), 3.59 (t, *J* = 6.6 Hz, 2H), 2.25 (t, *J* = 7.6 Hz, 2H), 1.70 – 1.45 (m, 6H), 1.41 – 1.31 (m, 4H), 1.30 – 1.13 (m, 14H), 0.93 – 0.71 (m, 3H).

<u>Synthesis of Tail 6-hydroxyhexyl undec-10-ynoate</u>
Follow the synthesis method above. ^{1}H NMR (400 MHz, $CDCl_3$) δ 4.02 (t, *J* = 6.7 Hz, 2H), 3.59 (t, *J* = 6.6 Hz, 2H), 2.25 (t, *J* = 7.5 Hz, 2H), 2.13 (td, *J* = 7.1, 2.7 Hz, 2H), 1.90 (t, *J* = 2.6 Hz, 1H), 1.67 – 1.42 (m, 8H), 1.40 – 1.15 (m, 12H).

<u>Synthesis of Tail 6-hydroxyphenyl undec-10-enoate</u>
Follow the synthesis method above. ^{1}H NMR (400 MHz, $CDCl_3$) δ 5.74 (ddt, *J* = 16.9, 10.2, 6.7 Hz, 1H), 5.04 – 4.77 (m, 2H), 4.00 (t, *J* = 6.7 Hz, 2H), 3.56 (t, *J* = 6.6 Hz, 2H), 2.23 (t, *J* = 7.5 Hz, 2H), 1.97 (dt, *J* = 8.0, 6.7 Hz, 2H), 1.70 – 1.43 (m, 6H), 1.41 – 0.97 (m, 14H).

<u>Synthesis of Tail 6-hydroxyhexyl palmitate</u>
Follow the synthesis method above. ^{1}H NMR (400 MHz, $CDCl_3$) δ 4.06 (t, *J* = 6.7 Hz, 2H), 3.64 (t, *J* = 6.5 Hz, 2H), 2.28 (t, *J* = 7.6 Hz, 2H), 1.60 (dddd, *J* = 14.5, 12.9, 6.8, 3.2 Hz, 6H), 1.38 (p, *J* = 3.3 Hz, 4H), 1.25 (s, 22H), 0.97 – 0.76 (m, 3H).

<u>Synthesis of Tail 6-hydroxyhexyl stearate</u>
Follow the synthesis method above. ^{1}H NMR (400 MHz, $CDCl_3$) δ 4.04 (t, *J* = 6.7 Hz, 2H), 3.62 (t, *J* = 6.6 Hz, 2H), 2.27 (t, *J* = 7.6 Hz, 2H), 1.59 (dddd, *J* = 15.1, 9.8, 6.7, 3.7 Hz, 7H), 1.37 (p, *J* = 3.8, 3.3 Hz, 4H), 1.23 (s, 28H), 0.90 – 0.77 (m, 3H).

<u>Synthesis of Tail 6-hydroxyhexyl oleate</u>
Follow the synthesis method above. ^{1}H NMR (400 MHz, $CDCl_3$) δ 5.46 – 5.21 (m, 2H), 4.04 (t, *J* = 6.7 Hz, 2H), 3.62 (t, *J* = 6.6 Hz, 2H), 2.27 (t, *J* = 7.6 Hz, 2H), 2.06 – 1.85 (m, 4H), 1.63 – 1.53 (m, 6H), 1.41 – 1.34 (m, 4H), 1.33 – 1.16 (m, 20H), 0.97 – 0.79 (m, 3H).

<u>Synthesis of Tail 6-hydroxyhexyl (9Z,12Z)-octadeca-9,12-dienoate</u>
Follow the synthesis method above. ^{1}H NMR (400 MHz, $CDCl_3$) δ 5.52 – 5.12 (m, 4H), 4.03 (t, *J* = 6.7 Hz, 2H), 3.60 (t, *J* = 6.6 Hz, 2H), 2.85 – 2.58 (m, 2H), 2.26 (t, *J* = 7.6 Hz, 2H), 2.02 (q, *J* = 6.9 Hz, 4H), 1.64 – 1.50 (m, 6H), 1.39 – 1.21 (m, 18H), 0.90 – 0.77 (m, 3H).

<u>Synthesis of Tail 4'</u>
<u>Synthesis of Tail 6-oxohexyl nonanoate</u>
Follow the synthesis method above. ^{1}H NMR (400 MHz, $CDCl_3$) δ 9.76 (t, *J* = 1.7 Hz, 1H), 4.05 (t, *J* = 6.6 Hz, 2H), 2.57 – 2.07 (m, 4H), 1.75 – 1.49 (m, 6H), 1.46 – 1.33 (m, 2H), 1.32 – 1.18 (m, 11H), 0.91 – 0.80 (m, 3H).

Synthesis of Tail 6-oxohexyl 4-methylnonanoate

Follow the synthesis method above. ^{1}H NMR (400 MHz, $CDCl_3$) δ 9.76 (t, J = 1.7 Hz, 1H), 4.05 (t, J = 6.6 Hz, 2H), 2.48 – 2.26 (m, 4H), 1.69 – 1.54 (m, 6H), 1.40 (tdd, J = 8.2, 6.5, 4.5 Hz, 4H), 1.29 – 1.21 (m, 8H), 0.86 (td, J = 6.6, 1.3 Hz, 6H).

Synthesis of Tail 6-oxohexyl decanoate

Follow the synthesis method above. ^{1}H NMR (400 MHz, $CDCl_3$) δ 9.74 (t, J = 1.7 Hz, 1H), 4.04 (t, J = 6.6 Hz, 2H), 2.55 – 2.14 (m, 4H), 1.62 (ddq, J = 14.3, 9.9, 7.3 Hz, 6H), 1.43 – 1.13 (m, 14H), 0.92 – 0.78 (m, 3H).

Synthesis of Tail 6-oxohexyl undec-2-enoate

Follow the synthesis method above. ^{1}H NMR (400 MHz, $CDCl_3$) δ 9.76 (t, J = 1.7 Hz, 1H), 6.95 (dt, J = 15.6, 7.0 Hz, 1H), 5.79 (dt, J = 15.6, 1.6 Hz, 1H), 4.12 (t, J = 6.6 Hz, 2H), 2.48 – 2.25 (m, 3H), 2.18 (qd, J = 7.1, 1.6 Hz, 2H), 1.68 – 1.62 (m, 4H), 1.45 – 1.35 (m, 5H), 1.26 – 1.23 (m, 6H), 0.86 (d, J = 7.1 Hz, 3H).

Synthesis of Tail 6-oxohexyl (E)-dec-2-enoate

Follow the synthesis method above. ^{1}H NMR (400 MHz, $CDCl_3$) δ 9.82 – 9.66 (m, 1H), 6.93 (dt, J = 15.6, 6.9 Hz, 1H), 5.77 (dt, J = 15.6, 1.8 Hz, 1H), 4.09 (t, J = 6.6 Hz, 2H), 2.43 (td, J = 7.3, 1.7 Hz, 2H), 2.16 (qd, J = 7.3, 1.6 Hz, 2H), 1.71 – 1.61 (m, 4H), 1.46 – 1.35 (m, 4H), 1.26 – 1.18 (m, 6H), 0.84 (t, J = 6.7 Hz, 3H).

Synthesis of Tail 6-oxohexyl undecanoate

Follow the synthesis method above. ^{1}H NMR (400 MHz, $CDCl_3$) δ 9.74 (t, J = 1.7 Hz, 1H), 4.04 (t, J = 6.6 Hz, 2H), 2.43 (td, J = 7.3, 1.7 Hz, 2H), 2.26 (t, J = 7.6 Hz, 2H), 1.61 (ddt, J = 16.2, 9.8, 7.3 Hz, 6H), 1.42 – 1.34 (m, 2H), 1.24 (d, J = 9.2 Hz, 14H), 0.87 – 0.82 (m, 3H).

Synthesis of Tail 6-oxohexyl undec-10-ynoate

Follow the synthesis method above. ^{1}H NMR (400 MHz, $CDCl_3$) δ 4.02 (t, J = 6.6 Hz, 2H), 2.41 (td, J = 7.3, 1.7 Hz, 1H), 2.32 (t, J = 7.5 Hz, 1H), 2.24 (t, J = 7.5 Hz, 2H), 2.13 (td, J = 7.1, 2.7 Hz, 2H), 1.90 (t, J = 2.7 Hz, 1H), 1.65 – 1.55 (m, 6H), 1.52 – 1.43 (m, 2H), 1.39 – 1.31 (m, 4H), 1.27 – 1.17 (m, 6H).

Synthesis of Tail 6-oxohexyl undec-10-enoate

Follow the synthesis method above. ^{1}H NMR (400 MHz, $CDCl_3$) δ 9.76 (t, J = 1.7 Hz, 1H), 5.79 (ddt, J = 16.9, 10.2, 6.7 Hz, 1H), 5.07 – 4.79 (m, 2H), 4.06 (t, J = 6.6 Hz, 2H), 2.45 (td, J = 7.3, 1.7 Hz, 2H), 2.28 (t, J = 7.5 Hz, 2H), 2.08 – 1.95 (m, 2H), 1.71 – 1.55 (m, 6H), 1.45 – 1.37 (m, 2H), 1.36 – 1.12 (m, 9H).

Synthesis of Tail 6-oxohexyl palmitate

Follow the synthesis method above. ^{1}H NMR (400 MHz, $CDCl_3$) δ 9.76 (t, J = 1.6 Hz, 1H), 4.05 (t, J = 6.6 Hz, 2H), 2.49 – 2.34 (m, 2H), 2.28 (t, J = 7.5 Hz, 2H), 1.72 – 1.56 (m, 6H), 1.39 (tt, J = 9.8, 6.3 Hz, 2H), 1.24 (s, 22H), 0.87 (t, J = 6.7 Hz, 3H).

Synthesis of Tail 6-oxohexyl stearate

Follow the synthesis method above. ^{1}H NMR (400 MHz, $CDCl_3$) δ 9.76 (t, *J* = 1.7 Hz, 1H), 4.05 (t, *J* = 6.6 Hz, 2H), 2.44 (td, *J* = 7.3, 1.7 Hz, 2H), 2.28 (t, *J* = 7.6 Hz, 2H), 1.72 – 1.53 (m, 6H), 1.48 – 1.06 (m, 32H), 0.91 – 0.78 (m, 3H).

Synthesis of Tail 6-oxohexyl oleate

Follow the synthesis method above. ^{1}H NMR (400 MHz, $CDCl_3$) δ 9.75 (t, *J* = 1.7 Hz, 1H), 5.37 – 5.27 (m, 2H), 4.04 (t, *J* = 6.6 Hz, 2H), 2.43 (td, *J* = 7.3, 1.7 Hz, 2H), 2.27 (t, *J* = 7.6 Hz, 2H), 1.99 (q, *J* = 6.7 Hz, 3H), 1.71 – 1.54 (m, 6H), 1.43 – 1.34 (m, 2H), 1.32 – 1.19 (m, 20H), 0.87 – 0.81 (m, 3H).

Synthesis of Tail 6-oxohexyl (9Z,12Z)-octadeca-9,12-dienoate

Follow the synthesis method above. ^{1}H NMR (400 MHz, $CDCl_3$) δ 8.70 (s, 1H), 5.52 – 5.12 (m, 4H), 4.05 (t, *J* = 6.7 Hz, 2H), 3.12 – 2.98 (m, 4H), 2.40 – 2.26 (m, 2H), 2.02 (q, *J* = 6.9 Hz, 4H), 1.64 – 1.50 (m, 6H), 1.50 – 1.20 (m, 16H), 0.90 – 0.82 (m, 3H).

General Synthesis Route of Tail B

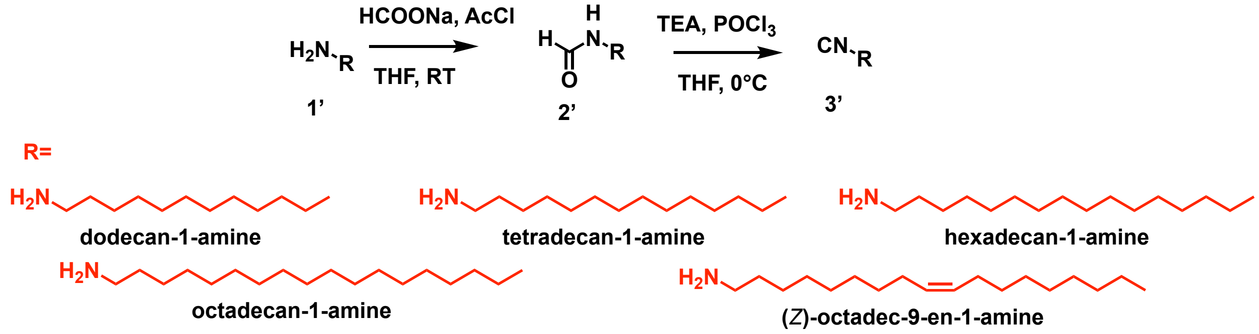

Tetrahydrofuran (82.75 ml) and solid sodium formate (2.2511 g) were added into a 250 mL round-bottomed flask and stirred to form a suspension. A DCM (34.755 mL) solution containing acetyl chloride (1.0 M) was taken in a constant pressure titration funnel and added dropwise to the suspension prepared above. The mixture was stirred at room temperature for 12 h after the dropwise addition to obtain reaction solution 1. 1' was dissolved in THF and added dropwise to reaction solution 1. The mixture was stirred at room temperature for 4 h after the dropwise addition to obtain reaction solution 2. Reaction solution 2 was diluted with 125 ml of deionized water, and the aqueous solution was extracted twice with 240 ml of ethyl acetate, each with an amount of 120 ml. The resulting extract was dried with anhydrous magnesium sulphate for 0.5 h and spin dried to obtain the crude 2'.

The crude 2' product and THF (50 mL) solution were added to a round-bottomed flask (100 mL) and placed in an ice bath at 0°C. The mixture was stirred for 10 min and TEA was added to the solution. The mixture was stirred in the ice bath for 15 min to obtain the reaction solution 1. THF (12.9 mL) and phosphorus oxychloride (3.519 g) were taken to prepare solution 1. Under ice bath conditions, solution 1 was added slowly dropwise to reaction solution 1 with a constant pressure-dropping funnel. The reaction was stirred for 1.5 h. 65 mL of a saturated sodium bicarbonate solution was added to dilute and wash the mixture, and the washed solution was extracted twice with 130 mL of ether, 65 mL each time. The extracts were combined, and anhydrous magnesium sulphate was added to the organic phase, stirred and dried for 0.5 h. The solution was removed by rotary evaporation. The crude product was purified by column using a Hexane/EA system.

Synthesis of Tail 1-isocyanododecane

Follow the synthesis method above. ^{1}H NMR (400 MHz, $CDCl_3$) δ 3.49 – 3.22 (m, 2H), 1.72 – 1.57 (m, 2H), 1.50 – 1.34 (m, 2H), 1.26 (d, *J* = 10.0 Hz, 16H), 0.89 – 0.84 (m, 3H).

Synthesis of Tail 1-isocyanotetradecane

Follow the synthesis method above. ^{1}H NMR (400 MHz, $CDCl_3$) δ 3.47 – 3.24 (m, 2H), 1.66 (ddt, *J* = 10.8, 8.4, 4.2 Hz, 2H), 1.46 – 1.37 (m, 2H), 1.27 (d, *J* = 11.2 Hz, 20H), 0.94 – 0.74 (m, 3H).

Synthesis of Tail 1-isocyanohexadecane

Follow the synthesis method above. ^{1}H NMR (400 MHz, $CDCl_3$) δ 3.45 – 3.26 (m, 2H), 1.66 (ddtt, *J* = 11.4, 6.7, 4.6, 2.3 Hz, 2H), 1.46 – 1.38 (m, 2H), 1.26 (d, *J* = 11.1 Hz, 24H), 0.87 (t, *J* = 6.8 Hz, 3H).

Synthesis of Tail 1-isocyanooctadecane

Follow the synthesis method above. ^{1}H NMR (400 MHz, $CDCl_3$) δ 3.48 – 3.23 (m, 2H), 1.73 – 1.61 (m, 2H), 1.46 – 1.37 (m, 2H), 1.25 (s, 28H), 0.91 – 0.84 (m, 3H).

Synthesis of Tail (Z)-1-isocyanooctadec-9-ene

Follow the synthesis method above. ^{1}H NMR (400 MHz, $CDCl_3$) δ 5.41 – 5.21 (m, 2H), 3.45 – 3.29 (m, 2H), 1.99 (dq, *J* = 14.2, 6.0 Hz, 3H), 1.66 (ddd, *J* = 8.4, 5.6, 3.4 Hz, 2H), 1.48 – 1.35 (m, 2H), 1.28 (dt, *J* = 17.3, 6.2 Hz, 20H), 0.92 – 0.84 (m, 3H).

Top-performing lipid in high throughput screening

Synthesis method of top 1 lipid in high throughput screen ((Z)-7-((1H-pyrazol-4-yl)amino)-8-(octadec-9-en-1-ylamino)-8-oxooctyl nonanoate).

The synthesis of the top-performing lipid in 1,200 screening through 3CR-Ugi reaction. A mixture of piperidin-1-amine (50 mg, 601.73 µmol), 6-oxobutyl octanoate (160.96 mg, 601.73 µmol) and (Z)-1-isocyanooctadec-9-ene (154.27 mg, 601.73 µmol) in anhydrous solvent DCM (240 µL) and MeOH (160 µL) and was stirred in capped glass vials at room temperature overnight. The mixture was purified by flash column chromatography (0.1% ammonia hydroxide with 0-10% MeOH in DCM) to obtain finial compound as colorless oil. (285.0 mg, 451.66 µmol, 75.06%). ^{1}H NMR (400 MHz, $CDCl_3$) δ 7.32 – 7.04 (m, 2H), 6.92 (t, *J* = 4.9 Hz, 1H), 5.33 (p, *J* = 3.6 Hz, 2H), 4.10 (tt, *J* = 5.6, 4.1 Hz, 3H), 3.25 (td, *J* = 5.9, 4.8 Hz, 2H), 2.39 – 2.24 (m, 3H), 2.03 – 1.90 (m, 4H), 1.68 – 1.53 (m, 3H), 1.48 – 1.20 (m, 41H), 0.94 – 0.81 (m, 6H).

Tail Synthesis for AGILE-identified structure

Synthesis of olealdehyde

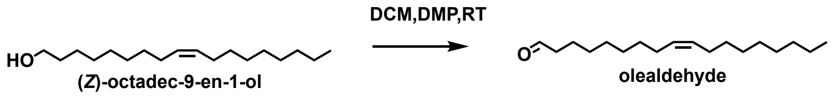

Olealdehyde was made using the same procedure as the synthesis method above. ^{1}H NMR (400 MHz, $CDCl_3$) δ 9.75 (t, *J* = 1.9 Hz, 1H), 5.34 (qd, *J* = 3.8, 1.7 Hz, 2H), 2.41 (td, *J* = 7.4, 1.9 Hz, 2H), 2.00 (q, *J* = 6.1 Hz, 4H), 1.62 (p, *J* = 7.3 Hz, 2H), 1.34 – 1.22 (m, 20H), 0.91 – 0.83 (m, 3H).

Synthesis of palmitaldehyde

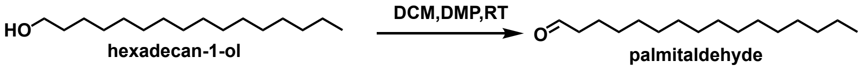

Palmitaldehyde was made using the same procedure as the synthesis method above. ^{1}H NMR (400 MHz, $CDCl_3$) δ 9.76 (s, 1H), 2.41 (td, J = 7.4, 1.9 Hz, 2H), 1.62 (dd, J = 9.3, 5.2 Hz, 2H), 1.25 (s, 23H), 0.90 – 0.84 (m, 3H).

Synthesis of 4-oxobutyl 4-methylnonanoate

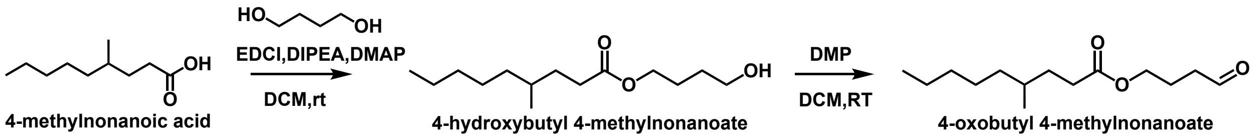

4-hydroxybutyl 4-methylnonanoate was made using the same procedure as the synthesis method above. ^{1}H NMR (400 MHz, $CDCl_3$) δ 4.09 – 4.04 (m, 2H), 3.64 (t, J = 6.3 Hz, 2H), 2.27 (td, J = 9.3, 6.1 Hz, 2H), 1.70 – 1.57 (m, 4H), 1.45 – 1.15 (m, 10H), 0.90 – 0.77 (m, 6H). 4-oxobutyl 4-methylnonanoate was made using the same procedure as the synthesis method above. ^{1}H NMR (400 MHz, $CDCl_3$) δ 9.78 (s, 1H), 4.08 (t, J = 6.3 Hz, 2H), 2.53 (td, J = 7.2, 1.3 Hz, 2H), 2.28 (td, J = 9.4, 6.1 Hz, 2H), 2.01 – 1.90 (m, 2H), 1.66 – 1.57 (m, 1H), 1.44 – 1.19 (m, 10H), 0.88 – 0.83 (m, 6H).

Synthesis of 4-oxobutyl (E)-dec-2-enoate

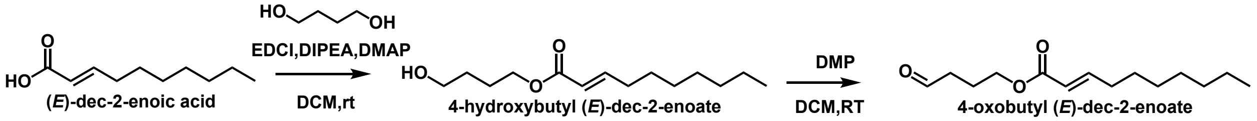

4-hydroxybutyl (*E*)-dec-2-enoate was made using the same procedure as the synthesis method above. ^{1}H NMR (400 MHz, $CDCl_3$) δ 6.89 (dt, J = 15.6, 6.9 Hz, 1H), 5.73 (dt, J = 15.7, 1.6 Hz, 1H), 4.08 (t, J = 6.5 Hz, 2H), 3.58 (t, J = 6.4 Hz, 2H), 2.12 (qd, J = 7.1, 1.6 Hz, 2H), 1.71 – 1.54 (m, 5H), 1.41 – 1.34 (m, 2H), 1.24 – 1.19 (m, 8H), 0.80 (d, J = 7.1 Hz, 3H).

4-oxobutyl (*E*)-dec-2-enoate was made using the same procedure as the synthesis method above. ^{1}H NMR (400 MHz, $CDCl_3$) δ 9.78 (t, J = 1.3 Hz, 1H), 6.95 (dt, J = 15.6, 6.9 Hz, 1H), 5.78 (d, J = 15.7 Hz, 1H), 4.15 (t, J = 6.3 Hz, 2H), 2.55 (td, J = 7.2, 1.3 Hz, 2H), 2.18 (qd, J = 7.1, 1.6 Hz, 2H), 1.99 (ddd, J = 13.6, 7.3, 6.3 Hz, 2H), 1.46 – 1.38 (m, 2H), 1.29 – 1.23 (m, 8H), 0.88 – 0.84 (m, 3H).

Synthesis of 4-oxobutyl octanoate

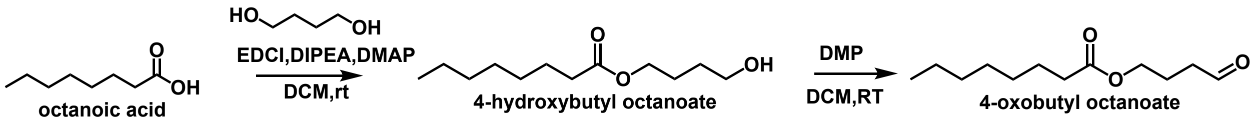

4-hydroxybutyl octanoate was made using the same procedure as the synthesis method above. ^{1}H NMR (400 MHz, $CDCl_3$) δ 4.06 (q, J = 7.1 Hz, 2H), 3.67 (p, J = 6.7 Hz, 1H), 3.08 (q, J = 7.4 Hz, 1H), 2.23 – 2.19 (m, 3H), 1.31 (d, J = 6.8 Hz, 7H), 1.22 – 1.20 (m, 9H), 0.82 (d, J = 4.0 Hz, 6H). 4-oxobutyl octanoate was made using the same procedure as the synthesis method above. ^{1}H NMR (400 MHz, $CDCl_3$) δ 9.77 (t, J = 1.3 Hz, 1H), 4.08 (t, J = 6.3 Hz, 2H), 2.53 (td, J = 7.2, 1.3 Hz, 2H), 2.30 – 2.22 (m, 2H), 1.96 (ddd, J = 13.5, 7.2, 6.3 Hz, 2H), 1.67 – 1.53 (m, 2H), 1.31 – 1.21 (m, 9H), 0.88 – 0.83 (m, 3H).

Synthesis of 4-oxobutyl oleate

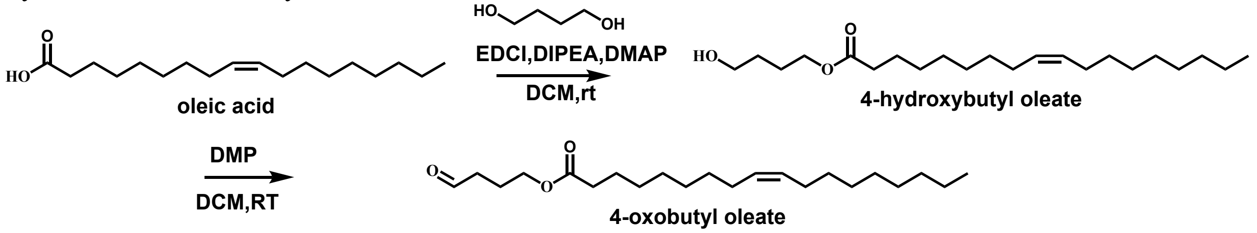

4-hydroxybutyl oleate was made using the same procedure as the synthesis method above. ^{1}H NMR (400 MHz, $CDCl_3$) δ 5.42 – 5.25 (m, 2H), 4.12 – 4.09 (m, 2H), 3.68 (t, J = 6.3 Hz, 2H), 2.29 (t, J = 7.6 Hz, 2H), 2.00 (q, J = 6.5 Hz, 4H), 1.76 – 1.60 (m, 6H), 1.31 – 1.24 (m, 20H), 0.87 (t, J = 6.7 Hz, 3H). 4-oxobutyl oleate was made using the same procedure as the synthesis method above. ^{1}H NMR (400 MHz, $CDCl_3$) δ 9.70 (t, J = 1.3 Hz, 1H), 5.45 – 5.15 (m, 2H), 4.14 (m, 2H), 2.52 – 2.18 (m, 4H), 2.12 – 1.78 (m, 6H), 1.70 – 1.50 (m, 2H), 1.41 – 1.11 (m, 20H), 1.02 – 0.78 (m, 3H).

Synthesis of 1-isocyanoundecane

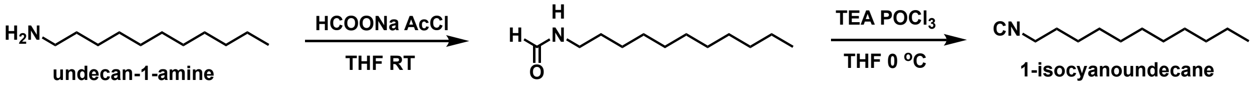

1-isocyanoundecane was made using the same procedure as the synthesis method above. ^{1}H NMR (400 MHz, $CDCl_3$) δ 3.42 – 3.27 (m, 2H), 1.63 – 1.52 (m, 2H), 1.31 – 1.24 (m, 16H), 0.88 – 0.86 (m, 3H).

Synthesis of 1-isocyanoheptadecane

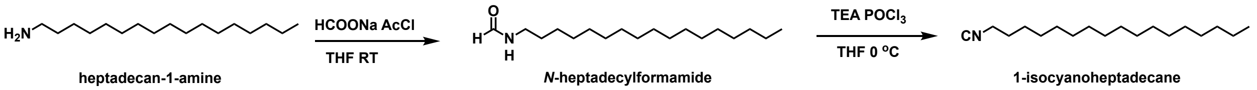

1-isocyanoheptadecane was made using the same procedure as the synthesis method above. ^{1}H NMR (400 MHz, $CDCl_3$) δ 3.44 – 3.29 (m, 2H), 1.74 – 1.37 (m, 4H), 1.25 (d, J = 1.5 Hz, 15H), 0.87 (td, J = 6.9, 1.8 Hz, 3H).

The mechanism of the Ugi-3CR reaction is mediated by isocyanide and its Markush structure.

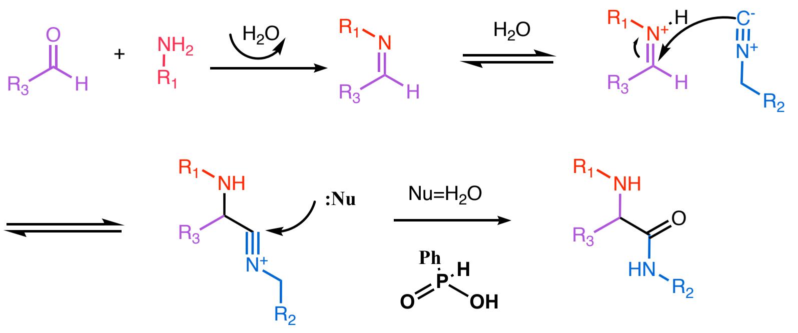

Top-performing lipid synthesis

Synthesis Method of Lipid **H9** (*Z*)-5-(octadec-9-en-1-ylamino)-5-oxo-4-(piperidin-1-ylamino)pentyl octanoate.

The synthesis of **H9** through 3CR-Ugi reaction. A mixture of piperidin-1-amine (25 mg, 249.59 µmol), 4-oxobutyl octanoate (53.49 mg, 249.59 µmol) and (Z)-1-isocyanooctadec-9-ene (69.26 mg, 249.59 µmol) in anhydrous solvent DCM (240 µL) and MeOH (160 µL) with the catalyst phenylphosphinic acid (7.09 mg, 49.92 µmol) and was stirred in capped glass vials at room temperature overnight. The mixture was purified by flash column chromatography (0.1% ammonia hydroxide with 0-10% MeOH in DCM) to obtain compound **H9** as colorless oil. (35.0 mg, 59.13 µmol, 23.69%). ^{1}H NMR (400 MHz, $CDCl_3$) δ 7.03 (t, *J* = 5.8 Hz, 1H), 5.46 – 5.31 (m, 2H), 4.05 (t, *J* = 6.1 Hz, 2H), 3.40 – 3.26 (m, 2H), 3.23 – 3.11 (m, 1H), 2.69 (s, 2H), 2.41 (s, 2H), 2.28 (t, *J* = 7.6 Hz, 2H), 1.99 (dd, *J* = 14.1, 7.7 Hz, 4H), 1.74 – 1.46 (m, 14H), 1.33 – 1.21 (m, 31H), 0.91 – 0.83 (m, 6H). MS: m/z calculated for $C_{36}H_{69}N_3O_3^+$ (M+H), 592.53; observed (ESI ms) m/z 592.53.

Synthesis Method of Lipid **R6** 2-((3-(diethylamino)propyl) amino) -N-undecylheptadecanamide.

The synthesis of **R6** through 3CR-Ugi reaction. A mixture of *N*, *N*-diethylpropane-1,3-diamine (25 mg, 191.96 µmol), palmitaldehyde (46.15 mg, 191.96 µmol) and 1-isocyanoundecane (34.81 mg, 191.96 µmol) in anhydrous solvent DCM (240 µL) and MeOH (160 µL) with the catalyst phenylphosphinic acid (5.46 mg, 39.39 µmol) and was stirred in capped glass vials at room temperature overnight. The mixture was purified by flash column chromatography (0.1% ammonia hydroxide with 0-10% MeOH in DCM) to obtain compound **R6** as colorless oil. (39.8 mg, 72.1 µmol, 37.56%). ^{1}H NMR (400 MHz, $CDCl_3$) δ 3.27 – 3.18 (m, 2H), 2.99 (dd, *J* = 7.7, 4.7 Hz, 1H), 2.67 – 2.40 (m, 8H), 1.73 – 1.43 (m, 6H), 1.33 – 1.19 (m, 44H), 1.03 (td, *J* = 7.1, 3.1 Hz, 7H), 0.91 – 0.84 (m, 6H). MS: m/z calculated for $C_{35}H_{73}N_3O^+$ (M+H), 552.58; observed (ESI ms) m/z 552.73.

Supplementary Note 2.

The Mass Spectra for H1-H15 and H31-H45.

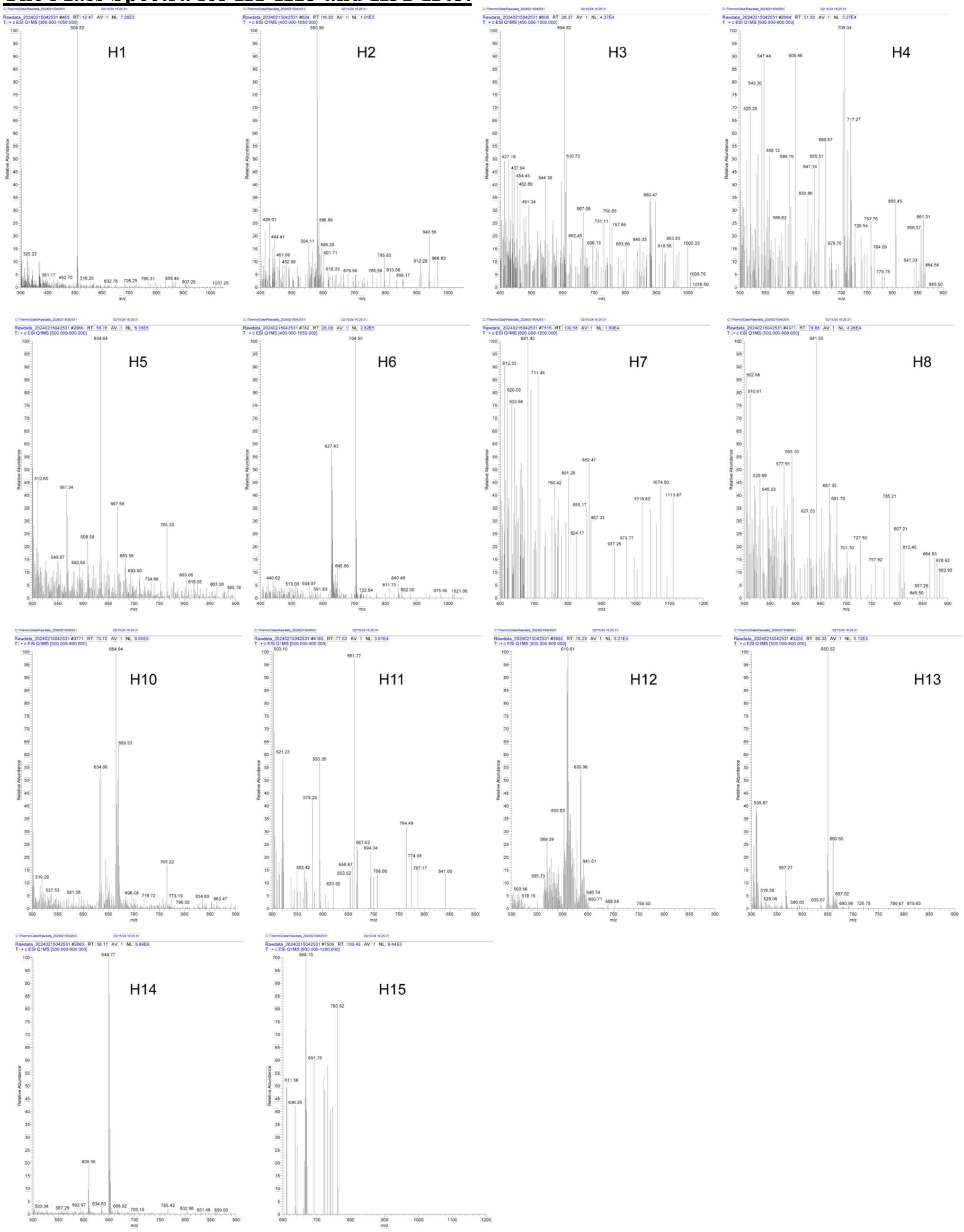

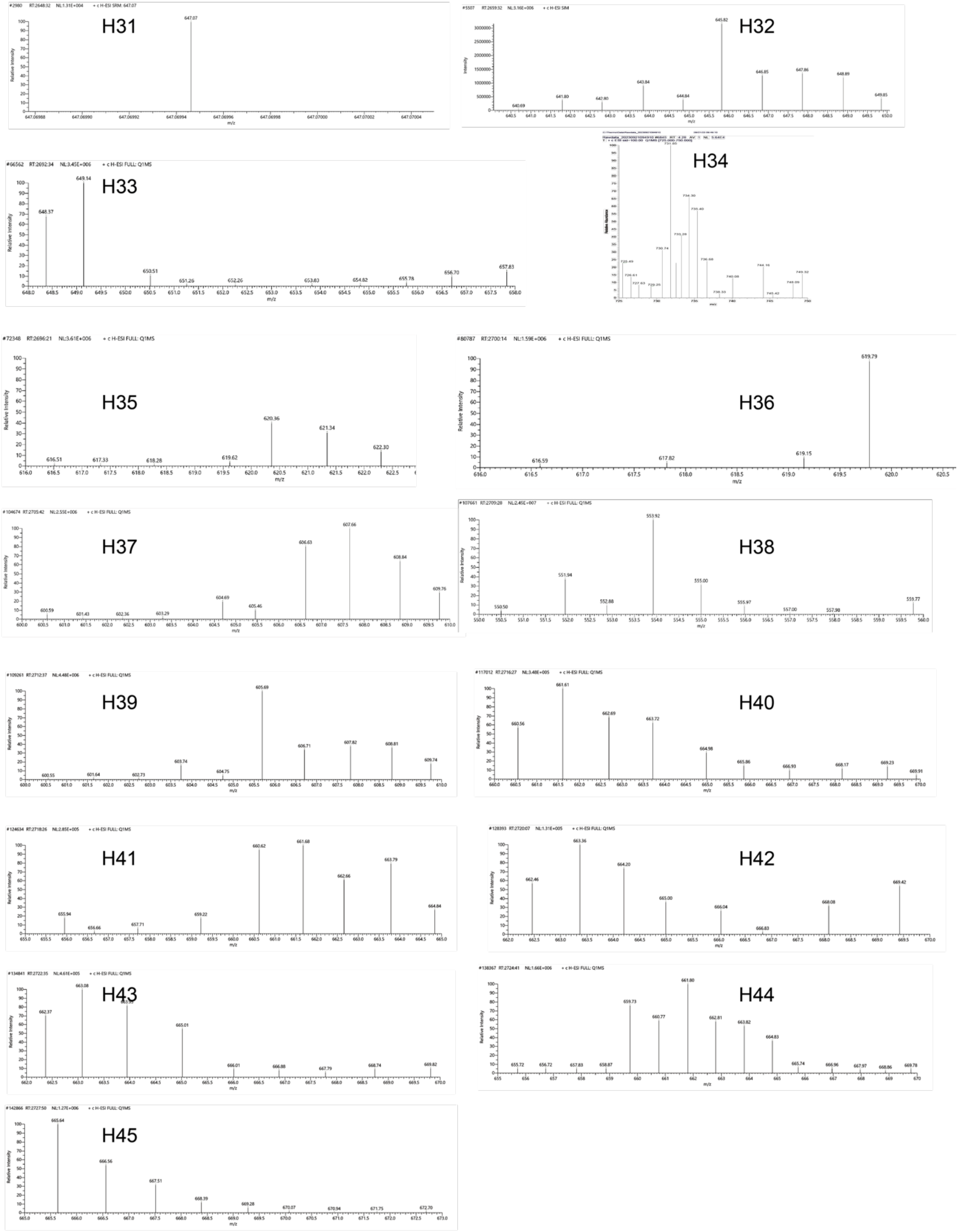
H31
H32
H33
H34
H35
H36
H37
H38
H39
H40
H41
H42
H43
H44
H45

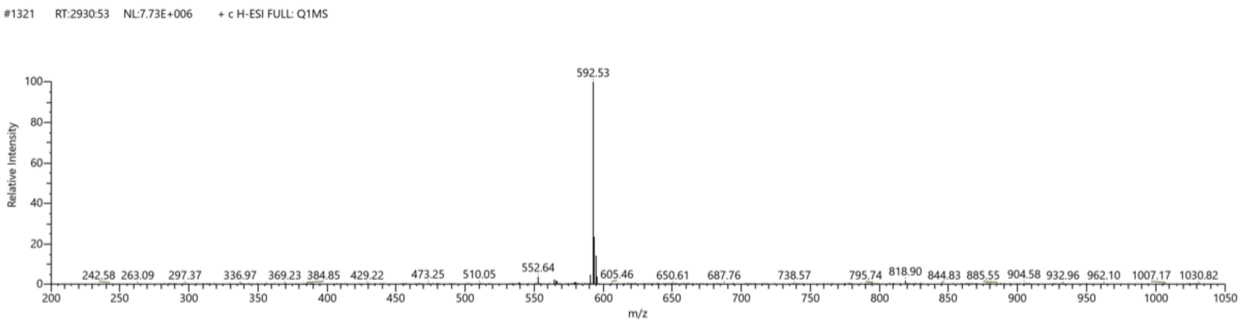

Mass Spectrum of **H9**.

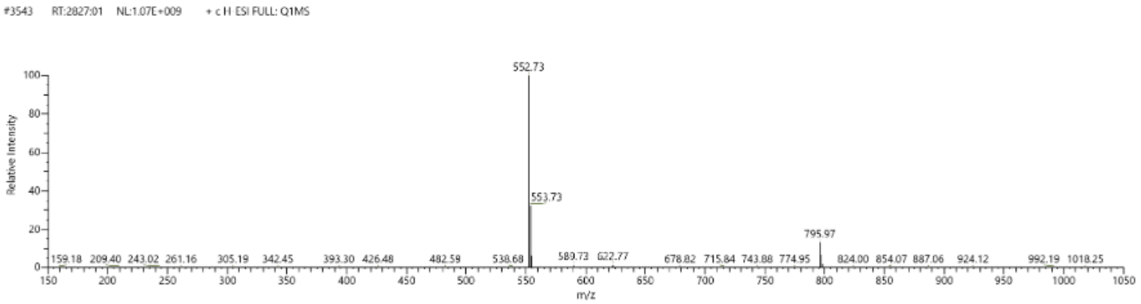

Mass Spectrum of **R6**.

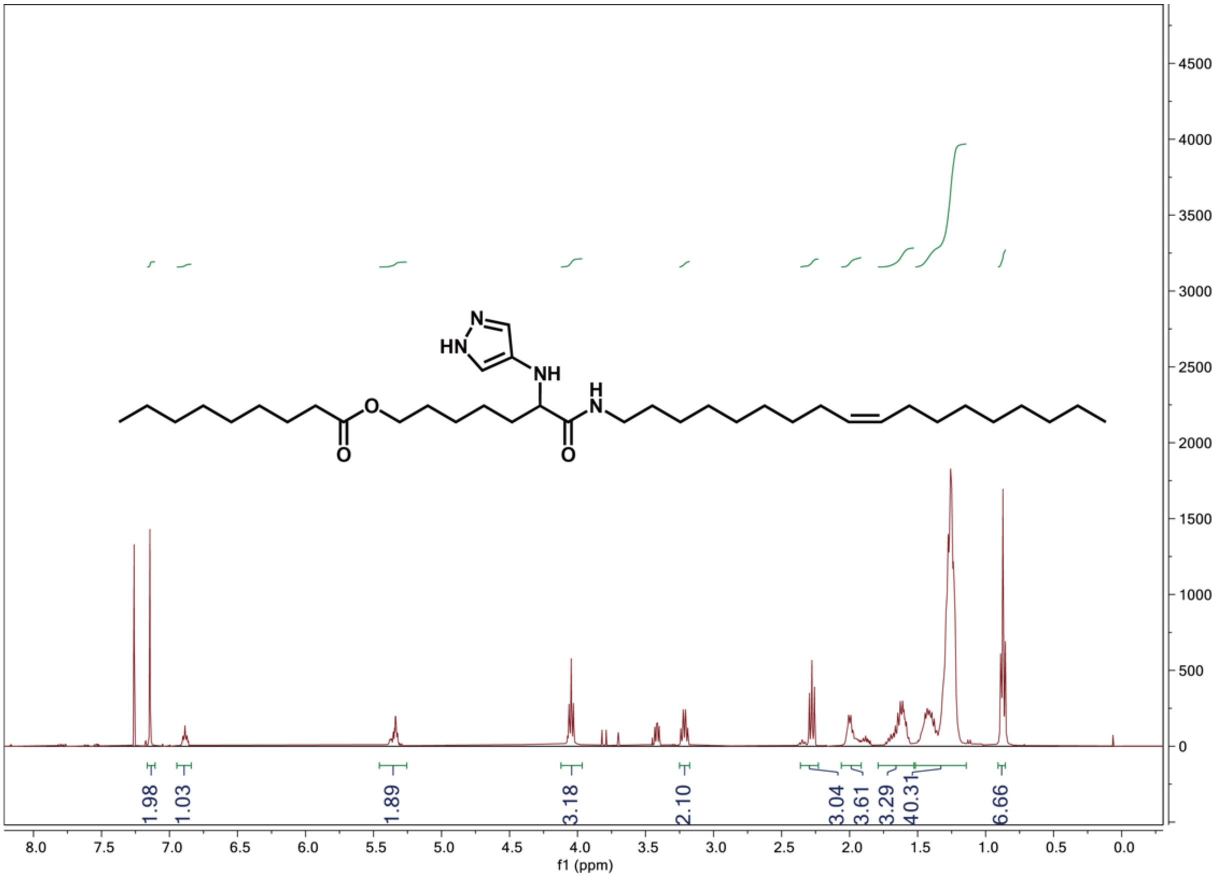

^{1}H NMR of **Top 1** in 1200.

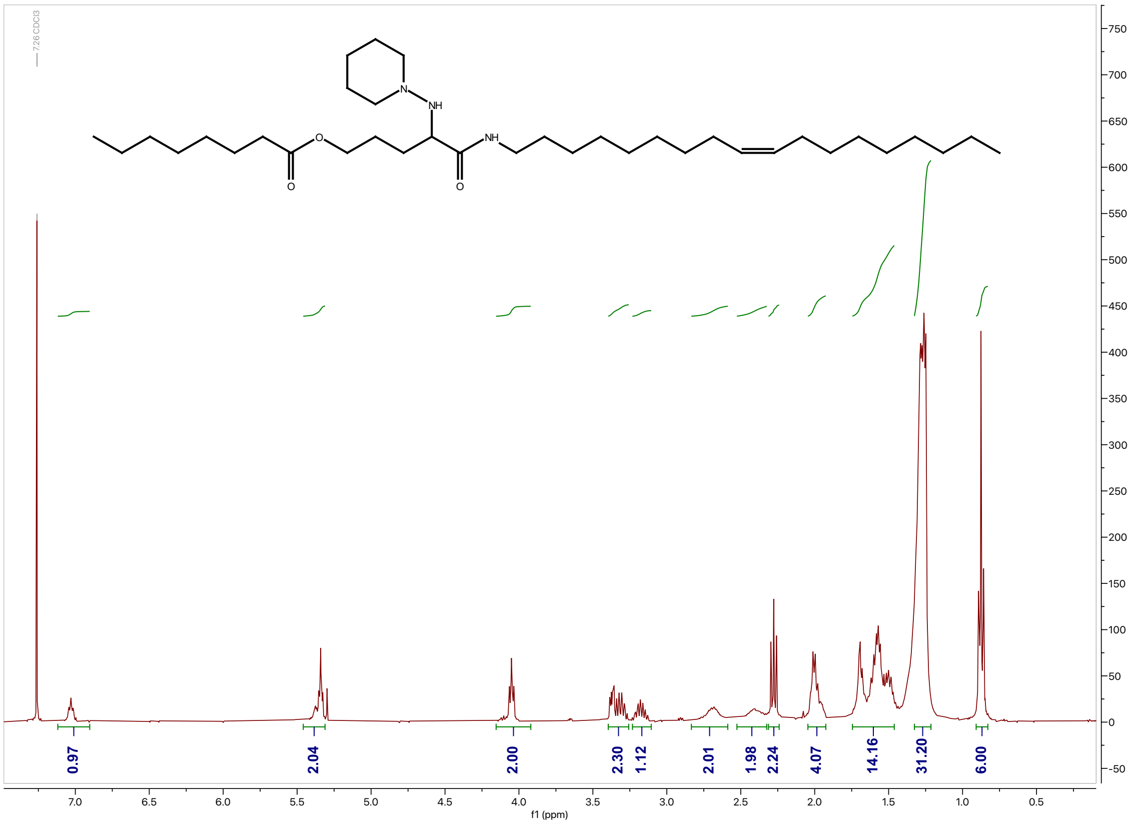

^{1}H NMR of **H9**.

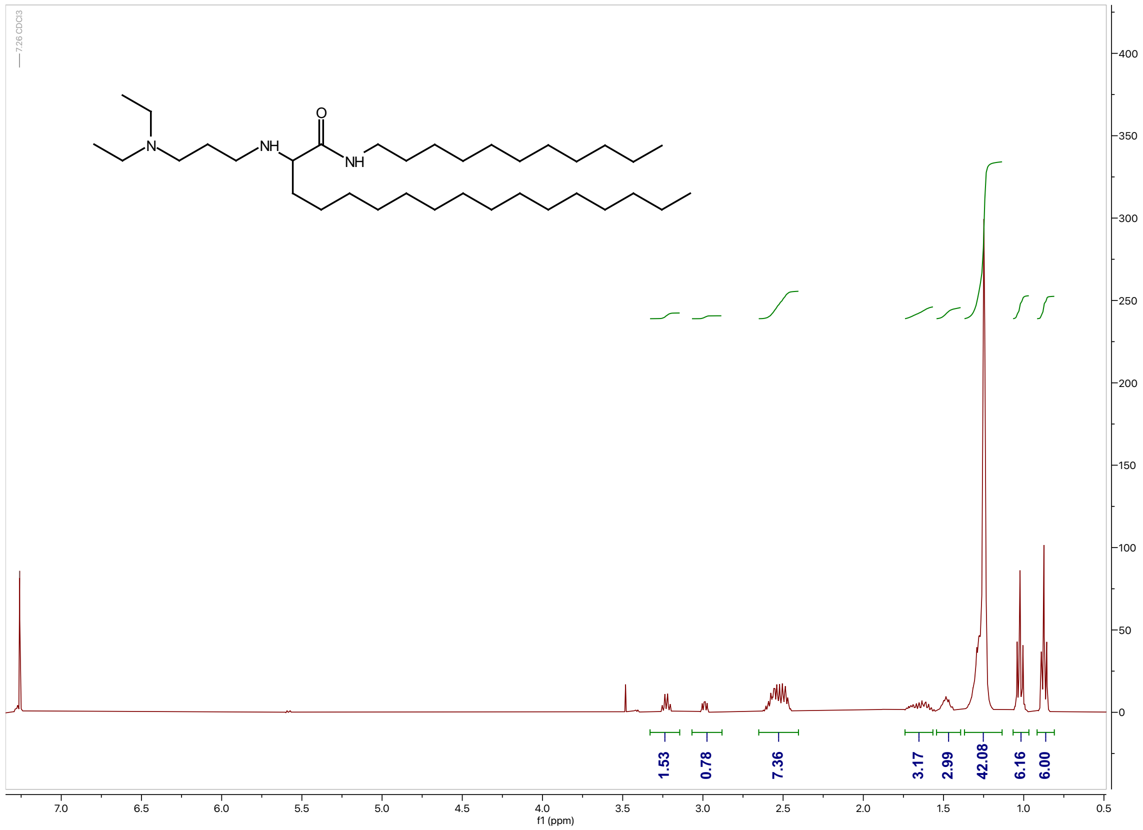
7.26 CDCl3
N
NH
O
NH
1.53
0.78
7.36
3.17
2.99
42.08
6.16
6.00
7.0
6.5
6.0
5.5
5.0
4.5
4.0
3.5
3.0
2.5
2.0
1.5
1.0
0.5
f1 (ppm)
400
350
300
250
200
150
100
50
0

^{1}H NMR of **R6**.

Supplementary Table 1
The structure and characterization of H1-H15 and H31-H45 lipids.

Compound	Chemical Formula	Structure	Calcd. (m/z)	Observed (See Suppl. Notes)
H1	$C_{32}H_{65}N_3O$	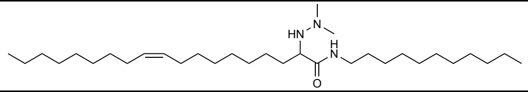	508.51	508.52
H2	$C_{35}H_{69}N_3O_3$	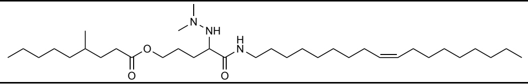	580.53	580.56
H3	$C_{39}H_{77}N_3O$	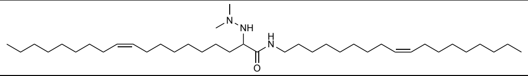	604.61	604.82
H4	$C_{44}H_{87}N_3O_3$	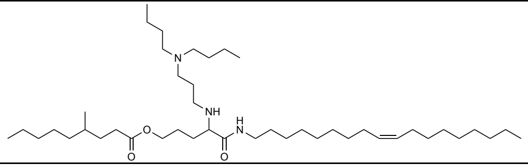	706.67	706.04
H5	$C_{41}H_{83}N_3O$	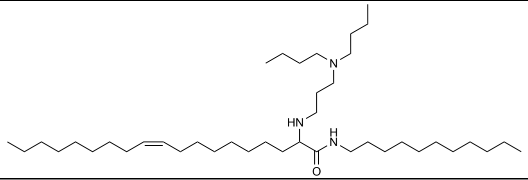	634.65	634.64
H6	$C_{44}H_{85}N_3O_3$	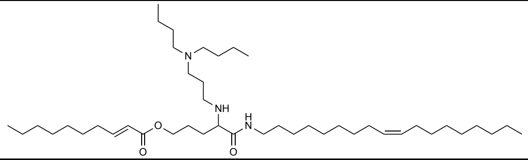	704.66	704.55
H7	$C_{38}H_{73}N_3O_3$	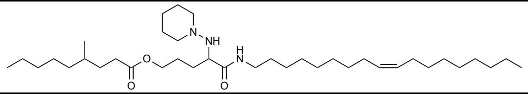	620.57	620.03
H8	$C_{38}H_{71}N_3O_3$	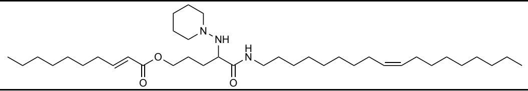	641.55	641.55
H9	$C_{36}H_{69}N_3O_3$	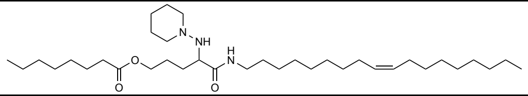	592.53	592.53
H10	$C_{41}H_{81}N_3O_3$	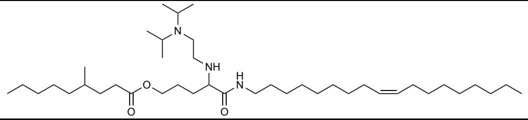	664.63	664.94
H11	$C_{41}H_{80}N_3O_3$	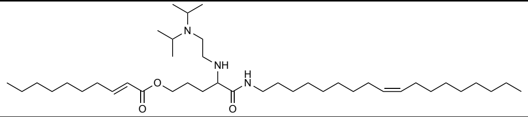	663.61	663.29
H12	$C_{37}H_{75}N_3O_3$	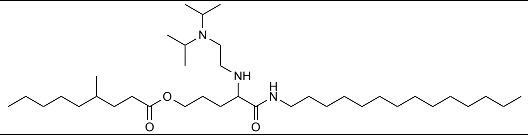	610.58	610.61
H13	$C_{40}H_{79}N_3O_3$	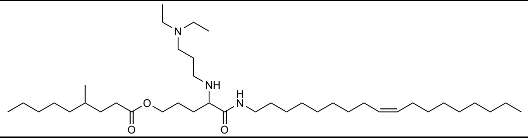	650.61	650.52

H14	$C_{40}H_{77}N_3O_3$	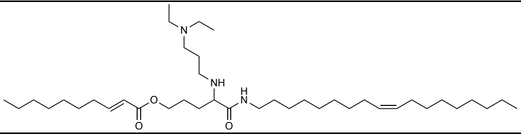	648.60	648.85
H15	$C_{48}H_{93}N_3O_3$	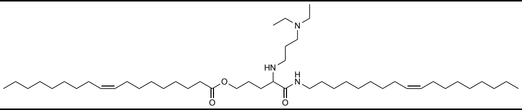	760.72	760.52
H31	$C_{40}H_{77}N_3O_3$	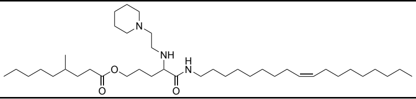	647.60	647.60
H32	$C_{40}H_{75}N_3O_3$	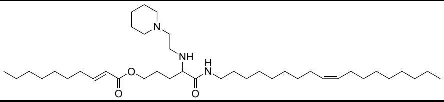	645.58	645.82
H33	$C_{40}H_{79}N_3O_3$	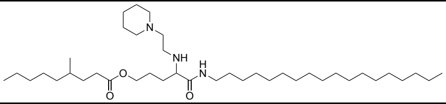	649.61	649.14
H34	$C_{46}H_{89}N_3O_3$	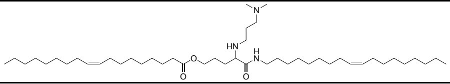	731.69	731.85
H35	$C_{38}H_{75}N_3O_3$	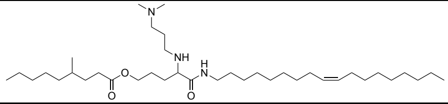	621.58	621.34
H36	$C_{38}H_{73}N_3O_3$	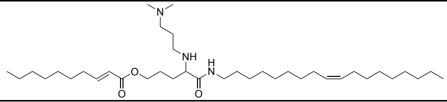	619.57	619.79
H37	$C_{37}H_{73}N_3O_3$	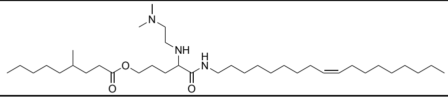	607.57	607.66
H38	$C_{33}H_{67}N_3O_3$	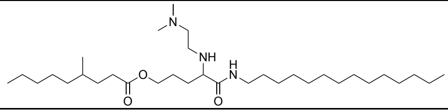	553.52	553.92
H39	$C_{37}H_{71}N_3O_3$	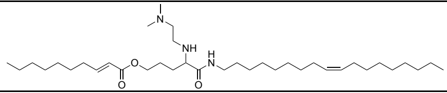	605.55	605.69
H40	$C_{41}H_{79}N_3O_3$	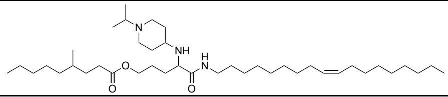	661.61	661.61
H41	$C_{41}H_{77}N_3O_3$	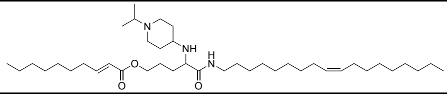	659.60	659.22
H42	$C_{41}H_{81}N_3O_3$	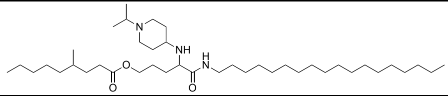	663.63	663.36
H43	$C_{40}H_{77}N_3O_4$	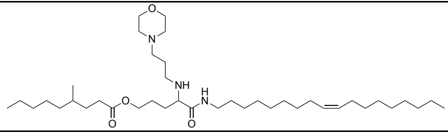	663.59	663.08
H44	$C_{40}H_{75}N_3O_4$	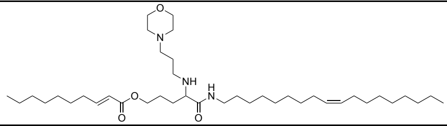	661.58	661.80

H45	$C_{40}H_{79}N_3O_4$	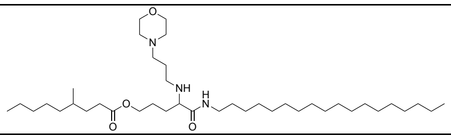	665.61	665.64

Supplementary Table 2
The characterization of H1-H15 and H31-H45 LNPs.

LNP	Size (nm)	PDI	*In vitro Transfection Potency* (HeLa, Log_2)	*In vivo Transfection Potency* (I.M.)
H1	134.6	0.14	7.81	3.625E+04
H2	138.0	0.14	6.32	3.494E+04
H3	108.7	0.09	7.01	1.941E+04
H4	110.0	0.17	7.63	4.065E+04
H5	122.6	0.14	9.95	1.384E+05
H6	119.9	0.15	9.61	3.299E+04
H7	134.8	0.01	5.43	7.301E+04
H8	132.9	0.09	4.09	1.892E+04
H9	123.8	0.08	12.48	2.773E+06
H10	127.5	0.15	6.51	3.379E+04
H11	151.0	0.16	7.77	5.005E+05
H12	147.5	0.17	10.15	4.675E+04
H13	135.5	0.02	9.59	1.870E+04
H14	128.2	0.12	7.62	1.952E+04
H15	117.5	0.14	5.58	6.916E+05
H31	134.8	0.1	6.30	8.059E+05
H32	132.9	0.12	6.21	5.577E+04
H33	133.8	0.1	5.87	4.46E+04
H34	137.8	0.15	1.46	6.802E+04
H35	135.5	0.1	3.10	5.662E+04
H36	139.0	0.1	3.54	1.109E+04
H37	132.8	0.13	6.21	7.084E+05
H38	133.9	0.16	6.93	3.388E+05
H39	137.9	0.16	4.01	2.286E+05
H40	135.4	0.23	0.73	1.844E+04
H41	134.6	0.14	0.75	3.195E+04
H42	138.0	0.14	0.86	2.989E+04
H43	121.6	0.14	5.87	2.472E+04
H44	119.9	0.15	-0.87	3.911E+04
H45	108.4	0.15	5.55	2.835E+05

Supplementary Table 3
Formulations and characterization of H9-containing LNPs.

Number	Factor					Response
	Lipid/mRNA weight ratio	H9	DOPE	PEG	Chol	HeLa (Log_2)
1	7.5	60	20	1.5	11	9.246
2	7.5	60	15	0.5	17	11.086
3	15	30	10	1.5	43.5	4.466
4	7.5	30	20	2.5	40	8.412
5	7.5	30	10	2.5	50	2.616
6	7.5	60	10	2.5	20	-0.071
7	15	60	10	2.5	12.5	8.257
*8	15	60	20	0.5	4.5	12.244
9	15	60	10	0.5	14.5	8.169
10	11.25	60	20	2.5	6.25	9.016
11	15	45	20	2.5	17.5	8.923
12	7.5	45	10	0.5	37	6.256
13	15	30	20	0.5	34.5	9.252
14	11.25	45	15	1.5	27.25	8.382
15	15	30	15	2.5	37.5	4.237
16	7.5	30	20	0.5	42	8.024
17	11.25	30	10	0.5	48.25	6.939

Note: The LNP formulation with the best performance for H9 was *8.

Supplementary Table 4. Formulations and characterization of R6-containing LNPs.

Number	Factor					Response
	Lipid/mRNA weight ratio	R6	DOTAP	PEG	Chol	Raw 264.7 (Log_2)
1	7.5	60	20	1.5	11	6.518
#2	7.5	60	15	0.5	17	6.680
3	15	30	10	1.5	43.5	4.327
4	7.5	30	20	2.5	40	4.414
5	7.5	30	10	2.5	50	2.705
6	7.5	60	10	2.5	20	2.825
7	15	60	10	2.5	12.5	3.102
8	15	60	20	0.5	4.5	5.172
9	15	60	10	0.5	14.5	5.574
10	11.25	60	20	2.5	6.25	5.115
11	15	45	20	2.5	17.5	4.621
12	7.5	45	10	0.5	37	6.123
13	15	30	20	0.5	34.5	5.604
14	11.25	45	15	1.5	27.25	3.333
15	15	30	15	2.5	37.5	3.628
16	7.5	30	20	0.5	42	6.443
17	11.25	30	10	0.5	48.25	5.150

Note: Formulation #2 was identified as the top performing LNP formulation for R6.

Supplementary Table 5. Key metrics for variance prediction and the Pearson correlation coefficient.

**Model**	**R^2**	**Pearson Correlation**
Ridge	-1.036	0.514
Lasso	-0.010	0.040
Gradient Boosting	0.090	0.308
SVM	0.067	0.409
**AGILE**	**0.249**	**0.573**

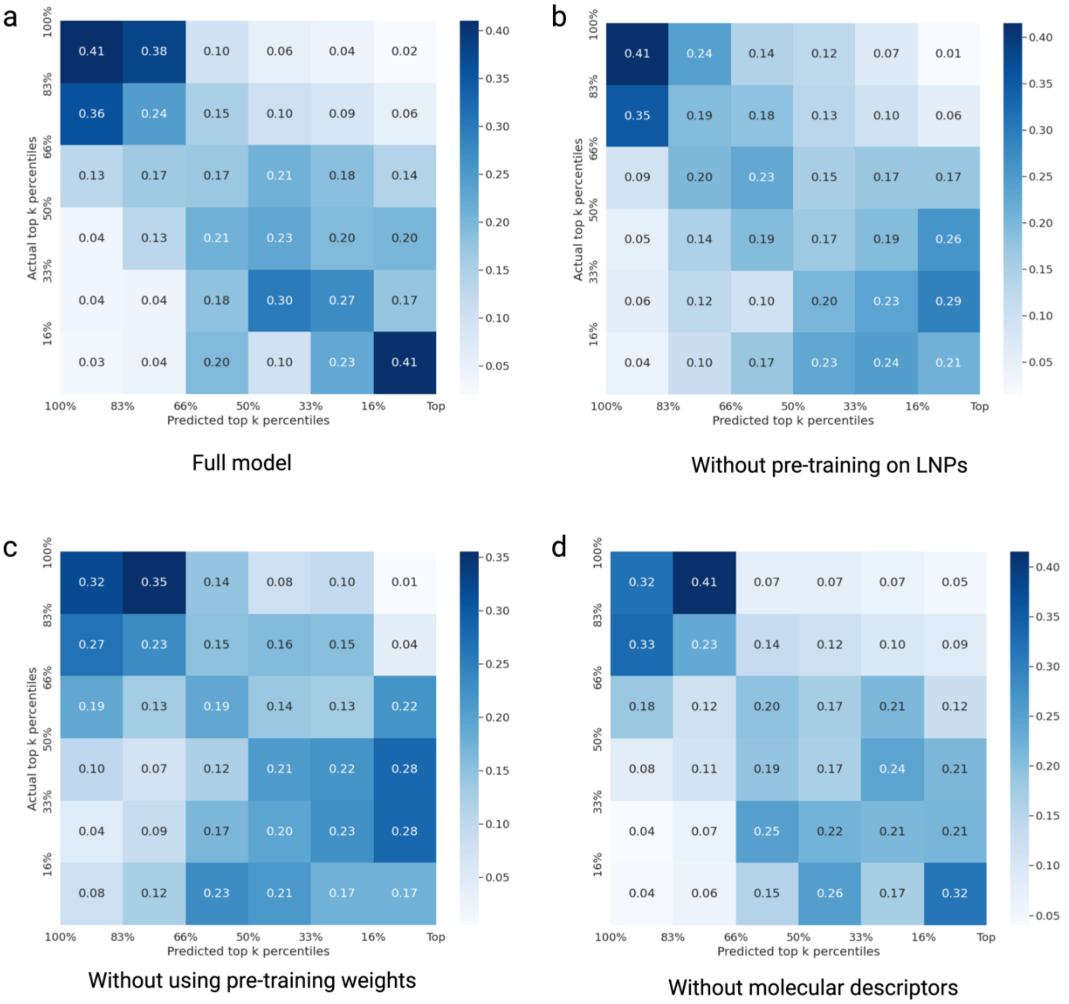

Supplementary Figure 1. This figure provides an ablation study of the AGILE pipeline design, assessing the effect of individual components on overall model performance. For each figure, the precision matrix computed on the experimental library of 1,200 lipids is shown. The transfection potency is divided into six equal percentiles, which are predicted and compared to the actual results. (a) The performance of the fully featured model is presented, which has been fine-tuned on the Hela experimental dataset. (b) The precision matrix depicts the performance of a model variant that does not include pre-training on lipids. Although this iteration demonstrates a comparable ability to identify lower-performing lipids, a notable decrease in its capacity to discern top-performing instances is observed. (c) The figure showcases the performance of the model when pre-training weights are excluded. In this case, the model's ability to identify top performers decreases further, along with a moderate reduction in its capability to identify lower-performing instances, underlining the significant contribution of pre-training weights to model performance. (d) Displayed is the model's performance when the molecular descriptor module is not included. In this scenario, a concurrent and somewhat significant decline in the model's ability to identify both top and bottom performers is observed suggesting the importance of this module in the overall performance of the AGILE pipeline.

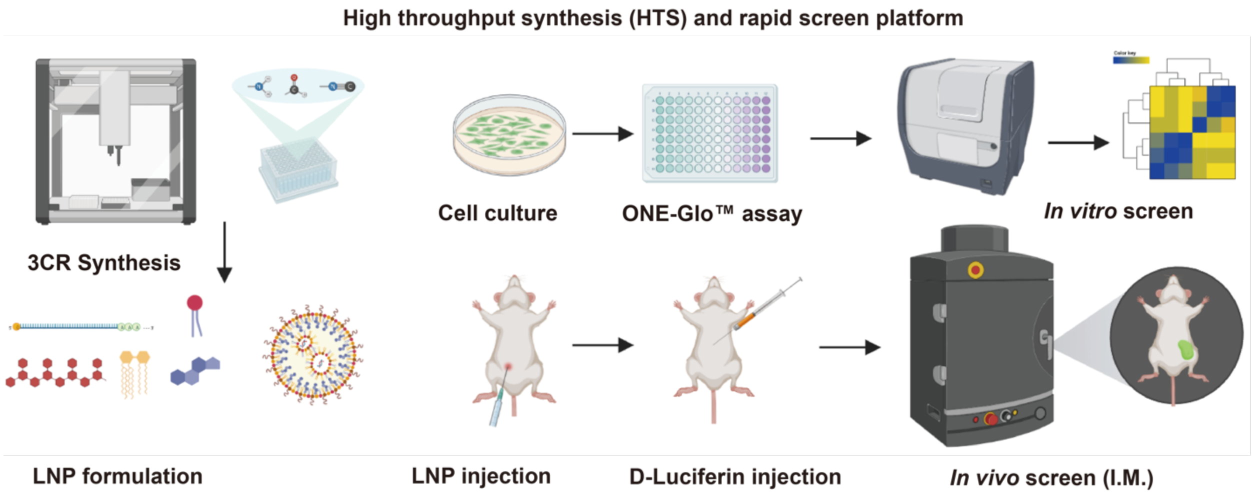

Supplementary Figure 2. Schematic diagram of the high-throughput synthesis and rapid screening platform. The figure was created with BioRender.com and released under a Creative Commons Attribution-NonCommercial-NoDerivs 4.0 International license.

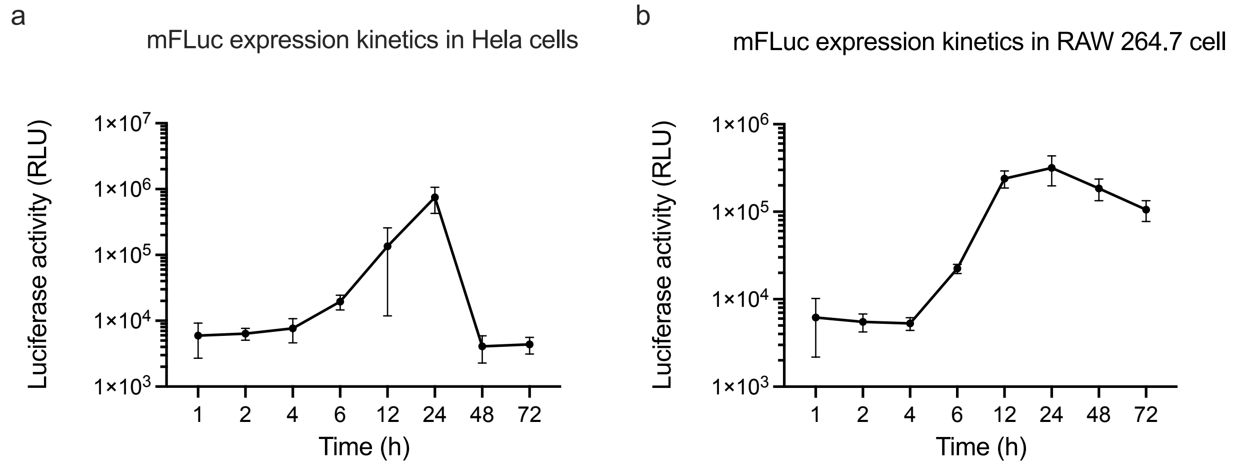

Supplementary Figure 3. Kinetic assays for time course analysis of (a) HeLa cells and (b) RAW264.7 cells. MC3 LNPs containing 0.1 μg of mFLuc, were added to pre-seeded HeLa cells in 96-well plates. The transfection of mFLuc was measured using the One-Glo Luciferase Assay System (Promega) (n =3).

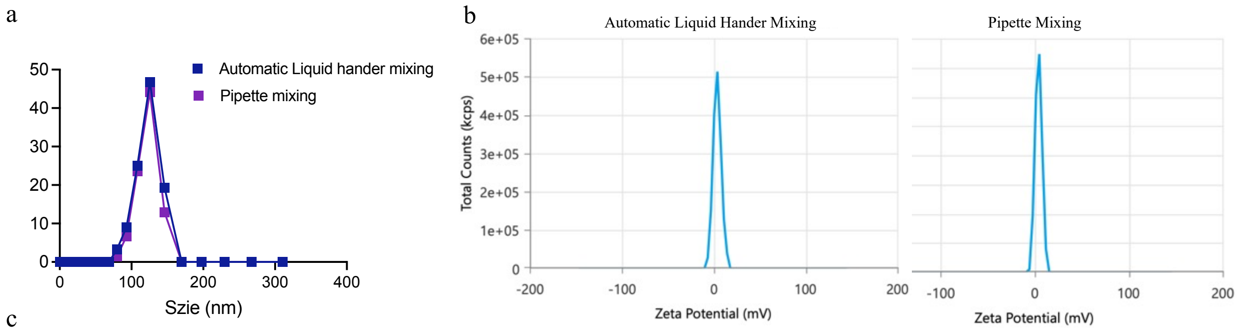

c

	Size (nm)	Zeta Potential (mV)	PDI	Encapsulation Efficiency (%)	In vivo (i.m.) Transfection	In vitro HeLa
**Automatic Liquid Handler Mixing**	125.6 ± 1.8	3.853 ± 0.6	0.19 ± 0.01	76.5 ± 7.55	4.1E+07 ± 1.2E+07	7.2E+05 ± 2.58E+05
**Pipette Mixing**	125.1 ± 2.3	3.411 ± 0.8	0.19 ± 0.01	79.4 ± 1.46	5.3E+07 ± 6.52E+06	7.3E+05 ± 9.54E+04

Supplementary Figure 4. Characterization of LNP prepared by different mixing ways. a. Size of MC3 LNPs formulated with Fluc mRNA measured by dynamic light scattering by different mixing ways. **b.** PDI of each MC3 LNP was prepared in different mixing ways. **c.** Characterization of MC3 LNPs, including size, zeta potential, PDI, and Encapsulation Efficiency. Quant-iT RiboGreen RNA assay (Invitrogen) was used to calculate mRNA encapsulation efficiency. The total flux of Luciferase signal (ROI) was measured at 6 hours following intramuscular administration of FLuc mRNA LNPs (0.125mg/kg, n = 3).

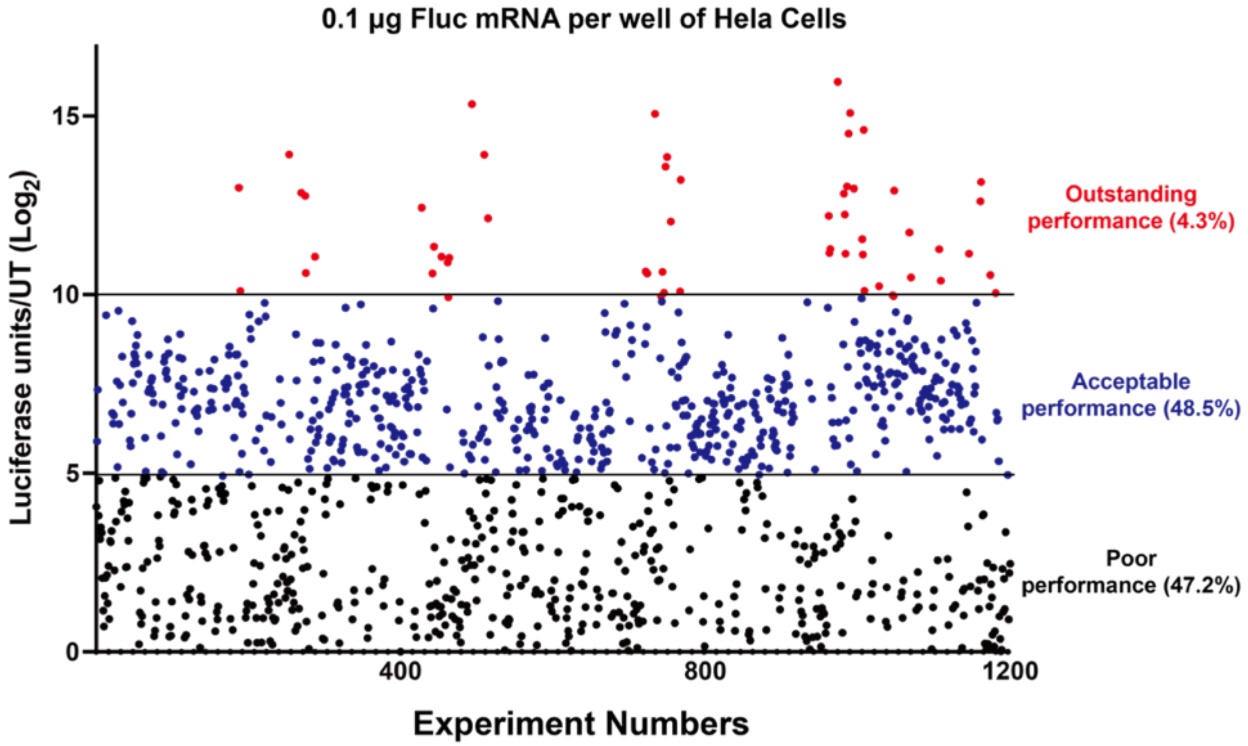

Supplementary Figure 5. Luciferase activity/untreated are shown as scatter plots for the 1,200 LNPs in Hela.

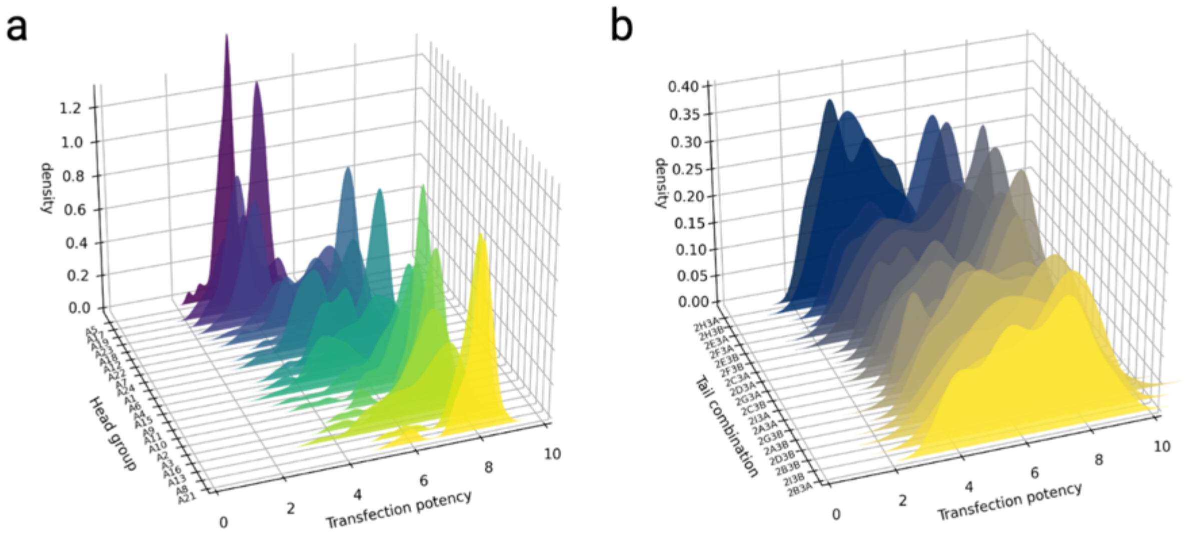

Supplementary Figure 6. The stratified distribution plots for predicted potencies across different categories. (a) Distribution plot demonstrating the potencies predicted for the Hela cell line, stratified according to head group classifications. (b) Similar stratified distribution plot for the Hela cell line, but the stratification is based on tail combinations.

Top 1-15 predictions ranked by raw prediction scores for the HeLa cells

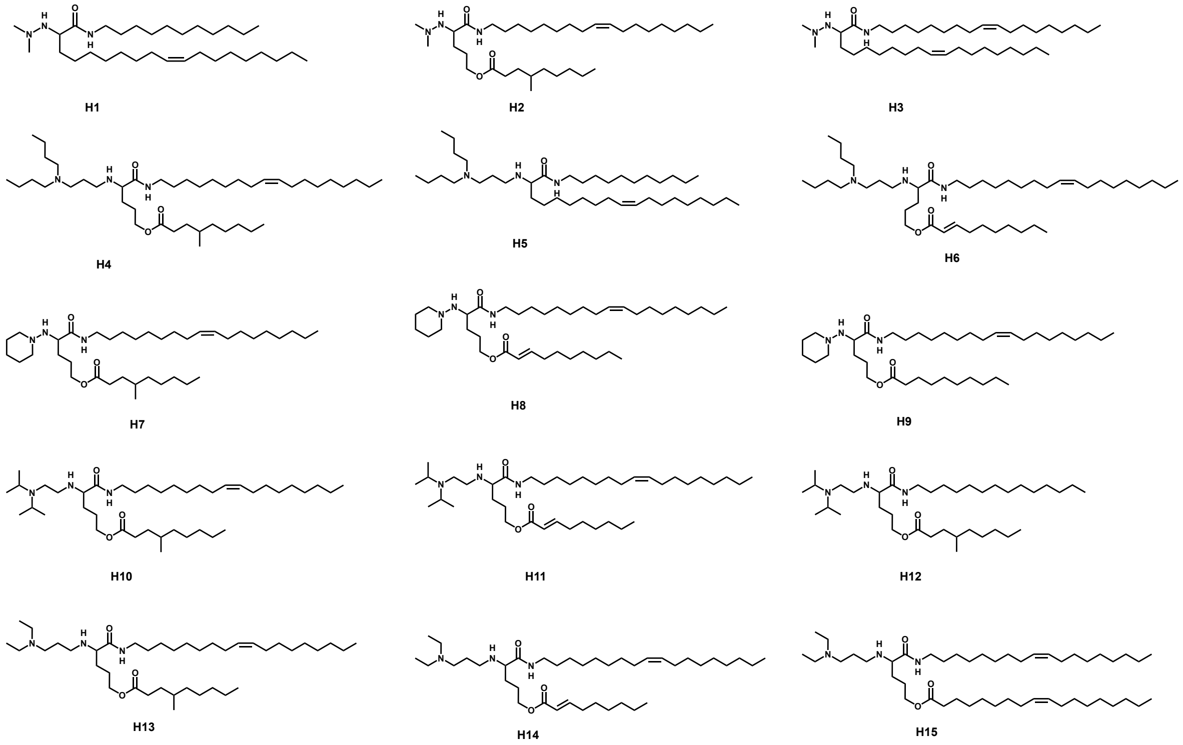

Supplementary Figure 7. Top 1-15 lipid candidate structures identified for Hela cells by the AGILE model.

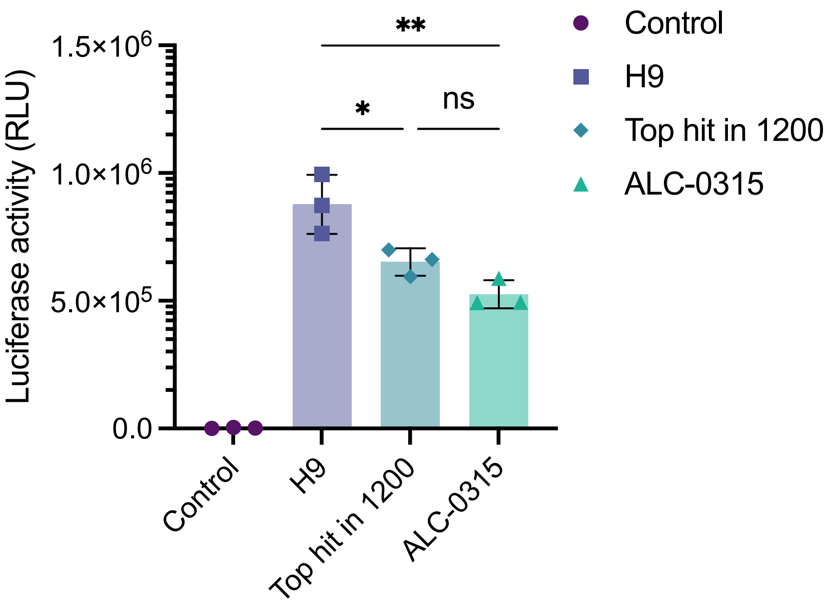

Supplementary Figure 8. Comparison of the mFluc transfection potency of the top hit in the initial 1,200 lipids pool with the H9 LNP in the HeLa cells (n = 3), control as untreated cells and ALC-0315 LNPs. LNPs formulated with cholesterol, DOPE, and DMG-PEG2000 at a molar ratio of 60/19.5/20/0.5 (n = 3). Statistical significance was analyzed by the two-tailed Student's t-test. *= p <0.05, **=p <0.01, ***=p <0.005. Data are presented as mean ± SD.

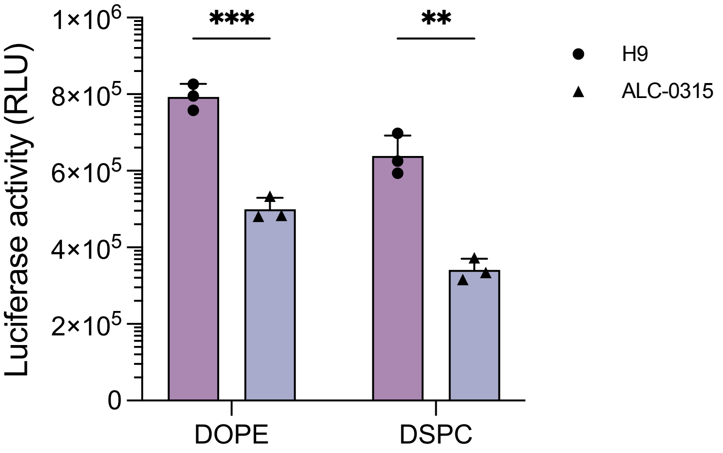

Supplementary Figure 9. Comparison of the mFluc transfection potency of the H9 and ALC-0315 LNPs with the same formulation (with different phospholipids DOPE or DSPC). LNPs formulated with cholesterol, DOPE or DSPC, and DMG-PEG2000 at a molar ratio of 60/19.5/20/0.5 (n = 3). Statistical significance was analyzed by the two-tailed Student's t-test. *= $p < 0.05$, **= $p < 0.01$, ***= $p < 0.005$. Data are presented as mean ± SD.

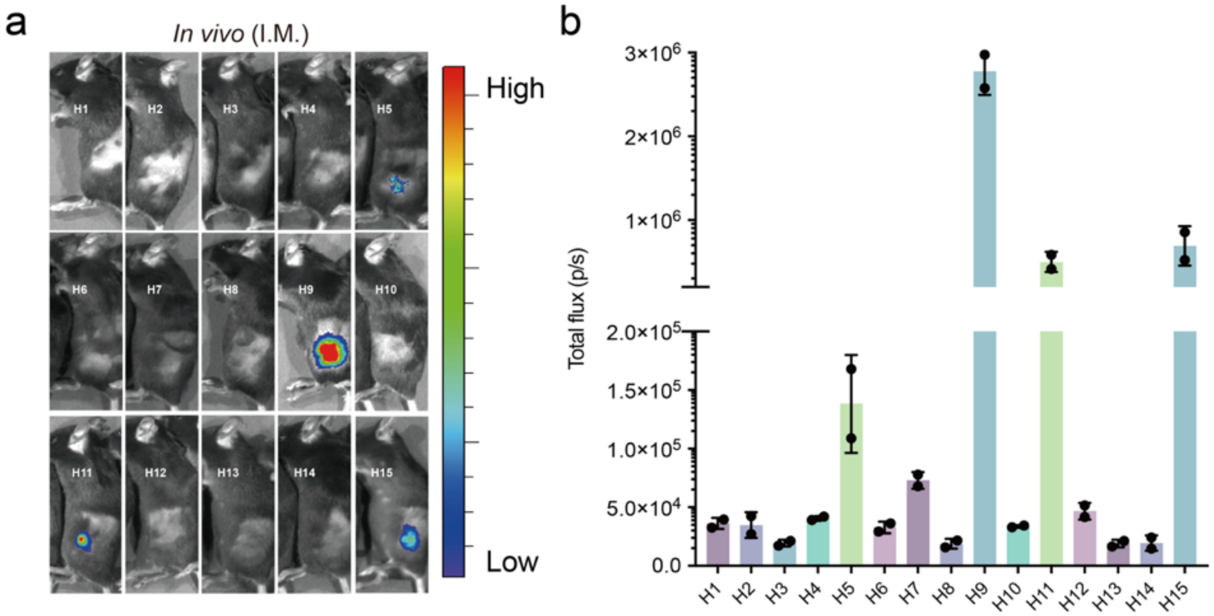

c

Pearson Correlations

		In vitro	In vivo
In vitro	Pearson Correlation	1	0.780**
	Sig. (2-tailed)		0.001
	N	15	15
In vivo	Pearson Correlation	0.780**	1
	Sig. (2-tailed)	0.001	
	N	15	15

Note. *p< 0.05, **p<0.01, ***p<0.001. Significant correlations are flagged using one (p < 0.05), two (p< 0.01) or three (p < .001) asterisks.

Supplementary Figure 10. a. Top 15 LNPs of unpurified ionizable lipids for intramuscular injection. LNPs formulated with Fluc encoding mRNA were injected intramuscularly into mice (0.2 mg mRNA/kg mouse, n = 2). The Fluc expression was visualized at 5 h. **b.** Histogram analysis of total flux in muscles. **c.** A Pearson correlation analysis was conducted by SPSS v27.0 to examine the relationship between *in vivo* and *in vitro* response of the top 1 to 15 lipids. The analysis revealed a significant positive correlation (Pearson correlation coefficient =0.780, r =0.001, $0.001 \leq p < 0.01$) between the two variables. Note. *p< 0.05, **p<0.01, ***p<0.001. Significant correlations are flagged using one (p < 0.05), two (p< 0.01) or three (p < .001) asterisks.

a

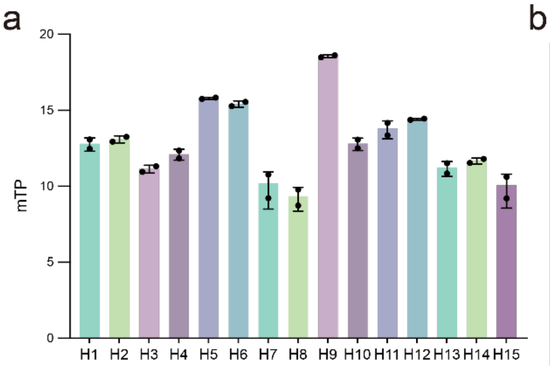

b

Pearson Correlations

		In vitro	In vivo
In vitro	Pearson Correlation	1	0.756**
	Sig. (2-tailed)		0.001
	N	15	15
In vivo	Pearson Correlation	0.756**	1
	Sig. (2-tailed)	.001	
	N	15	15

Note. *p< 0.05, **p<0.01, ***p<0.001. Significant correlations are flagged using one (p < 0.05), two (p< 0.01) or three (p < .001) asterisks.

Supplementary Figure 11. a. LNPs of the top 1 to 15 ionizable lipids were tested in C2C12 myoblast cells (n = 2). **b.** A Pearson correlation analysis was conducted by SPSS v27.0 to examine the rank relationship between C2C12 cells and the in vivo response of the top 15 lipids. The analysis revealed a significant positive correlation (Pearson correlation coefficient =0.756**) between the two variables. Note. *p< 0.05, **p<0.01, ***p<0.001. Significant correlations are marked using one (p < 0.05), two (p< 0.01) or three (p < .001) asterisks.

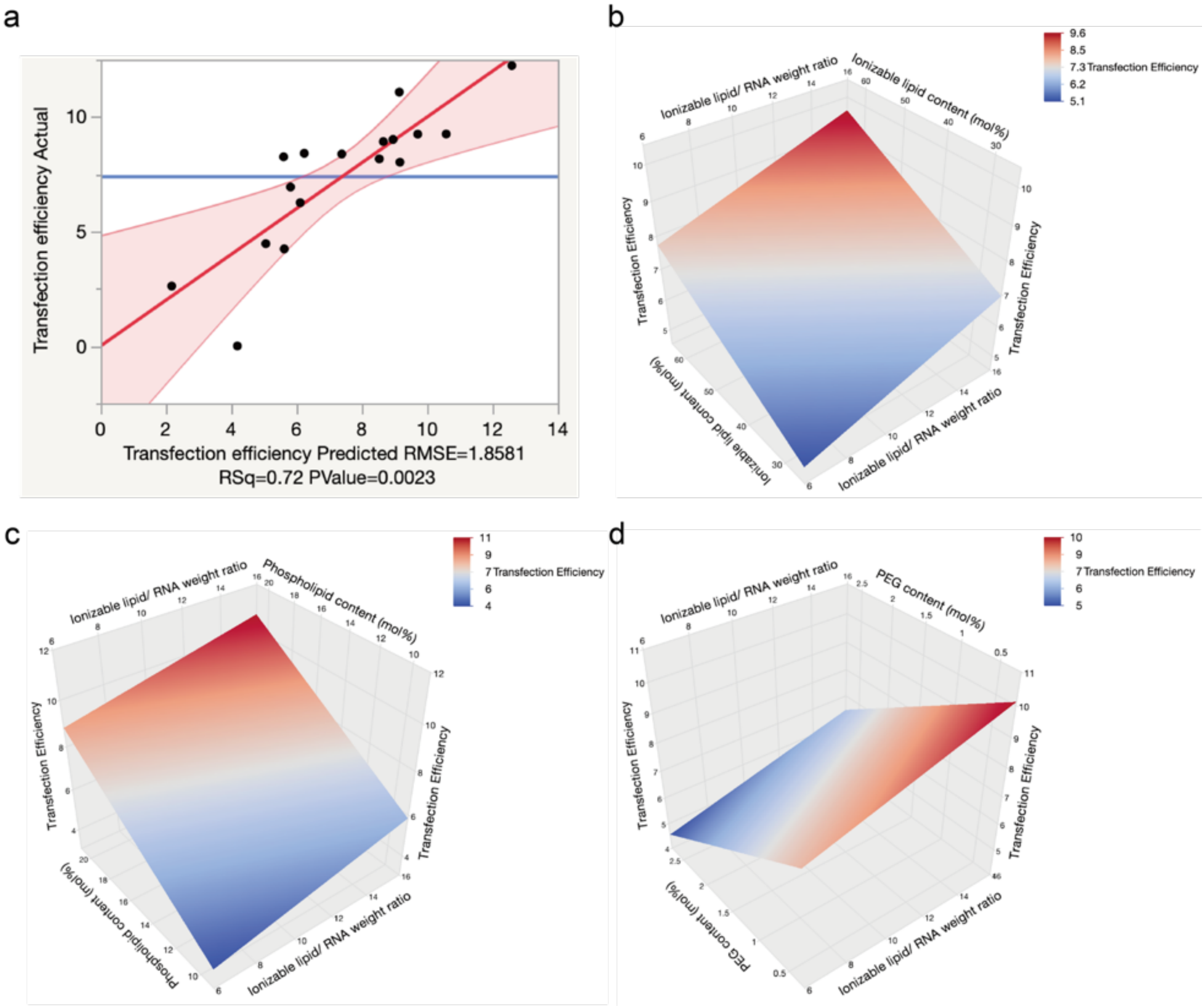

Supplementary Figure 12. LNP formulation optimization by DoE for HeLa cells. (a) The calculation for the predicted response for root means square error (RMSE) in DOE. Different response plots (b) ionizable lipid content (mol%) (c) phospholipid content (mol/%) and (d) PEG content (mol%).

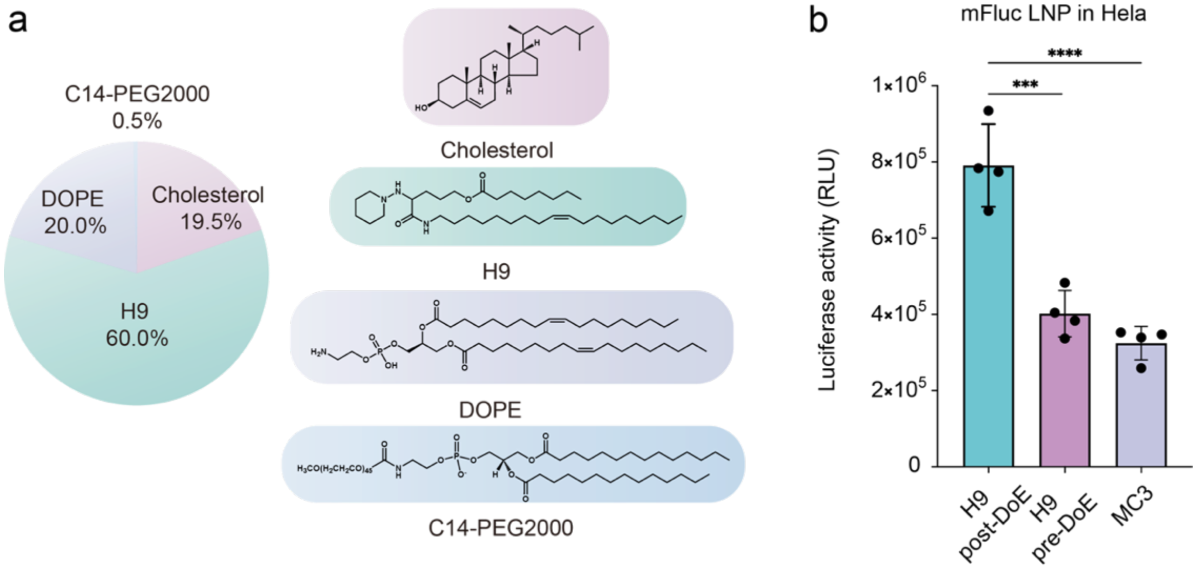

Supplementary Figure 13. (a) The top-performing formulation parameters used in the optimization of H9 LNPs in Hela. (b) Transfection of mFLuc LNPs in Hela cells (n = 4, per group).

a

	Day 0	Day 7
Size (nm)	108.0 ± 2.1	120.1 ± 4.5
PDI	0.16 ± 0.01	0.21 ± 0.02
Zeta Potential (mv)	-2.28	5.35
EE(%)	92.08 ± 2.5	82.4 ± 1.2
In vivo Transfection	7.5E+07	7.1E+07

b

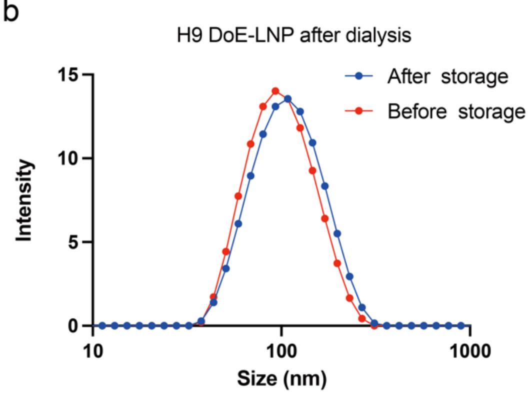

Supplementary Figure 14. Stability of H9 post-DoE LNP after dialysis. a. The physicochemical properties and response of the H9 LNP before storage and after storage at -20℃ for 1 week, 10% sucrose was added to the LNP solution before the stability test. b. Size of H9 post-DoE LNPs measured by dynamic light scattering.

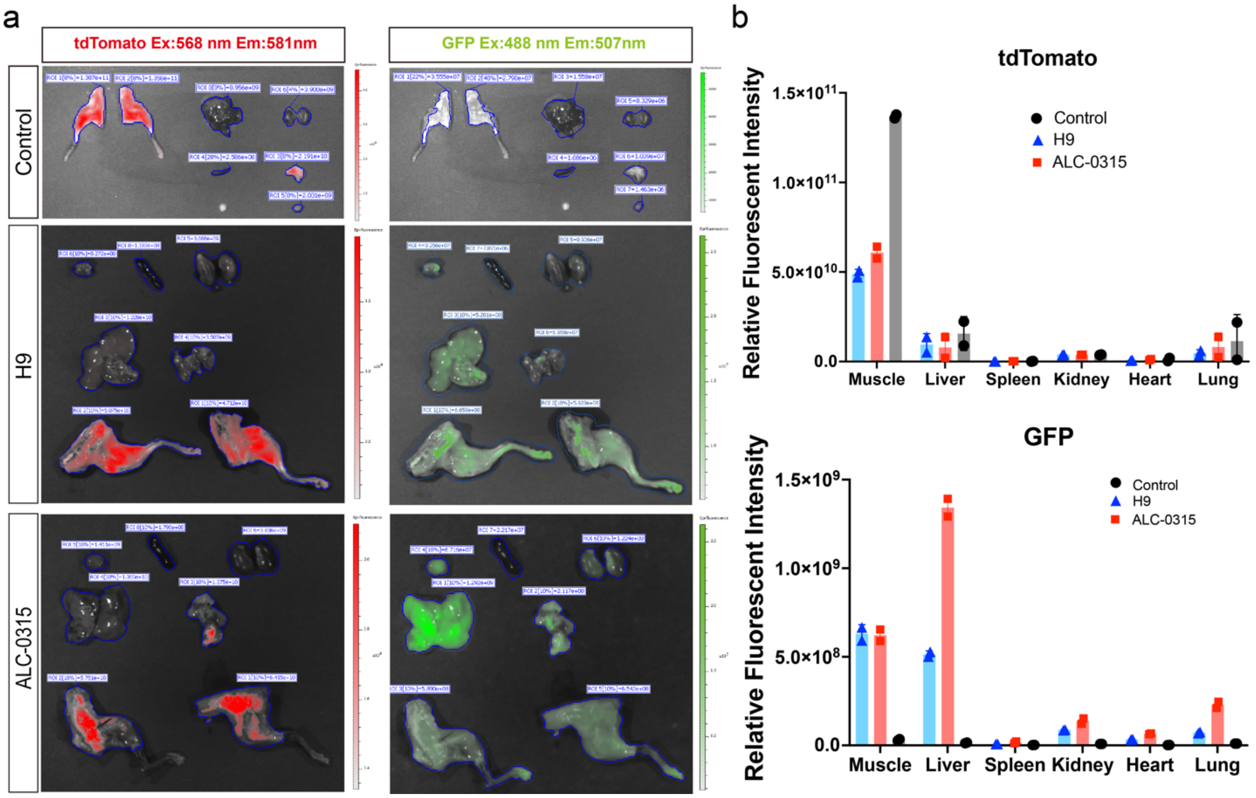

Supplementary Figure 15. (a) IVIS images of ROSA$^{mT/mG}$ Cre reporter mice organs after Cre-mRNA LNP by Intramuscular injection. (0.5 mg kg $^{-1}$ Cre-mRNA of each mouse and the control group is the homozygous ROSA$^{mT/mG}$ Cre reporter mice without any treatment, n = 2) (b) Comparison of the relative fluorescence intensity of each organ after intramuscular injection of Cre-mRNA LNP. (tdTomato: Ex:568 nm, Em:581 nm, GFP: Ex: 488 nm, Em: 507 nm).

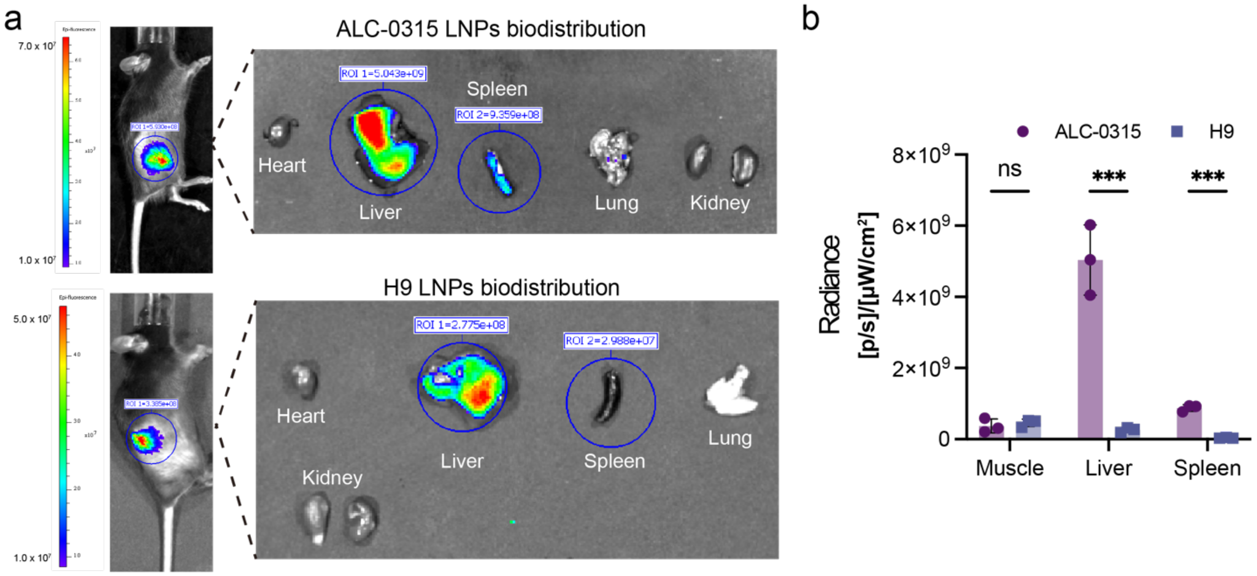

Supplementary Figure 16. (a) Representative IVIS images of C57BL/6 J mice at 6 h following intramuscular administration of ALC-0315 or H9 Cy5-mLuc-LNPs (0.3 mg/kg mLuc per mouse). (b) Quantification of Cy5 signal in muscle, liver and spleen (n = 3). Statistical significance was analyzed by the two-tailed Student's t-test. *=p-value <0.05, **=p-value<0.01, ***=p-value<0.005. Data are presented as mean±SD.

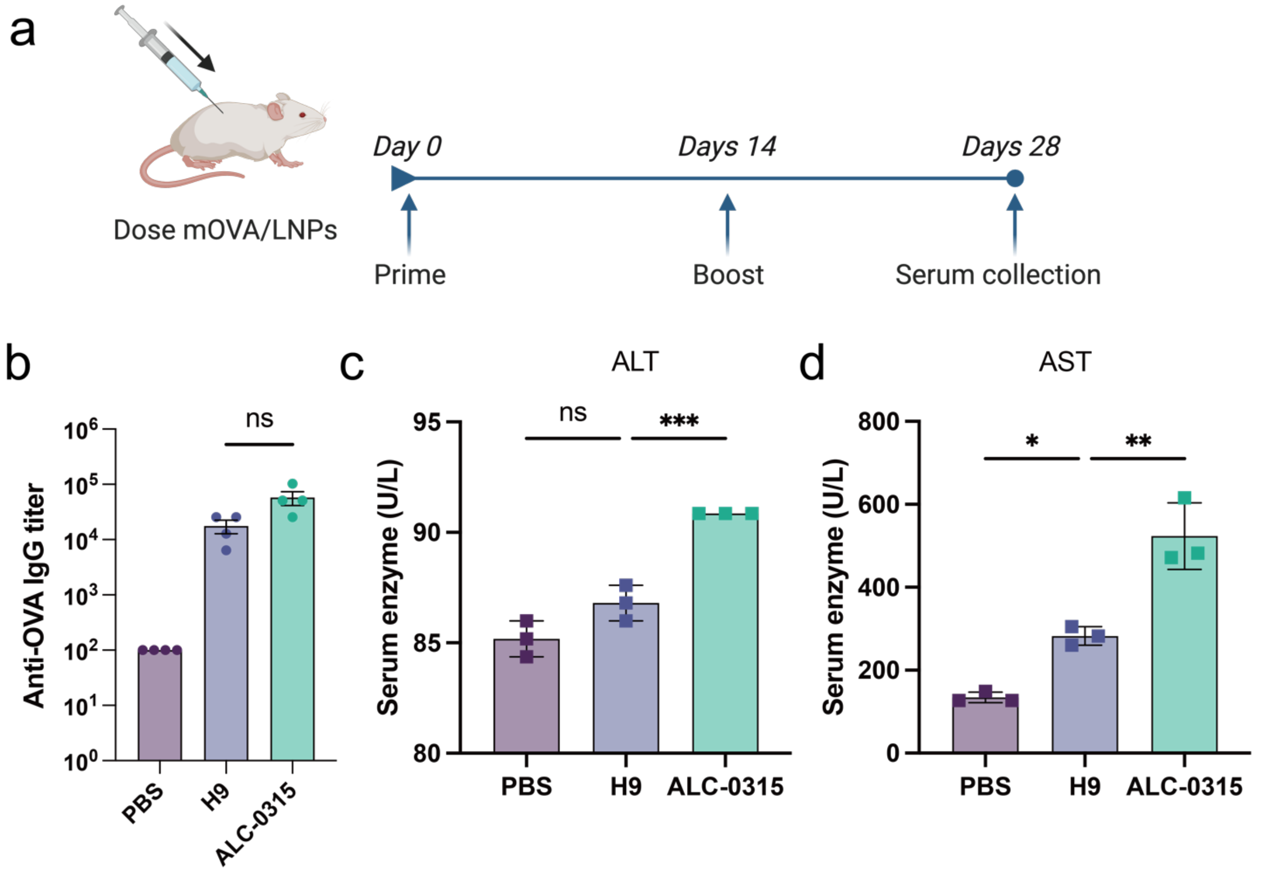

Supplementary Figure 17. (a) Timeline for mOVA/LNPs vaccination and sample collection in female BALB/c mice. (b) OVA-specific antibody titers in the mice treated with H9/mOVA (10 µg) and ALC-0315/mOVA (10 µg) on days 28. Control: unvaccinated animals (n = 4). Serum levels of liver enzymes on day 28 and quantification, (c) alanine aminotransferase (ALT, n = 3) and (d) aspartate aminotransferase (AST, n = 3). From the BALB/c mouse vendor colony documentation, the baseline of ALT was 89.0 U/L and AST was 236 U/L. (Doc: ALT 29301, AST 23903 from phenome.jax.org). Statistical significance was analyzed by using one-way analysis of variance with Tukey's multiple comparisons test. *=p-value <0.05, **=p-value<0.01, ***=p-value<0.005. Data are presented as mean±SD. Figure (a) was created with BioRender.com and released under a Creative Commons Attribution-NonCommercial-NoDerivs 4.0 International license.

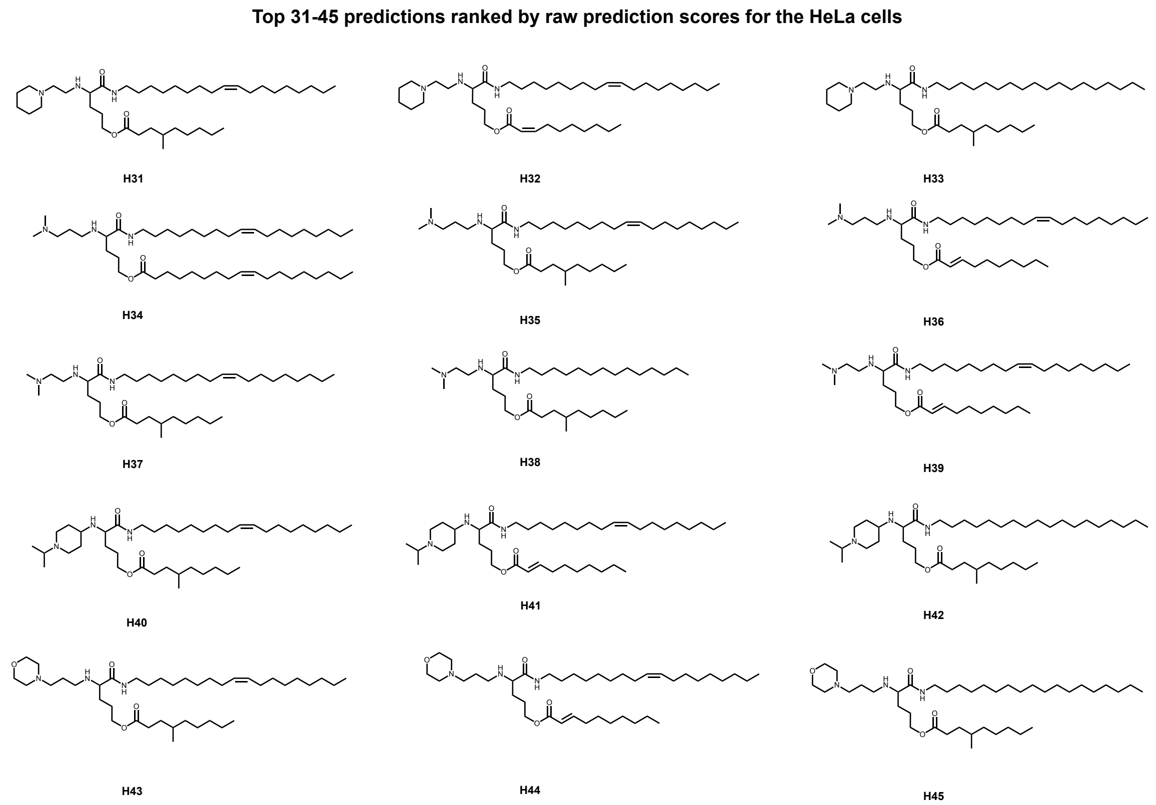

Supplementary Figure 18. Top 31-45 lipid candidate structures identified for Hela cells by the AGILE model.

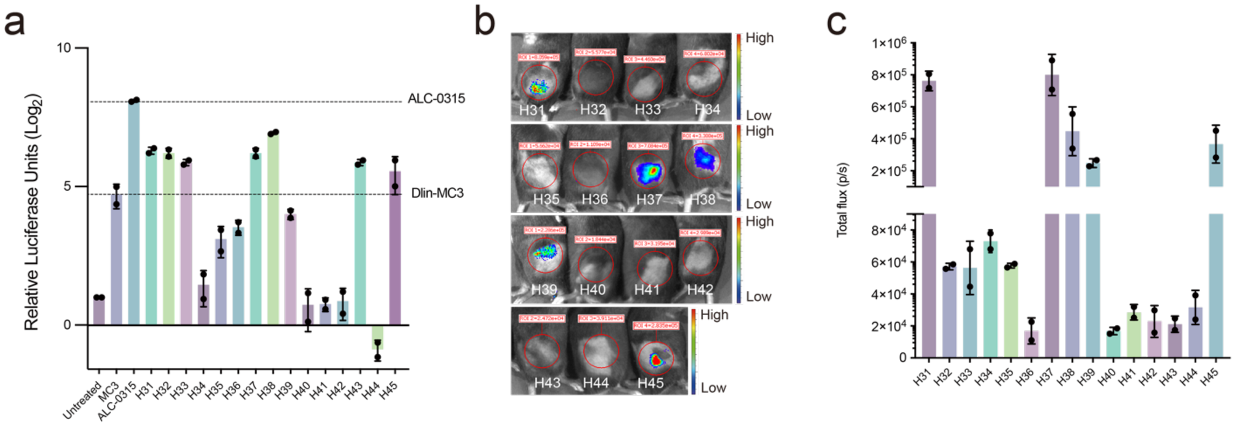

Supplementary Figure 19. The top 31 to 45 ionizable lipids identified by AGILE were rapidly synthesized and screened *in vitro* and *in vivo*. a. LNPs of the top 31 to 45 ionizable lipids were tested in HeLa cells. b. Representative IVIS images of the first-round *in vivo* testing (intramuscular injection, 0.2 mg mRNA/kg per mouse) from two mice per group and the Fluc expression was visualized at 6 h. c. histogram analysis of total flux in muscles. Note. *p< 0.05, **p<0.01, ***p<0.001. Significant correlations are marked using one (p < 0.05), two (p< 0.01) or three (p < .001) asterisks.

a

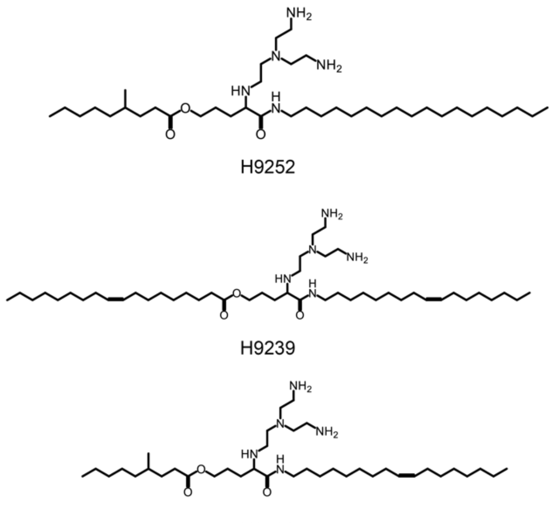

b

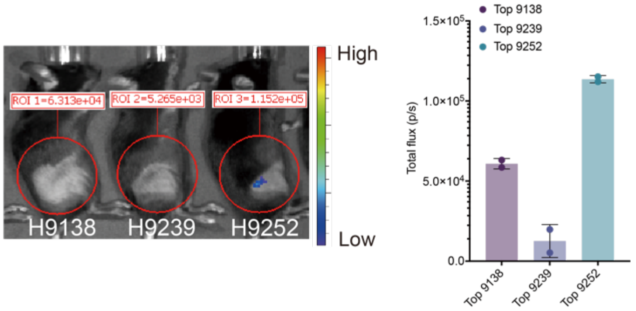

c

LNP	Size (nm)	PDI	In vitro (HeLa, Log_2)	In vivo (I.M.)
H9252	130.2	0.20	0.55	6.313E+04
H9239	132.6	0.22	0.23	5.265E+03
H9138	120.5	0.15	1.24	1.152E+05

Supplementary Figure 20. The 3 bottom-ranked candidates identified by AGILE. a. Structures of H9252, H9239, H9138. **b.** Representative IVIS images of the first-round *in vivo* testing (intramuscular injection, 0.2 mg mRNA/kg per mouse) from two mice per group and the Fluc expression was visualized at 6 h. **c.** Table for the characterization of LNPs and their *in vitro* (mTP in HeLa) and *in vivo* (I.M.) mFluc transfection performance.

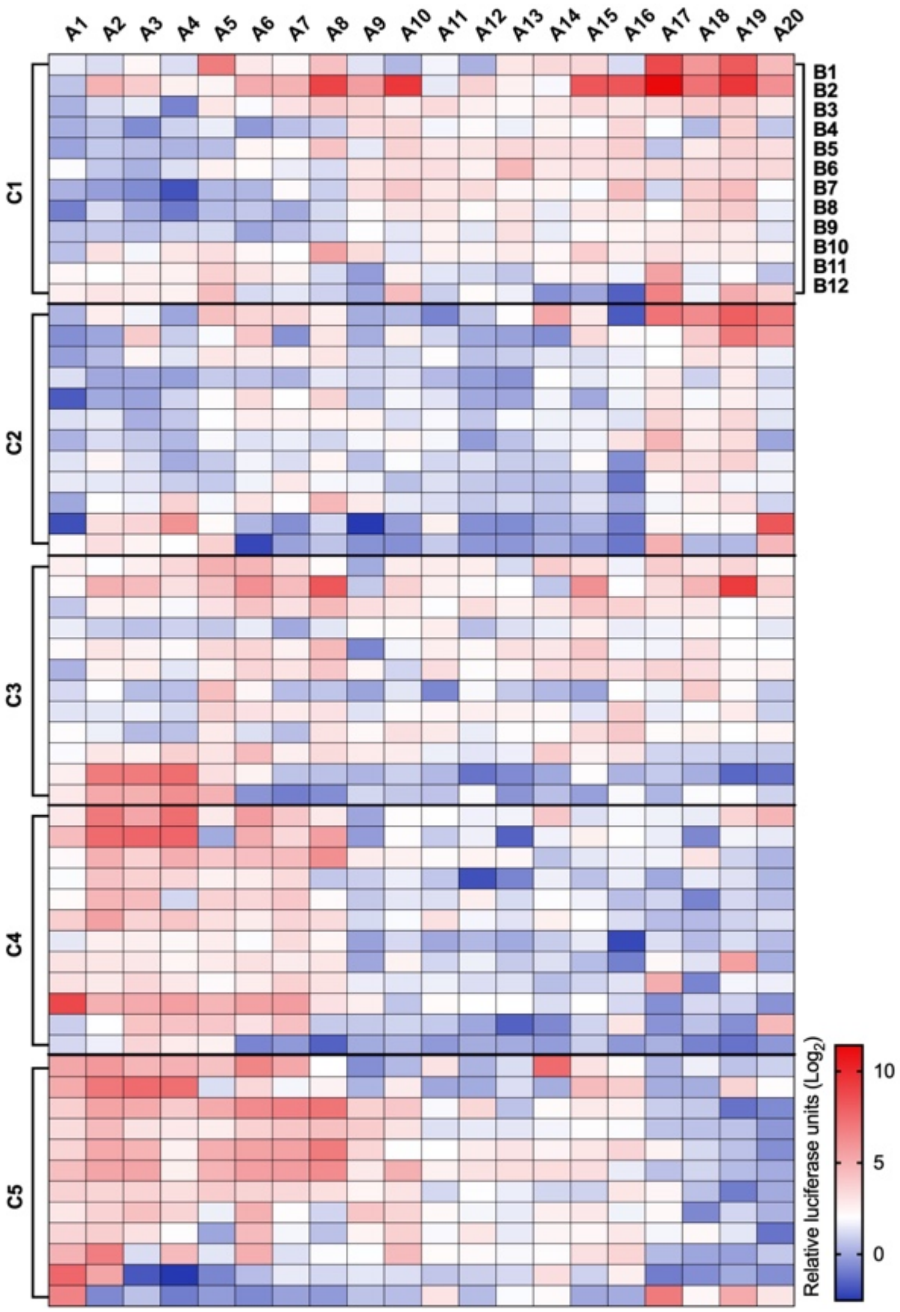

Supplementary Figure 21. The data used for the fine-tuning are depicted in a heatmap, which involved 1,200 LNPs for Fluc mRNA (mFLuc) delivery and measuring the relative luciferase expression in RAW 264.7 cells.

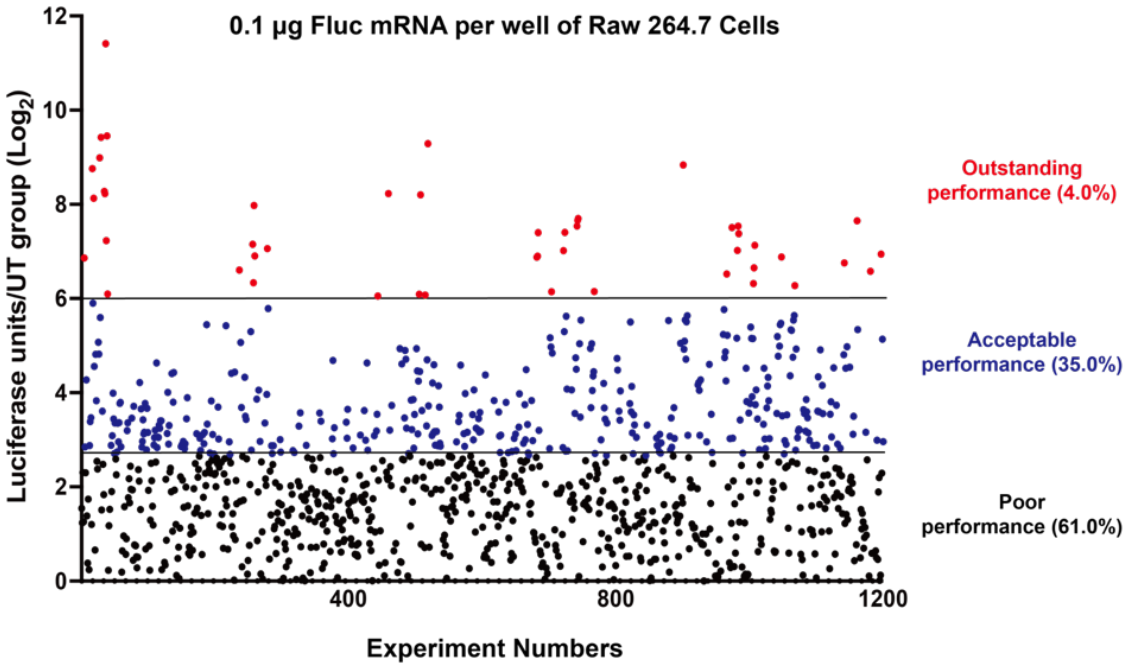

Supplementary Figure 22. Luciferase activity/untreated are shown as scatter plots for the 1,200 LNPs in RAW 264.7.

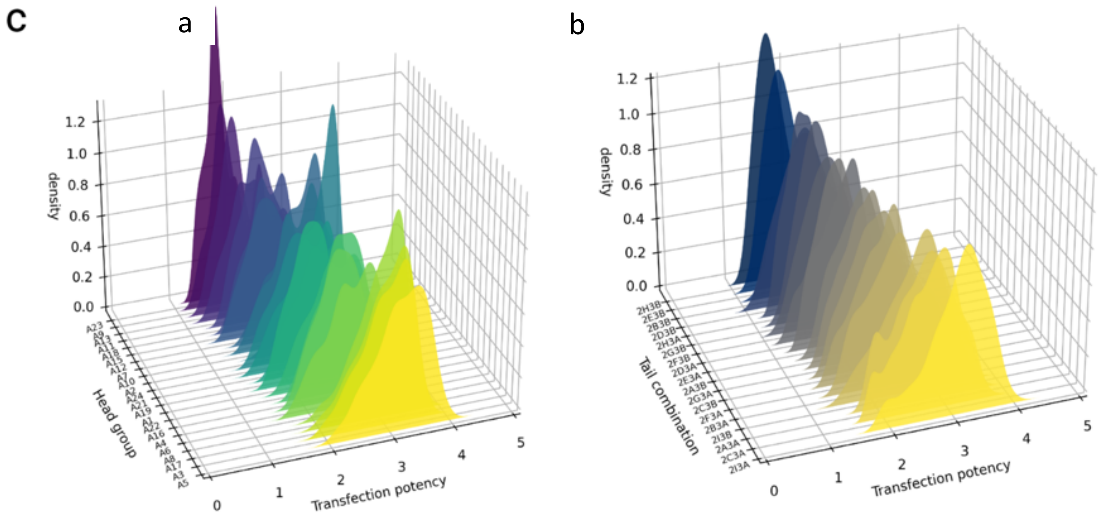

Supplementary Figure 23. The stratified distribution plots for predicted potencies across different categories. (a) Moving to the RAW 264.7 cell line, the distribution plot of predicted potencies stratified by head groups. (b) Distribution plot of predicted potencies for the RAW 264.7 cell line, stratified by tail combinations.

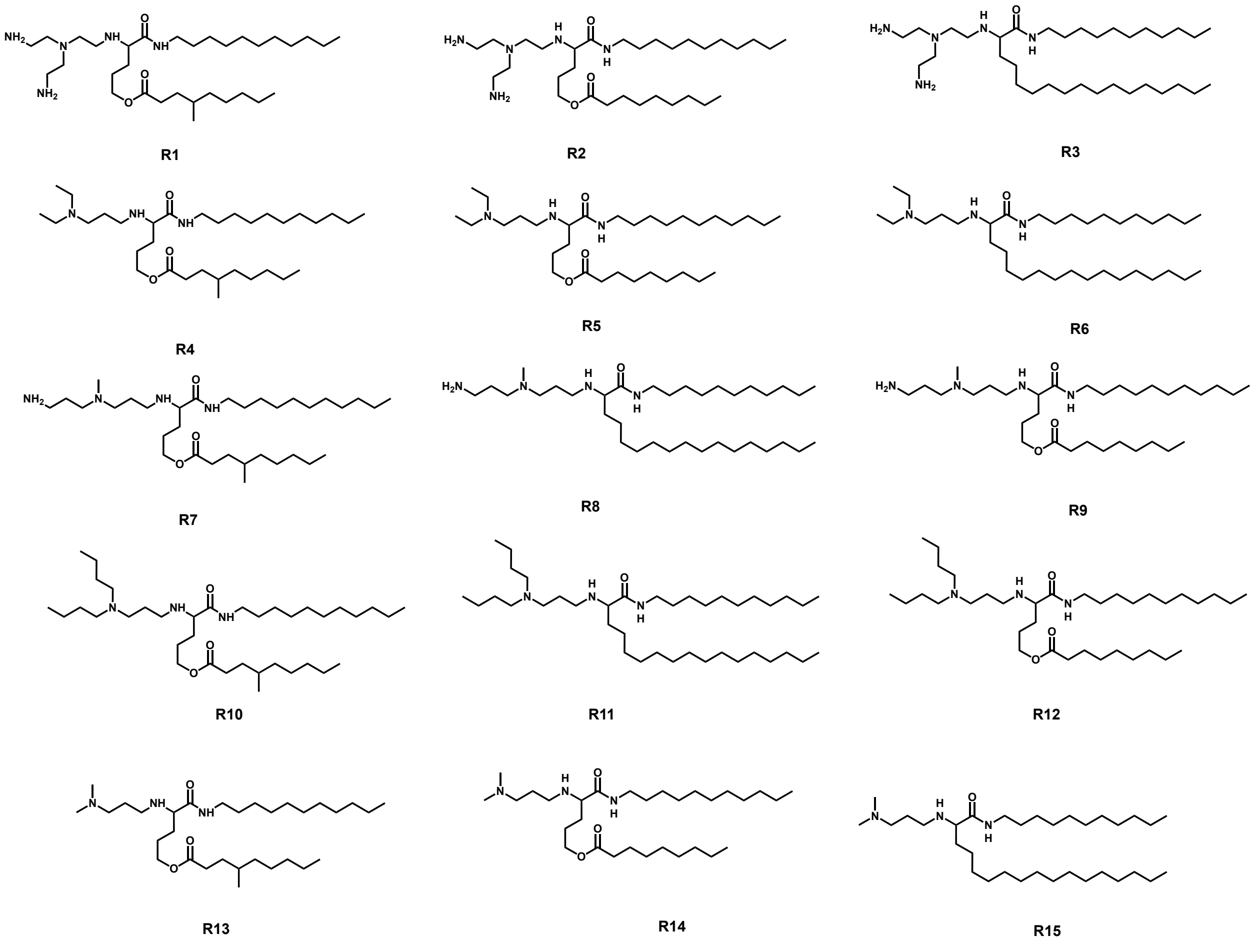

Supplementary Figure 24. Top 1-15 lipid candidates identified for RAW 264.7 cells by the AGILE model.

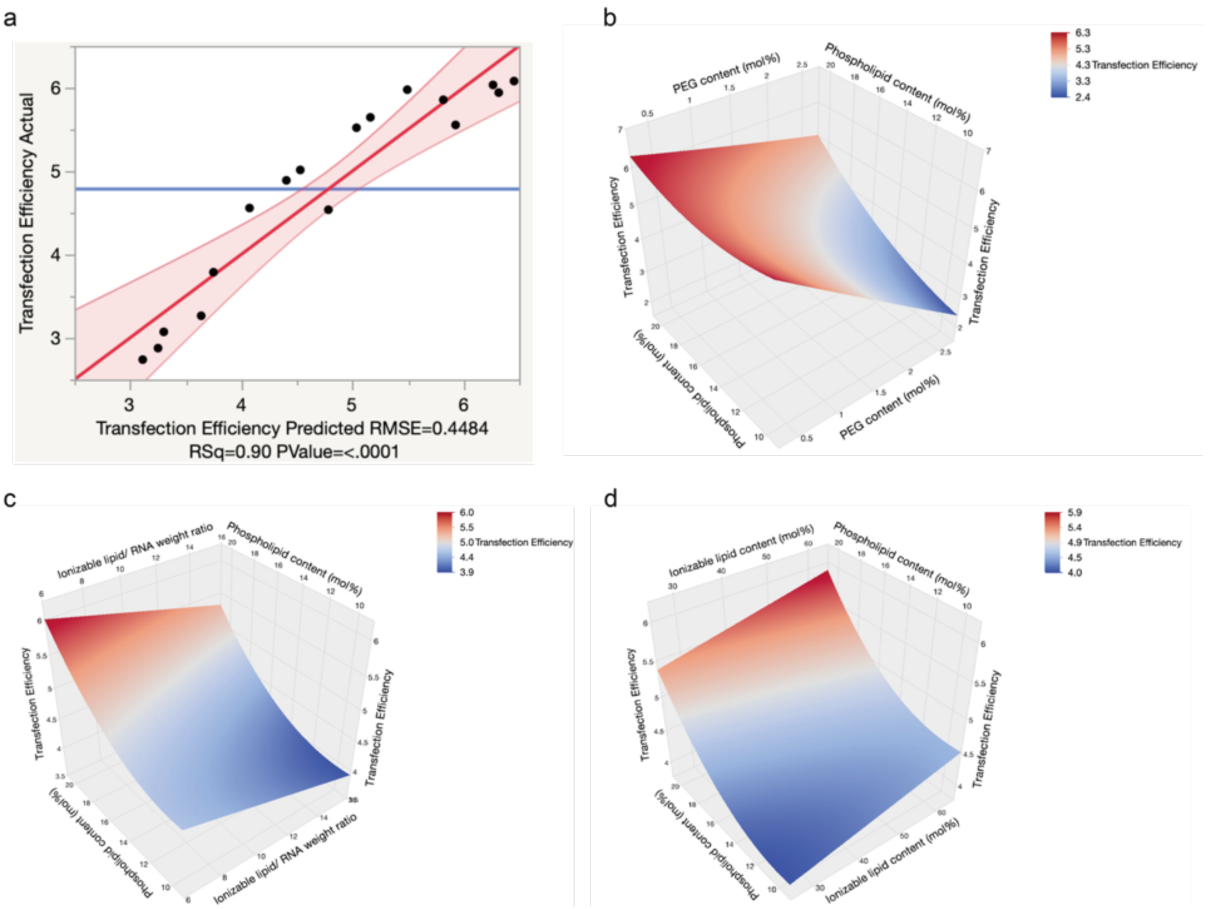

Supplementary Figure 25. LNP formulation optimization by DoE for RAW 264.7 cells. (a) The calculation for the predicted response for root means square error (RMSE) in DOE. Different response plots (b) PEG content (mol%) (c) phospholipid content (mol/%) and (d) ionizable lipid content (mol%).

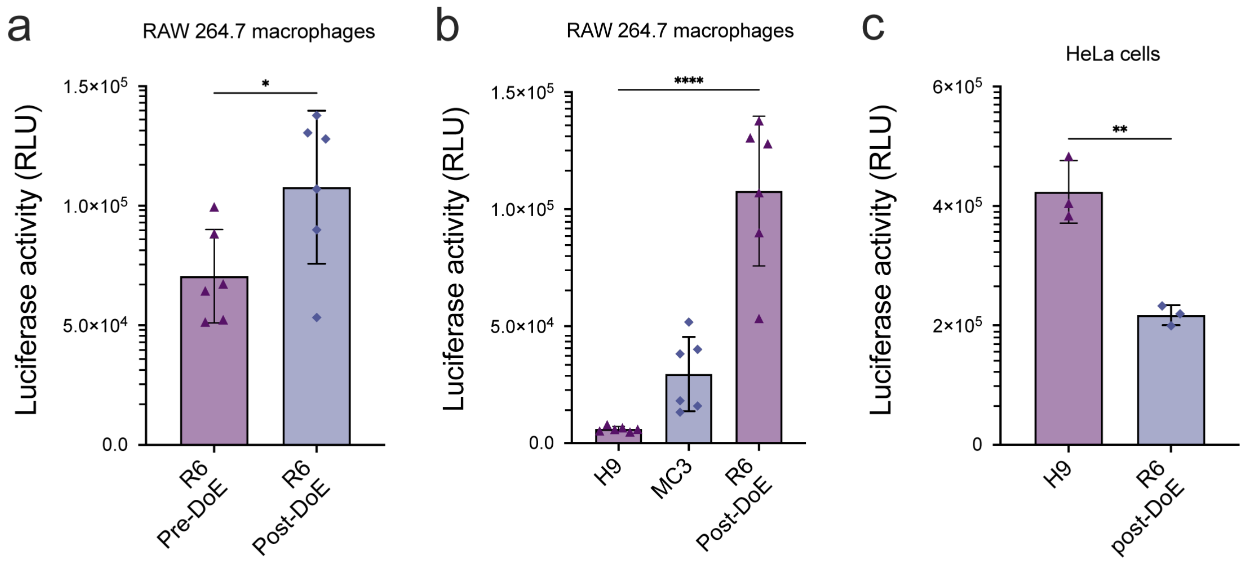

Supplementary Figure 26. (a) Comparison of the luciferase activity of the R6 LNPs Pre-DoE and Post-DoE in RAW 264.7 macrophages (n = 6). (b) Comparison of the luciferase activity of the R6 LNPs Post-DoE, MC3 and H9 LNPs in RAW 264.7 macrophages (n = 6). (c) Comparison of the luciferase activity of the R6 LNPs Post-DoE and H9 LNPs in HeLa cells. All H9 LNPs were optimized formulations after DoE (n =3). Statistical significance was analyzed by the two-tailed Student's t-test. *= p <0.05, **=p <0.01, ***=p <0.005. Data are presented as mean ± SD.

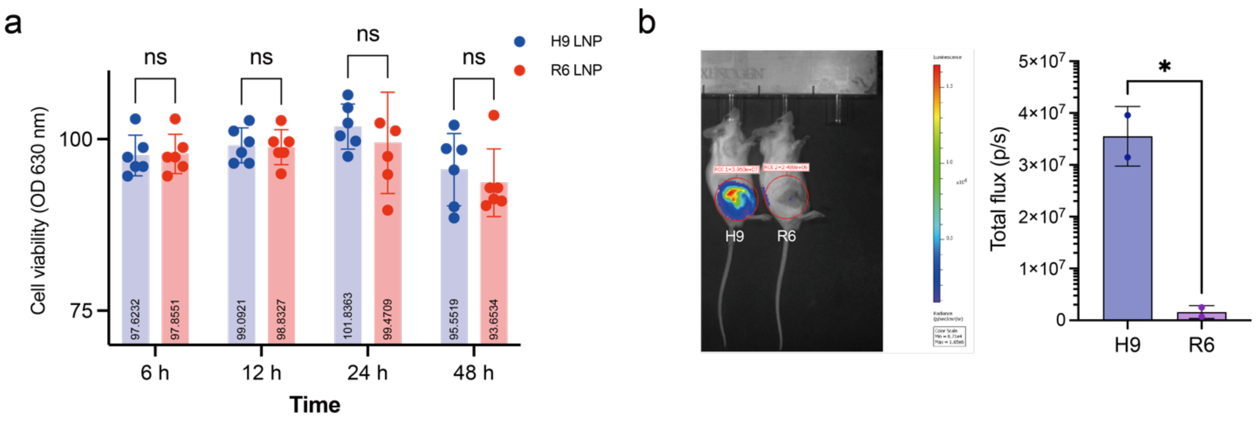

Supplementary Figure 27. (a). HeLa cells were incubated for different time points (6, 12, 24 and 48 h) post-transfection before analysis where cell viability was determined according to QUANTI-Blue™ assay protocol (n = 6). No significant cytotoxicity was observed between H9 and R6 LNPs. Statistical significance was analyzed by using one-way analysis of variance with Tukey's multiple comparisons test. *=p-value <0.05, **=p-value<0.01, ***=p-value<0.005. Data are presented as mean±SD. (b). Representative IVIS images of the first-round in vivo testing (intramuscular injection, 0.2 mg mRNA/kg per mouse) from two mice per group and the Fluc expression was visualized at 6 h. Statistical significance was analyzed by the t-test. *= p-value <0.05, **=p-value<0.01, ***=p-value<0.005. Data are presented as mean±SD.

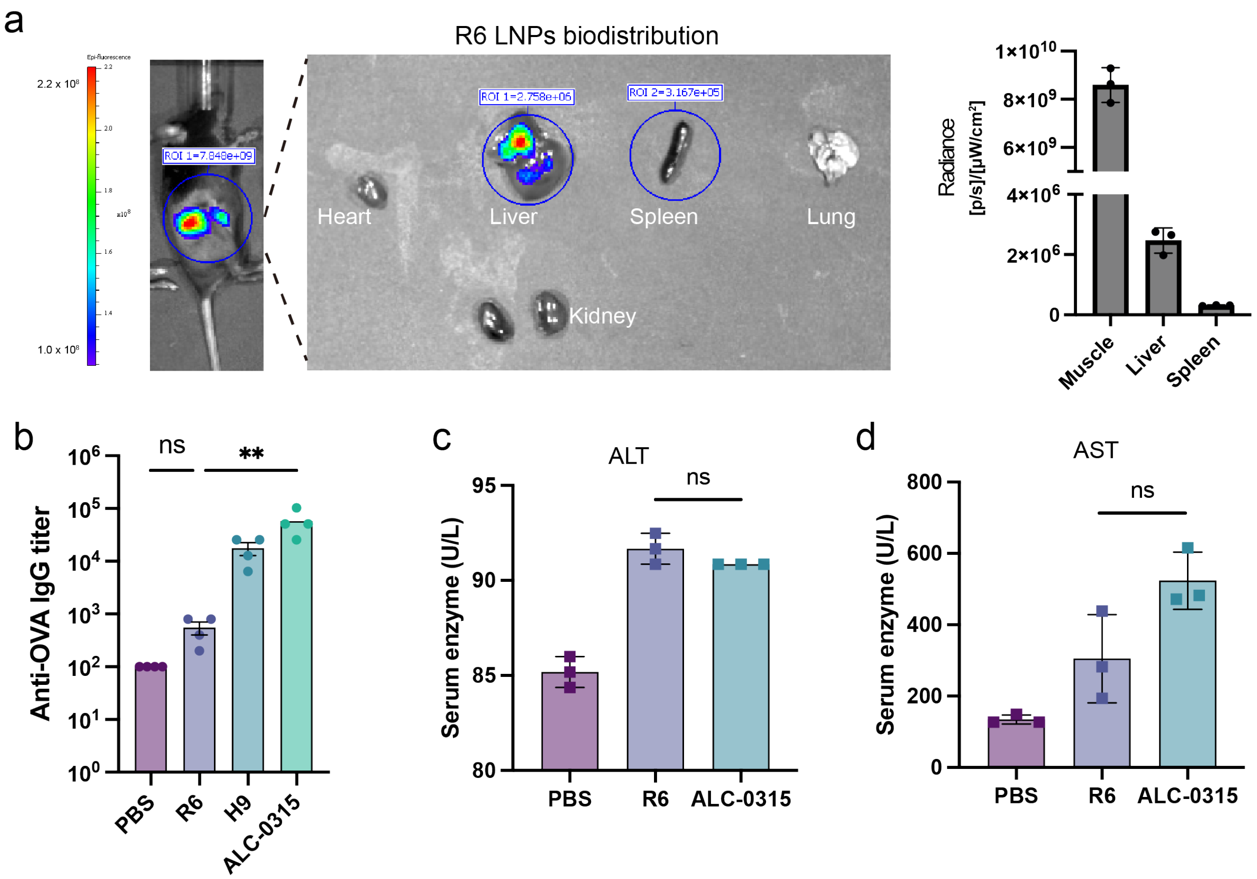

Supplementary Figure 28. (a) Representative IVIS images of C57BL/6 J mice at 6 h following intramuscular administration of R6 Cy5-mLuc-LNPs (0.3 mg/kg mLuc per mouse). (b) OVA-specific antibody titers in the mice treated with H9/mOVA (10 µg), R6/mOVA (10 µg) and ALC-0315/mOVA (10 µg) on days 28. Quantification of Cy5 signal in muscle, liver, and spleen. ($n = 3$). Serum levels of liver enzymes on day 28 and quantification, (c) serum alanine aminotransferase (ALT, $n = 3$), and (d) serum aspartate aminotransferase (AST, $n = 3$). Control: unvaccinated animals ($n = 4$). Statistical significance was analyzed by using a one-way analysis of variance with Tukey's multiple comparisons test. *=p-value <0.05, **=p-value<0.01, ***=p-value<0.005. Data are presented as mean±SD.

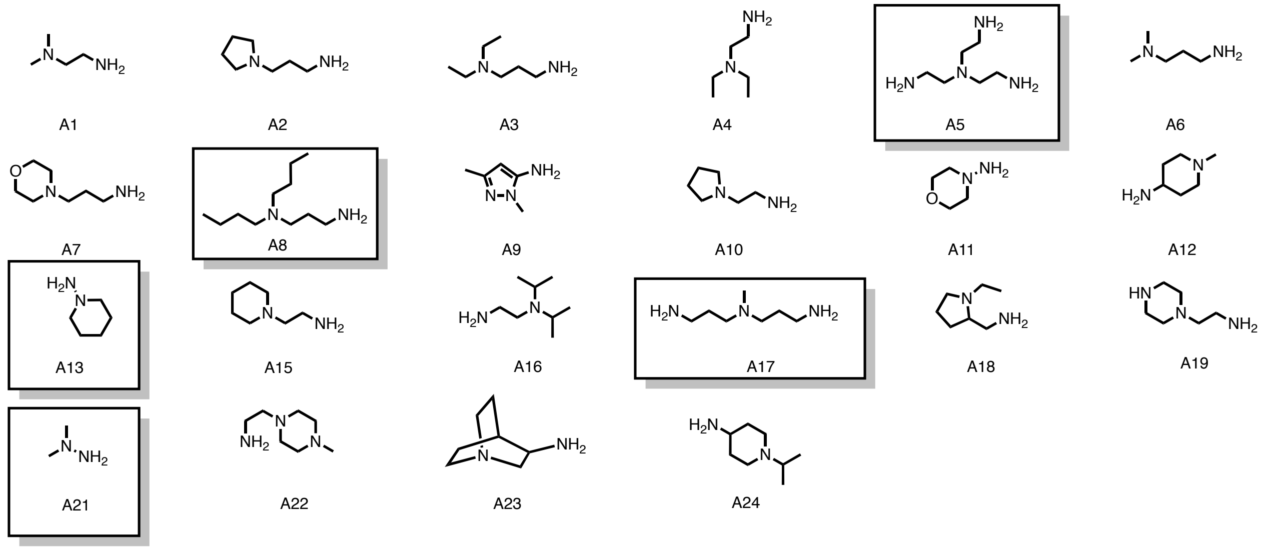

Supplementary Figure 29. All 22 unique headgroups are included in the candidate library. The amine headgroups highlighted in the boxes (A8, A13, and A21) are those that AGILE predicted would be the most effective headgroups, whereas headgroups A5 and A17 were expected to perform poorer based on AGILE's predictions.

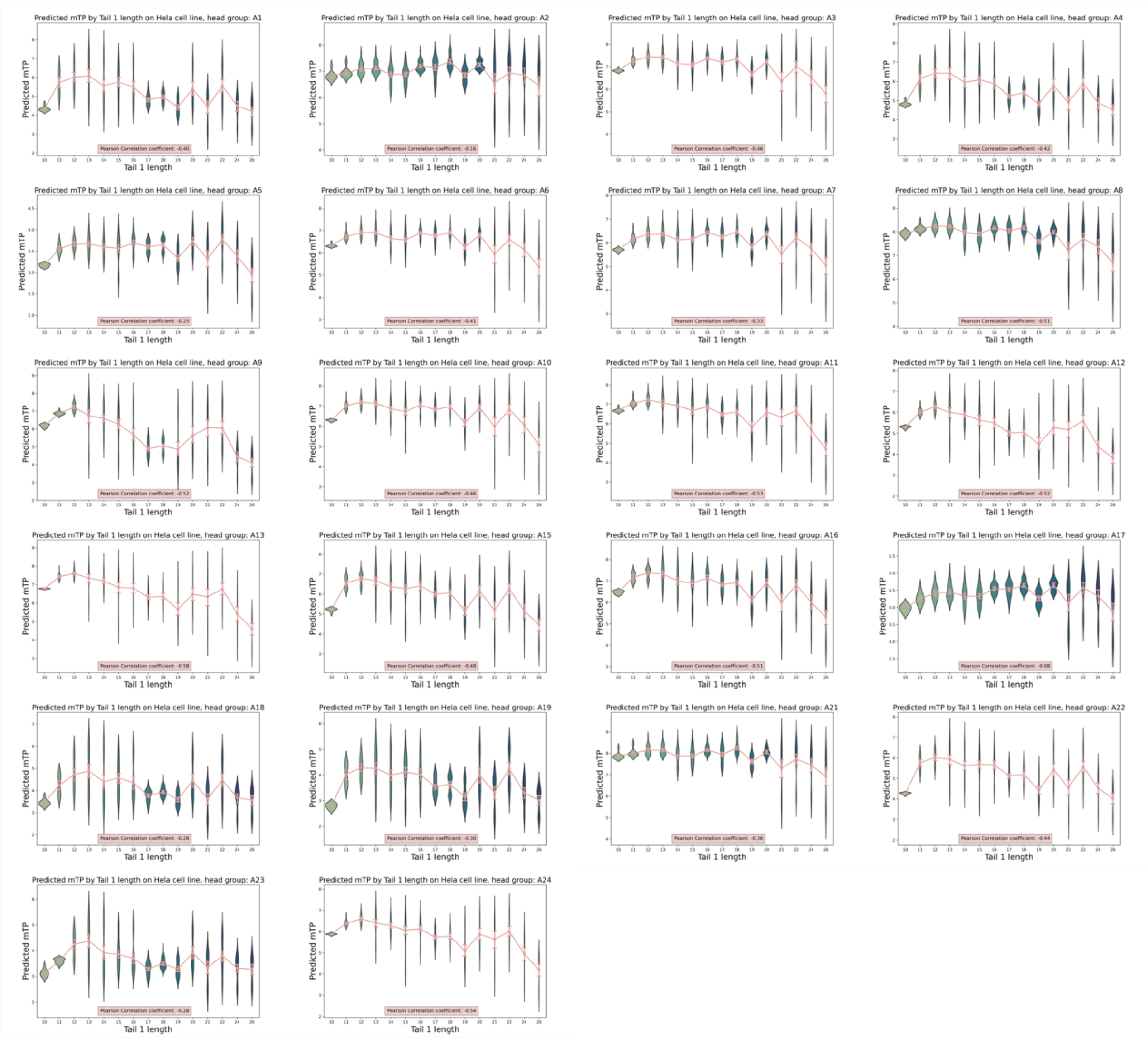

Supplementary Figure 30. Violin plot that visualizes the distribution of predicted potencies for the Hela cell line, categorized based on varying the length of tail 1 in lipids of each head group. Despite the distinct patterns exhibited by different head groups, a shared trend can be observed: an increase in the tail 1 carbon chain length from 10 to 12 correlates with an increase in predicted potency. Conversely, any subsequent lengthening of the tail 1 chain tends to have a detrimental effect on the predicted potencies.

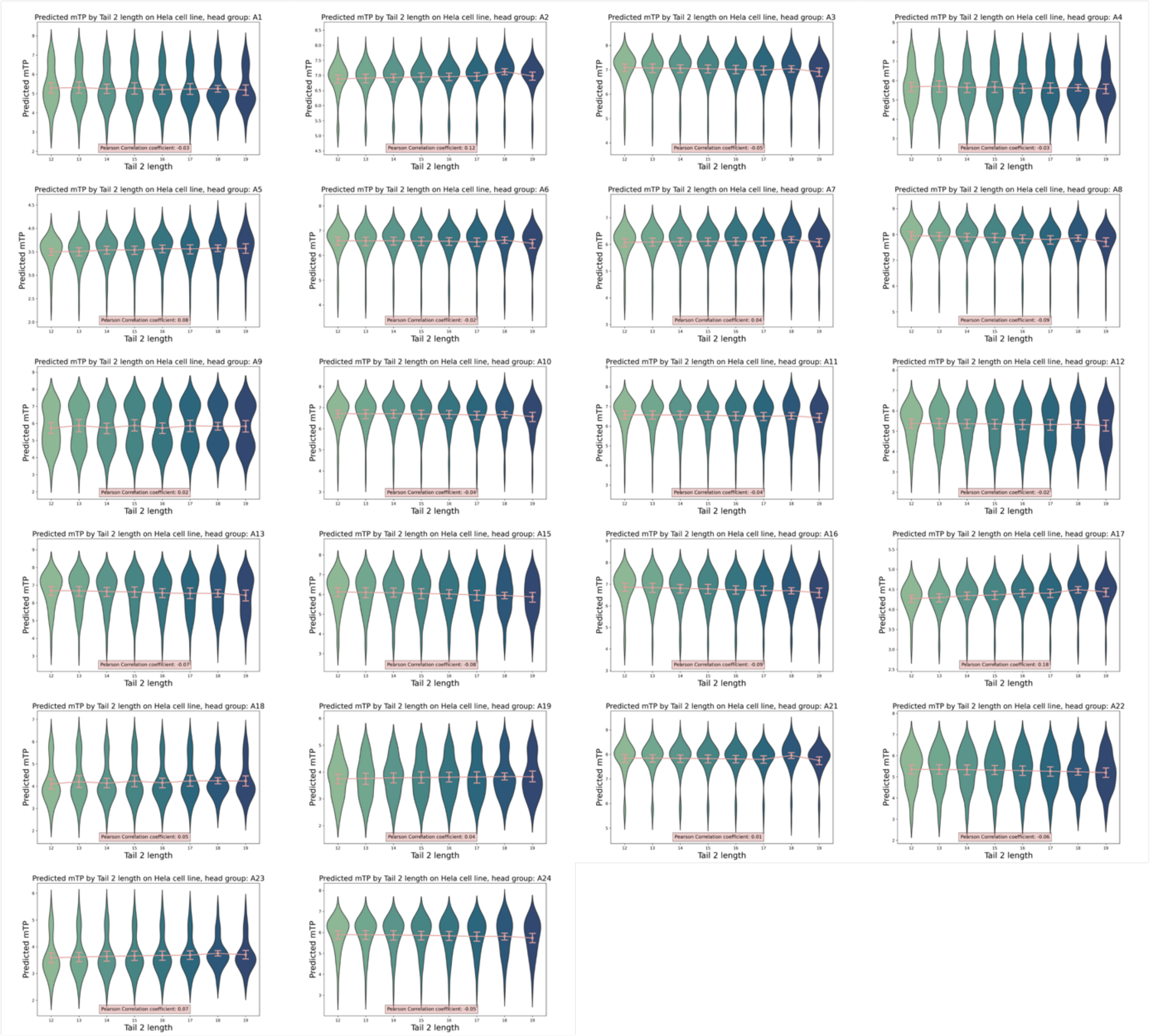

Supplementary Figure 31. Violin plot that visualizes the distribution of predicted potencies for the Hela cell line, categorized based on varying the length of tail 1 in lipids of each head group. The figure reveals that alterations in tail 2's length have a minimal effect on predicted potencies.

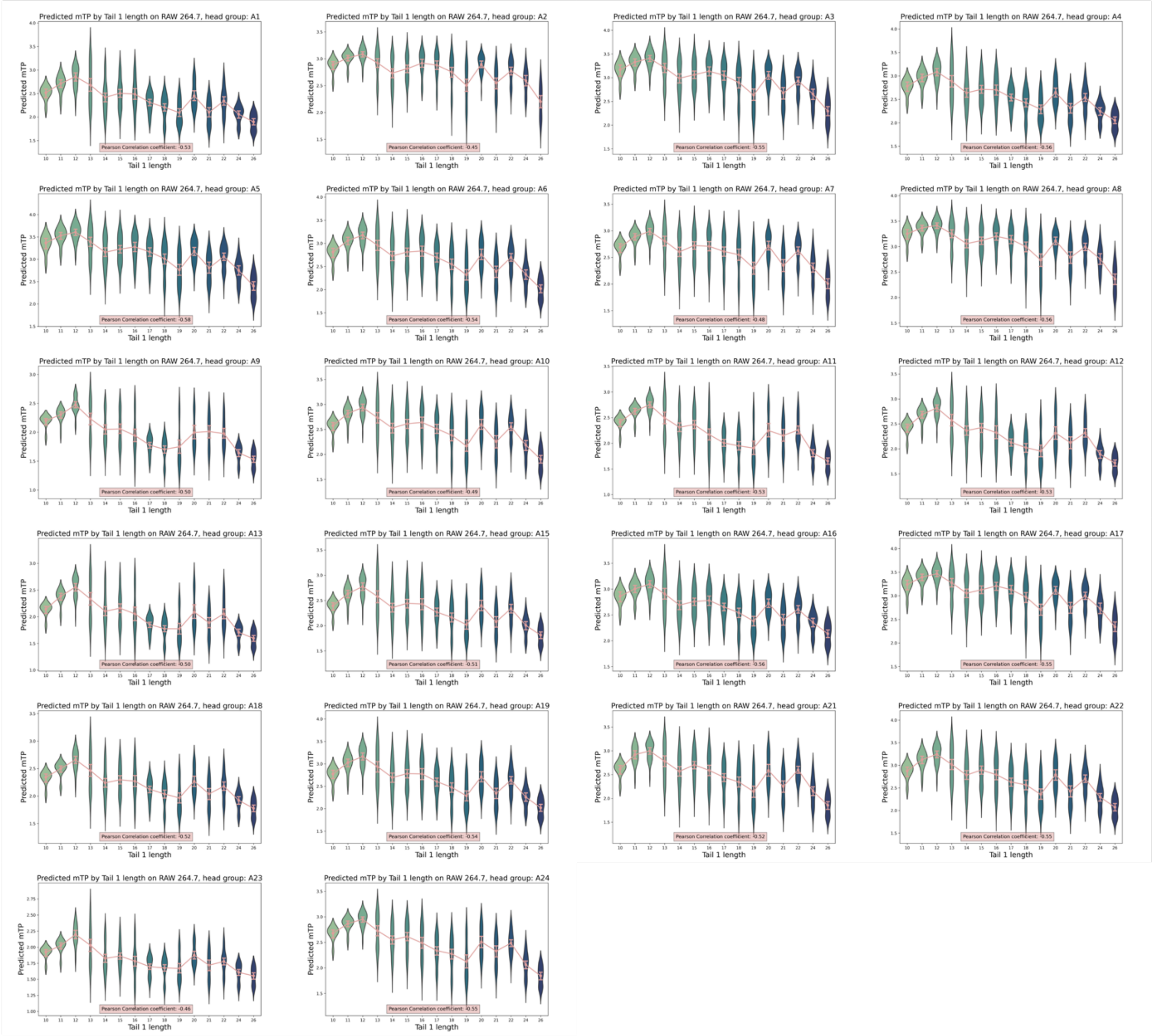

Supplementary Figure 32. Violin plot that visualizes the distribution of predicted potencies for RAW 264.7, categorized based on varying the length of tail 1 in lipids of each head group. Two shared trends can be observed: (1) An increase in tail 1's length from 10 carbons to 12 carbons correlates with an increase in predicted potencies. Conversely, any subsequent lengthening of tail 1 tends to have a detrimental effect on the predicted potencies. (2) Reduced lengths of tail 1 (C ≤ 12) exhibit less fluctuation in potency predictions compared to their longer counterparts (C>12).

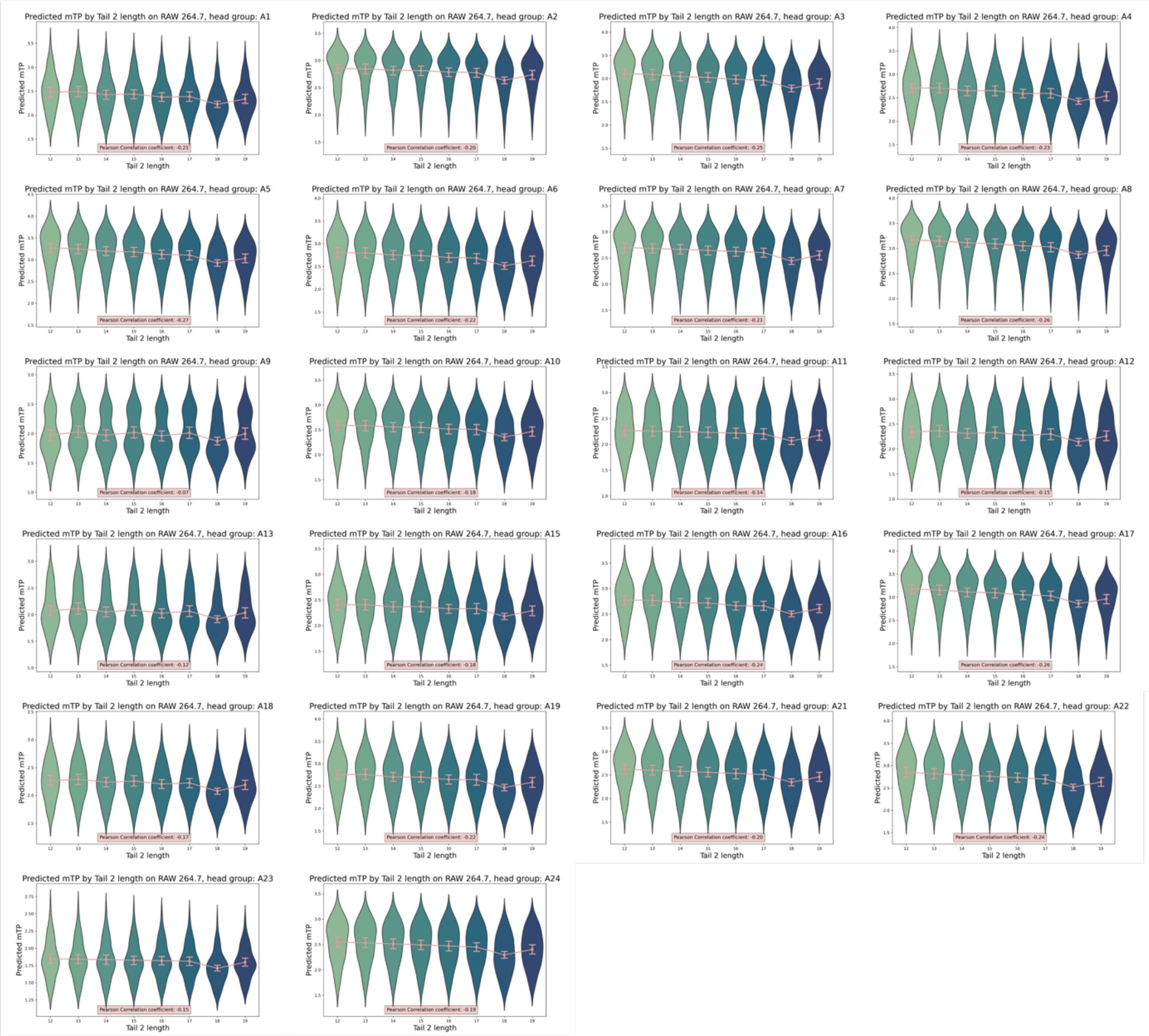

Supplementary Figure 33. Violin plot that visualizes the distribution of predicted potencies for RAW 264.7, categorized based on varying tail 2 lengths from lipids of each head group. The figures show a shared trend, where smaller tail 2 lengths are associated with higher predicted potencies.

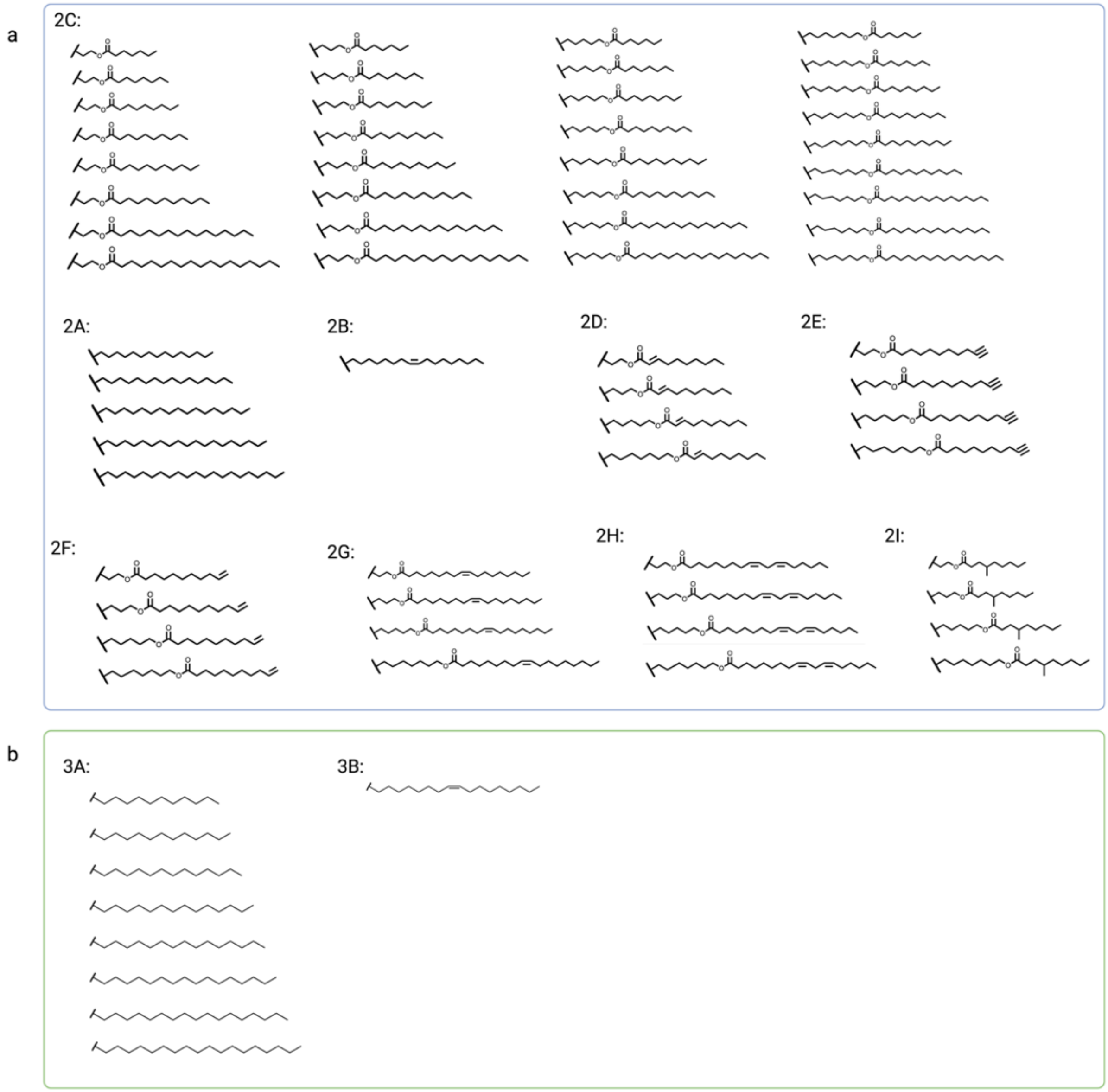

Supplementary Figure 34. Comprehensive representation of all distinct tail combinations in the candidate library. (a) Showcases different types of tail 1. (b) Showcases different types of tail 2.

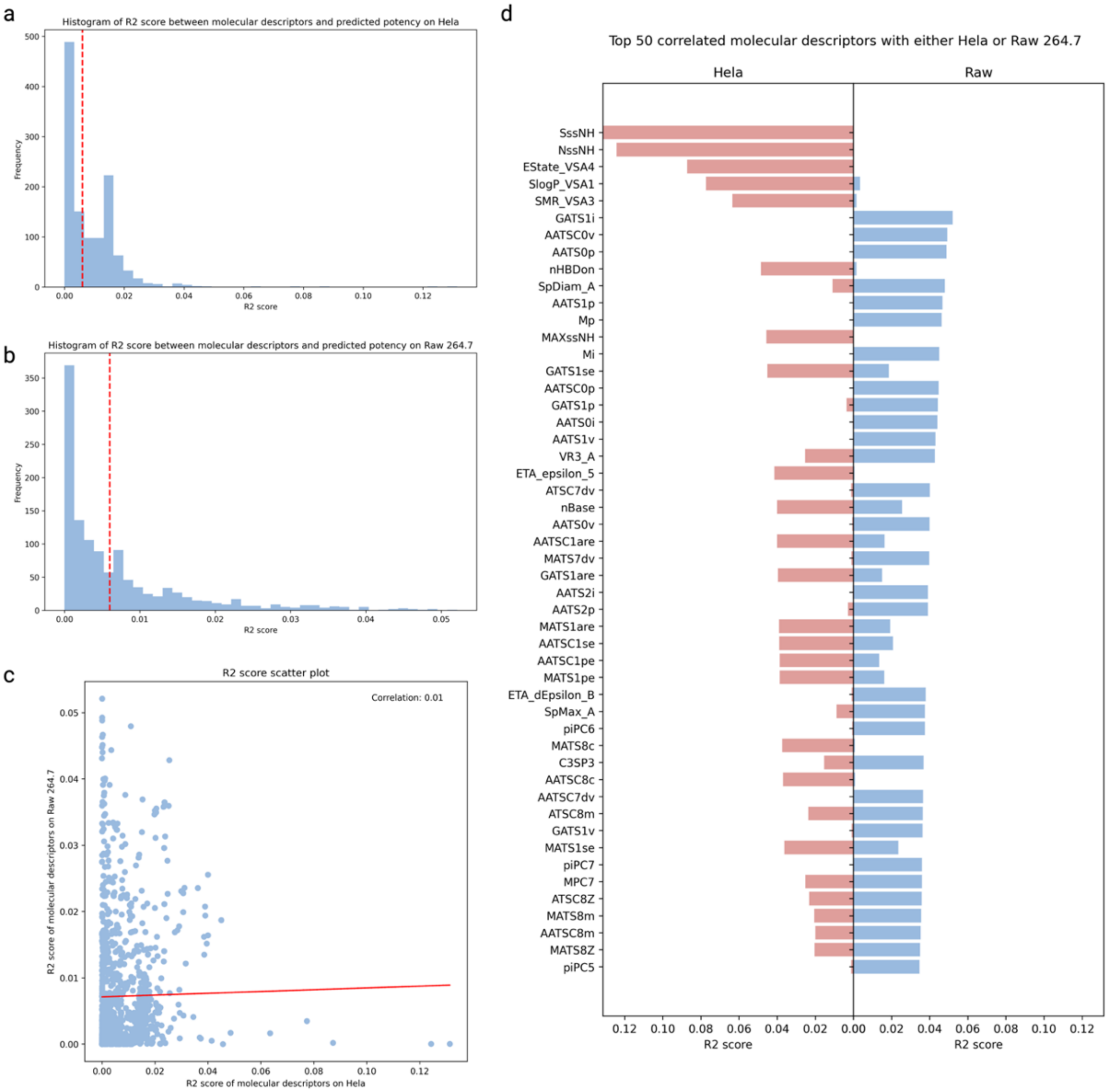

Supplementary Figure 35. (a) A histogram illustrating the R^2 score between molecular descriptors and potency labels in the Hela cell line. The red dashed line signifies the 0.006 threshold, established for descriptor selection. (b) Similarly, a histogram is displayed for the R^2 score between molecular descriptors and potency labels in the RAW 264.7 cell line, again with the red dashed line denoting the 0.006 threshold for descriptor selection. (c) A scatter plot presents the R^2 score of molecular descriptors with potency labels on both Hela and RAW 264.7 cell lines, with values on the two axes. A correlation of 0.01 indicates that molecular descriptors demonstrate a distinct correlation pattern for the Hela cell line and RAW 264.7. (d) Lastly, the top 50 selected molecular descriptors are presented, along with their respective correlation values.

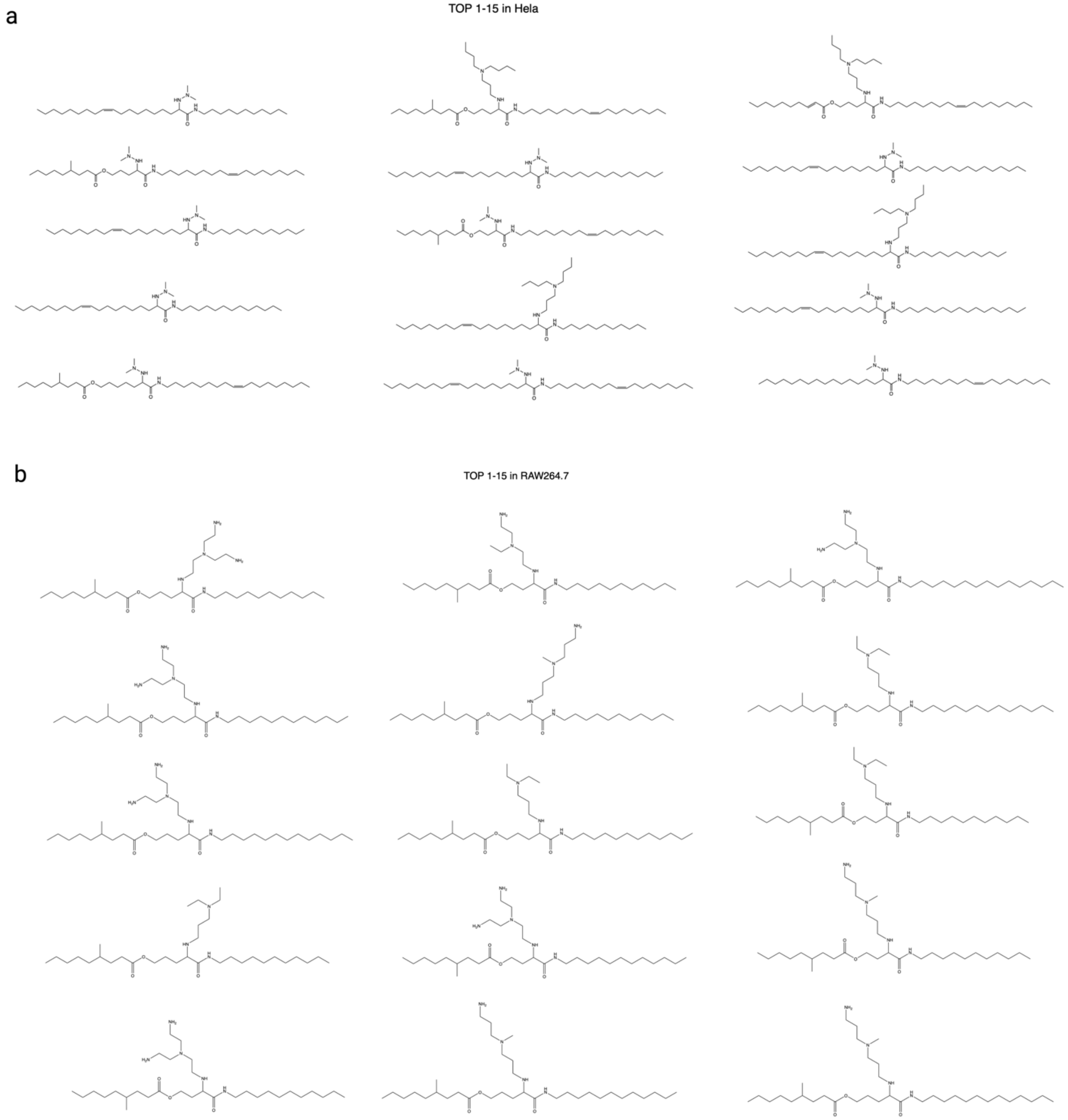

Supplementary Figure 36. Figure illuminating the significance of our head and tail-wise ranking scheme in enhancing structural diversity, exemplified by the top 15 predictions chosen based solely on predicted scores without the application of our ranking method. (a) The top 15 predictions selected for the Hela cell, without the implementation of our ranking scheme, are displayed. These selections reveal limited head group diversity, as only two head groups are selected, A8 and A21. (b) The top 15 predictions chosen for the RAW 264.7 cell line, again without our ranking scheme, are shown. These selections also exhibit restricted head group diversity, with only three different head groups represented: A3, A5, and A17.

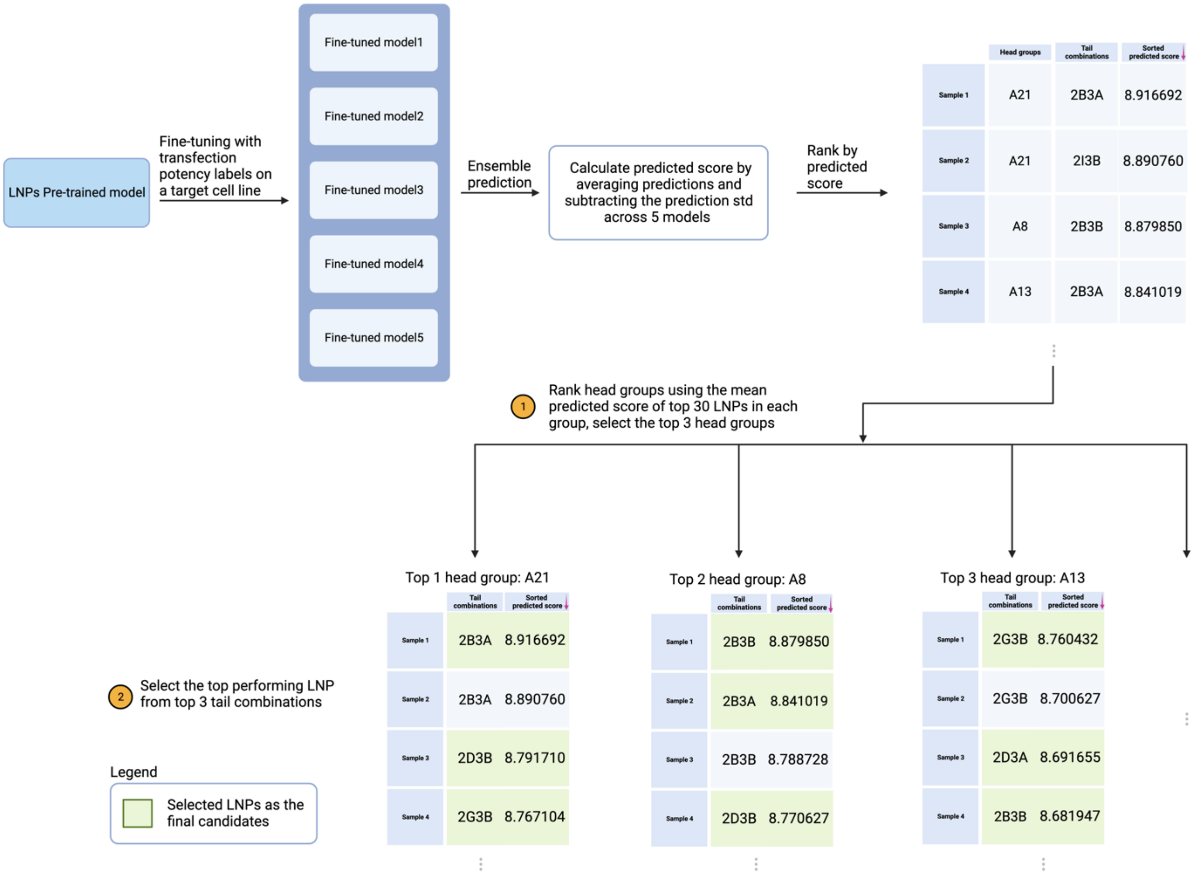

Supplementary Figure 37. This figure provides a comprehensive outline of the ranking and selection procedure implemented by AGILE, exemplified by the selection of the top three head groups and top three tail combinations as the final candidates. Initially, AGILE, pre-trained with lipids, is fine-tuned using measured transfection potency labels for a specific target cell line. The five optimal fine-tuned models are preserved for ensemble prediction. During this ensemble prediction stage, we initially calculate the predicted score by averaging the predictions and then subtracting the standard deviation of the predictions across the five models. This score is subsequently used to rank the samples. Upon obtaining this ranking, a head and tail-wise ranking is conducted to finalize candidate selection. The initial step utilizes the mean predicted score of the top 30 lipids in each group to rank the head groups, enabling the selection of the top three head groups. Subsequently, within these chosen head groups, the highest-performing lipid from the top three tail combinations is selected. This process results in a final selection of nine candidates, comprising three each from the top three head groups and tail combinations.

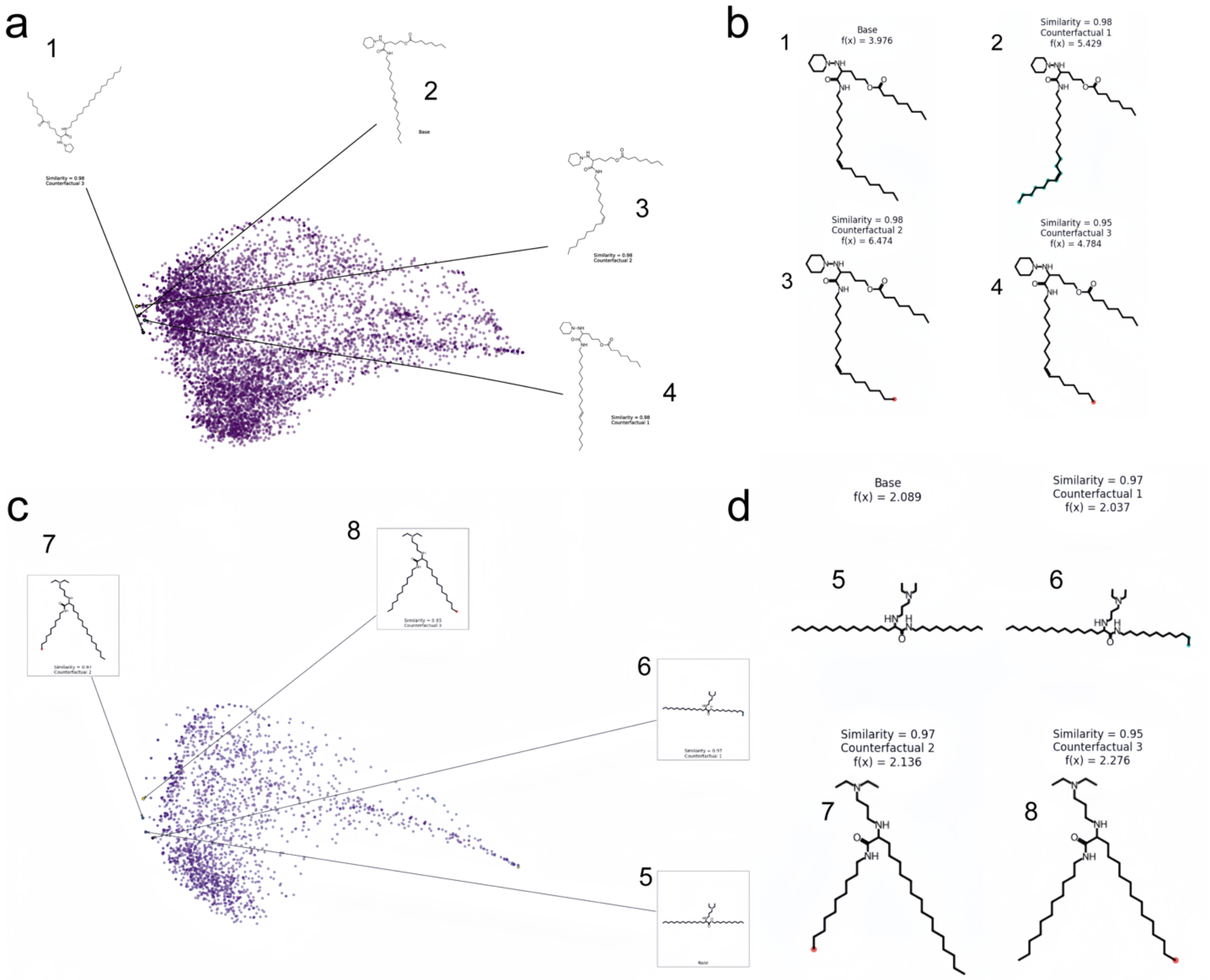

Supplementary Figure 38. Illustration of Exmol molecular explanation using counterfactual generation, where counterfactual molecules are generated by small modifications on the base molecule structure. This approach can explore the specific structure changes that may lead to the escalation or decline of the predicted transfection potencies. (a) The counterfactual space of H9. Each dot represents a counterfactual molecule. The base molecule, H9, is denoted as the left most one. (b) Four molecule structures representing the H9 and three counterfactual molecules, respectively. For each molecule, the output score by the AGILE graph encoder is provided above the structure as "f(x)". The similarities computed by Exmol between the counterfactual molecules and H9 are also listed. (c) The counterfactual space of R6. Each dot represents a counterfactual molecule. The base molecule, R6, is denoted as the left most one. (d) Four molecule structures representing the R6 and three counterfactual molecules, respectively. For each molecule, the output score by the AGILE graph encoder is provided above the structure as "f(x)". The similarities computed by Exmol between the counterfactual molecules and R6 are also listed.
