# Supplementary material for: AGILE platform: a deep learning powered approach to accelerate LNP development for mRNA delivery
Source: Nat Commun. 2024 Jul 26;15:6305. doi: 10.1038/s41467-024-50619-z (PMC11282250; doi:10.1038/s41467-024-50619-z)
Supplement: Supplementary file 2 — Peer Review File [file 41467_2024_50619_MOESM2_ESM.pdf]

nature portfolio

Peer Review File

AGILE Platform: A Deep Learning Powered Approach to Accelerate LNP Development for mRNA Delivery

Open Access This file is licensed under a Creative Commons Attribution 4.0 International License, which permits use, sharing, adaptation, distribution and reproduction in any medium or format, as long as you give appropriate credit to the original author(s) and the source, provide a link to the Creative Commons license, and indicate if changes were made. In the cases where the authors are anonymous, such as is the case for the reports of anonymous peer reviewers, author attribution should be to 'Anonymous Referee' followed by a clear attribution to the source work. The images or other third party material in this file are included in the article's Creative Commons license, unless indicated otherwise in a credit line to the material. If material is not included in the article's Creative Commons license and your intended use is not permitted by statutory regulation or exceeds the permitted use, you will need to obtain permission directly from the copyright holder. To view a copy of this license, visit http://creativecommons.org/licenses/by/4.0/.

Editorial Note: This manuscript has been previously reviewed at another journal that is not operating a transparent peer review scheme. This document only contains reviewer comments and rebuttal letters for versions considered at *Nature Communications*.

REVIEWER COMMENTS

Reviewer #1 (Remarks to the Author):

Thanks for the authors responses. Here, I still have concerns about the manuscript.

Based on the PNAS publication (2023, 120 (50) e2309472120), the authors improved the reaction efficiency. If the estimated yield of all lipids falls within the range of 70% to 90%, it raises questions about the unidentified 10% to 30% and their potential impacts on in vitro mRNA delivery. Can we rely on precise AI predictions for refining lipid designs using this screening library? While it's acknowledged that purifying all lipids from such a vast combinatorial library is challenging, uncertainties surrounding screening accuracy prompt us to reconsider the value of high-throughput screening. Without confidence in the screening process, how can we justify its utility?

Reviewer #2 (Remarks to the Author):

After reviewing the revised manuscript, I am pleased to note that the authors diligently addressed the comments raised. The overall improvement of the manuscript is significant and hold potential for advancing the filed of lipidnanoparicle technology. I don't have any further comments and in my opinion the manuscript is suitable for publication in nature communication.

Reviewer #3 (Remarks to the Author):

Comment 1:
Thank you for providing the characterization data for H1-H15 lipids.

There are examples that support my initial point: Angewandte Chemie 62 (43), e202310401, where the He et al synthesized and purified 161 novel ionizable lipids via Ugi-4CR reaction and then from there made 75 novel lipid nanoparticles for testing; and Biomaterials 301, 122243, where Goldman et al synthesized and purified >250 novel ionizable lipids for making 250+ novel lipid nanoparticles.
To clarify my initial comment again, from a practical point, I agree that it is time-consuming to purify all 1,200 of the authors' synthesized ionizable lipids. From a scientific point, I disagree this should be accepted as "consistent with standard practices in the field" or that "characterization is not a prerequisite for high-throughput screening contexts". Another reviewer has also pointed out these problems as well. The value of structure-activity relationships with impure crude products is questionable, as it is unclear what molecules are actually present and used to form LNPs. My suggestion to the authors is to explicitly state that crude lipid products is a limitation of this dataset and study. Especially as the authors have stated in lines 390-391, "given that the model's accuracy is inherently tied to the quality and breadth of data available". It is likely that data quality could be improved if the purity of the novel ionizable lipids used to generate the training data were improved.

Comment 2:
Thank you for providing the characterization data for H31-H45 LNPs per comments by the other reviewers.

The references to Nat Biomed Eng 5, 1059-1068 (2021), ACS Nano 17, 12, 11454-11465 (2023), Int J Pharm 599, 120392 (2021) support the fact that these papers appropriately provided basic characterization data (size and PDI) of their entire LNP library; not in reference to universal purification of high-throughput LNP libraries. Please refer to Nat BioMed Eng 5, 1059-1068 (2021) supplementary figures 1-3 (56 LNPs); ACS Nano 17, 12, 11454-11465 (2023) supporting tables S1-S2 (54 LNPs); and Int J Pharm 599, 120392 (2021) Figure 3 (32 unique LNPs in triplicate). Providing basic characterization data of all nanoparticles produced in studies for a manuscript is in line with the standard in the field, and important for improving reproducibility, quantification, and comparability as described in detail in Nature Nano 13, 777-785 (2018), Nature Nano 14, 629-635 (2019), and Nature Nano 15, 2-3 (2020). Presumably, the authors should have the size, PDI, and encapsulation efficiency data for all 1,200 LNPs as they would need to confirm that they indeed successfully synthesized all the different LNPs and encapsulated the mRNA cargo prior to downstream in vitro testing. The encapsulation efficiency is critical to normalize the amount of RNA dosed for each LNP formulation in the high-throughput in vitro screen with cells. Biases in doses and particle quality will render any models from this publication irreproducible. All these basic characterization data need to be included in the supplementary information.

With regards to concerns about LNP purification, since the authors were unable to find the relevant sections of the literature referenced, I have included and bolded/underlined the relevant sections for the authors' convenience.

• Nat Biomed Eng 5, 1059-1068 (2021), Methods section >> Nanoparticle characterization: "To avoid using unstable LNPs and enable sterile purification using a 0.22 μm filter, LNPs were included only if they met three criteria: diameter >20 nm, diameter <200 nm and correlation function with 1 inflection point. For screens, particles that met these criteria were pooled into respective groups. Particles were dialysed in 20 kD dialysis cassettes (Thermo Fisher Scientific). "
• ACS Nano 17, 12, 11454-11465 (2023), Materials and Methods >> High-Throughput Formulation and Characterization of LNPs: "In separate experiments, certain LNPs were processed through ultracentrifugation using an Amicon filter with the MWCO of 10 kDa (Millipore, MA, USA) for purification and buffer exchange to PBS."
• Int J Pharm 599, 120392 (2021), section 2.3 Characterization of ASO-loaded LNPs: "Sixty μl aliquots were adjusted to neutral pH (~7.0) by adding 15 μL of 0.5 M phosphate buffer (pH 7.4), then transferred to a filter plate (MWCO 100 kD; AcroPrep, PALL, NY, USA) and centrifuged (2,000 ×g, 10 min) for filtrates."
• Small 18, 9, e2105832 (2022), Experimental section >> LNP Preparations: Both standard and automated MC3 LNPs were made of the same lipids and compositions MC3:DSPC:cholesterol:DMG-PEG2k, 50:10:38.5:1.5 molar ratio) and were prepared as previously described.[29] In short, Nanoassemblr (Precision Nanosystems) and Hamilton liquid handling system (Microlab STAR) were used to prepare LNPs at three volumetric portions of mRNA to 1 volumetric portion of lipid solutions (mRNA: lipids 1:20 weight ratio) to obtain a lipid concentration of 2.81 mg mL−1 (4.80 mm). The clean-up of the crude formulations was achieved by dialysis in PBS using Slide-A-Lyzer G2 dialysis cassettes (Thermo Scientific, MWCO 10 K) or 96 well microdialysis membrane (Thermo Scientific Pierce, MWCO 10 K).

The examples I provided are relevant to the authors' submission because these papers either used an automated liquid handler to make LNPs (as the authors do in this submission) or prepared a large library of LNPs (i.e.: 56, 54, 32 x triplicate). My inclusion of these examples is relevant to discussion and comparison with the authors' work here. I disagree, as the authors rebut, that these reference literatures do not directly support my critique or misinterpret the scope and findings of the referenced works. With direct reference to the Small (2022) paper, Cui et al used a 96-well microdialysis membrane for LNP dialysis, which is applicable to the automated liquid handler approach as discussed, regardless if one is using an automated liquid handler to make a few LNP designs, or an entire 100+ LNP design library. LNP dialysis and filtration is important as it has huge effects on LNP physicochemical properties as the solvent conditions affect LNP assembly, stability, and ionization behavior.

In addition to providing the characteriziation data, the authors need to mention how using crude LNPs synthesized from an automated liquid handler without filtration or dialysis is a limitation of this dataset and study. Presumably, the model's accuracy could be further improved if the quality and purities of the novel LNPs used to generate the training data were improved as well.

Comment 3a:
Thank you for including the error reporting and for providing a more detailed methodology.

Comment 3b:
Comparing the new and previous Supplementary figures, I don't believe the authors made any changes to S16 and S28. The typos in Figure S16 and S28 are still present – "Kidney" is spelt as "Kindey" in the labelled images. I believe the IVIS imaging quantification units are still incorrect. The scale legend units should be "radiance", not "total radiance efficiency"; and the quantification graph should have the units [p/s]/[μW/cm2] since this is presumably derived from the ROI. Please refer to this technical note from PerkinElmer for details:
https://resources.perkinelmer.com/corporate/cmsresources/images/44-171013tch_012007_01_ivis-2d_3d_imaging.pdf

Comment 4:
Thank you for including the baseline AST/ALT values and for refining the AIH discussion.

Comment 5:
I appreciate the insight provided by the authors here about the dataset size required for AGILE training.

Reviewer #4 (Remarks to the Author):

The manuscript „AGILE Platform: A Deep Learning-Powered Approach to Accelerate LNP Development for mRNA Delivery" describes a deep learning approach to discover novel LNP. The authors describe a state-of-the-art model building pipeline starting with pre-training a GNN model followed by a fine-tuning step. Finetuning is done on sub-library of 1200 molecules. The authors present some validation data on the method based on 80-10-10 split of the data derived from the sub-library.
The general strategy is a promising strategy which combines an experimental with an in-siiico approach to accelerate learning and to identify relevant molecular features. In that respect, the use of various concepts to explain model prediction (feature importance and counterfactuals) is a highly relevant concept.
Nevertheless, model validation and results require more discussion on the contribution of the deep learning model to compound selection.
-The validation study only shows a model of mediocre predictivity (correlation coefficients, precision matrix). Explain, why you have selected 6 different classes for the precision matrix? How does a different partition influence the performance metric?
-Results for the 30 molecules have a significant number of lower ranked molecules with decent performance according to thresholds of fig. S5. Please elaborate in more detail, what the predictivity of the model contributes to the selection in comparison to the diversity-based approach.
I am not sure to understand Figure S36 in that context.
Also explain what the statistical basis of the threshold is depicted in Figure S5 – half of the of the screening set of 1200 molecules is marked as acceptable or outstanding.
-According to Figure S38, both H9 and R6 appear to be at the edge of the training space. Comment on the extrapolative aspect of your model.
-How is the scaffold split done? How different is the validation data set from the training data?
Overall, the manuscript presents an interesting approach with an integrated in-silico and experimental approach to identify novel LNPs. Nevertheless, before publication the contribution of the predictive model to the selection should be discussed more thoroughly.

Minor things.
-plots missing or wrong annotation in Figure S6 and S23
Typos: "Person" table S5

Point-by-point response to reviewer comments

We thank the editor and reviewers for their insightful and constructive feedback, which has significantly enhanced the quality and depth of our manuscript. Below is a point-by-point response to each comment raised by the reviewers. All modifications made in the manuscript are marked in blue.

Reviewer #1 (Remarks to the Author):
Thanks for the author's responses. Here, I still have concerns about the manuscript.

Comment 1:
Based on the PNAS publication (2023, 120 (50) e2309472120), the authors improved the reaction efficiency. If the estimated yield of all lipids falls within the range of 70% to 90%, it raises questions about the unidentified 10% to 30% and their potential impacts on in vitro mRNA delivery. Can we rely on precise AI predictions for refining lipid designs using this screening library? While it's acknowledged that purifying all lipids from such a vast combinatorial library is challenging, uncertainties surrounding screening accuracy prompt us to reconsider the value of high-throughput screening. Without confidence in the screening process, how can we justify its utility?

Response:
Thank you for your insightful questions. To meet the requirements for AI model training—particularly the necessary dataset size and diversity, we've adopted combinatorial chemistry and high-throughput screening strategy to rapidly generate diverse ionizable lipids, though this approach necessitated some compromises in lipid purification. While our primary goal in the present work is to demonstrate how AI can be integrated with combinatorial chemistry to accelerate the discovery of new ionizable lipids, we acknowledge that enhancing the purification process in a high-throughput setup could further improve data accuracy and model predictions. We would like to investigate this in our future studies. We appreciate your comment and have discussed the limitations of our current approach on page 10 of the revised manuscript: "Notably, the training data collected from crude ionizable lipids for AI model training represents a limitation of this study. Although it has been reported that Ugi-based multicomponent reactions have obtained acceptable conversion yields for HTS in vitro, purifying all ionizable lipids synthesized at the HTS stage is anticipated to improve the quality of the training data, which, in turn, enhance the precision of AI model predictions."

Point-by-point response to reviewer comments

Reviewer #2 (Remarks to the Author):
Comment 1:
After reviewing the revised manuscript, I am pleased to note that the authors diligently addressed the comments raised. The overall improvement of the manuscript is significant and hold potential for advancing the field of lipid nanoparticle technology. I don't have any further comments and in my opinion, the manuscript is suitable for publication in nature communication.
Response:
Thank you for your thorough review and positive feedback on the revised manuscript. We greatly appreciate your time and consideration.

Point-by-point response to reviewer comments

Reviewer #3 (Remarks to the Author):
Comment 1:
Thank you for providing the characterization data for H1-H15 lipids. There are examples that support my initial point: Angewandte Chemie 62 (43), e202310401, where the He et al synthesized and purified 161 novel ionizable lipids via Ugi-4CR reaction and then from there made 75 novel lipid nanoparticles for testing; and Biomaterials 301, 122243, where Goldman et al synthesized and purified >250 novel ionizable lipids for making 250+ novel lipid nanoparticles.
To clarify my initial comment again, from a practical point, I agree that it is time-consuming to purify all 1,200 of the authors' synthesized ionizable lipids. From a scientific point, I disagree this should be accepted as "consistent with standard practices in the field" or that "characterization is not a prerequisite for high-throughput screening contexts". Another reviewer has also pointed out these problems as well. The value of structure-activity relationships with impure crude products is questionable, as it is unclear what molecules are actually present and used to form LNPs. My suggestion to the authors is to explicitly state that crude lipid products is a limitation of this dataset and study. Especially as the authors have stated in lines 390-391, "given that the model's accuracy is inherently tied to the quality and breadth of data available". It is likely that data quality could be improved if the purity of the novel ionizable lipids used to generate the training data were improved.

Response:
Thank you for your insightful comments. We appreciate your input on the purification challenges of ionizable lipids and the implications for data quality in our study. While our primary goal in the present work is to demonstrate how AI can be integrated with combinatorial chemistry to accelerate the discovery of new ionizable lipids, we fully agree that enhancing the purification process in a high-throughput setup could further improve data accuracy and model predictions. We have revised the manuscript to clearly state these limitations and discuss their potential impact on the accuracy of AI model predictions: "Notably, the training data collected from crude ionizable lipids for AI model training represents a limitation of this study. Although it has been reported that Ugi-based multicomponent reactions have obtained acceptable conversion yields for HTS in vitro, purifying all ionizable lipids synthesized at the HTS stage is anticipated to improve the quality of the training data, which, in turn, enhance the precision of AI model predictions. [Angewandte Chemie 62 (43), e202310401]"

Comment 2:
Thank you for providing the characterization data for H31-H45 LNPs per comments by the other reviewers. The references to Nat Biomed Eng 5, 1059-1068 (2021), ACS Nano 17, 12, 11454-11465 (2023), Int J Pharm 599, 120392 (2021) support the fact that these papers appropriately provided basic characterization data (size and PDI) of their entire LNP library; not in reference to universal purification of high-throughput LNP libraries. Please refer to Nat BioMed Eng 5, 1059-1068 (2021) supplementary figures 1-3 (56 LNPs); ACS Nano 17, 12, 11454-11465 (2023) supporting tables S1-S2 (54 LNPs); and Int J Pharm 599, 120392 (2021) Figure 3 (32 unique LNPs in triplicate). Providing basic characterization data of all nanoparticles produced in studies for a manuscript is in line with the standard in the field, and important for improving reproducibility, quantification, and comparability as described in detail in Nature Nano 13, 777-785 (2018), Nature Nano 14, 629-635 (2019), and Nature Nano 15, 2-

3 (2020). Presumably, the authors should have the size, PDI, and encapsulation efficiency data for all 1,200 LNPs as they would need to confirm that they indeed successfully synthesized all the different LNPs and encapsulated the mRNA cargo prior to downstream in vitro testing. The encapsulation efficiency is critical to normalize the amount of RNA dosed for each LNP formulation in the high-throughput in vitro screen with cells. Biases in doses and particle quality will render any models from this publication irreproducible. All these basic characterization data need to be included in the supplementary information. With regards to concerns about LNP purification, since the authors were unable to find the relevant sections of the literature referenced, I have included and bolded/underlined the relevant sections for the authors' convenience.

- *Nat Biomed Eng 5, 1059-1068 (2021), Methods section >> Nanoparticle characterization: "To avoid using unstable LNPs and enable sterile purification using a 0.22 μm filter, LNPs were included only if they met three criteria: diameter >20 nm, diameter <200 nm and correlation function with 1 inflection point. For screens, particles that met these criteria were pooled into respective groups. Particles were dialyzed in 20 kD dialysis cassettes (Thermo Fisher Scientific).*
- *ACS Nano 17, 12, 11454-11465 (2023), Materials and Methods >> High-Throughput Formulation and Characterization of LNPs: "In separate experiments, certain LNPs were processed through ultracentrifugation using an Amicon filter with the MWCO of 10 kDa (Millipore, MA, USA) for purification and buffer exchange to PBS."*
- *Int J Pharm 599, 120392 (2021), section 2.3 Characterization of ASO-loaded LNPs: "Sixty μl aliquots were adjusted to neutral pH (~7.0) by adding 15 μL of 0.5 M phosphate buffer (pH 7.4), then transferred to a filter plate (MWCO 100 kD; AcroPrep, PALL, NY, USA) and centrifuged (2,000 ×g, 10 min) for filtrates."*
- *Small 18, 9, e2105832 (2022), Experimental section >> LNP Preparations: Both standard and automated MC3 LNPs were made of the same lipids and compositions MC3:DSPC:cholesterol:DMG-PEG2k, 50:10:38.5:1.5 molar ratio) and were prepared as previously described.[29] In short, Nanoassemblr (Precision Nanosystems) and Hamilton liquid handling system (Microlab STAR) were used to prepare LNPs at three volumetric portions of mRNA to 1 volumetric portion of lipid solutions (mRNA: lipids 1:20 weight ratio) to obtain a lipid concentration of 2.81 mg mL−1 (4.80 mm). The clean-up of the crude formulations was achieved by dialysis in PBS using Slide-A-Lyzer G2 dialysis cassettes (Thermo Scientific, MWCO 10 K) or 96 well microdialysis membrane (Thermo Scientific Pierce, MWCO 10 K).*

The examples I provided are relevant to the authors' submission because these papers either used an automated liquid handler to make LNPs (as the authors do in this submission) or prepared a large library of LNPs (i.e.: 56, 54, 32 x triplicate). My inclusion of these examples is relevant to discussion and comparison with the authors' work here. I disagree, as the authors rebut, that these reference literatures do not directly support my critique or misinterpret the scope and findings of the referenced works. With direct reference to the Small (2022) paper, Cui et al used a 96-well microdialysis membrane for LNP dialysis, which is applicable to the automated liquid handler approach as discussed, regardless if one is using an automated liquid handler to make a few LNP designs, or an entire 100+ LNP design library. LNP dialysis and filtration is important as it has huge effects on LNP physicochemical properties as the solvent conditions affect LNP assembly, stability, and ionization behavior. In addition to providing the characterization data, the authors need to mention how using crude LNPs synthesized from an automated liquid handler without filtration or dialysis is a limitation of this dataset and study. Presumably, the model's accuracy could be further improved if the quality and purities of the

novel LNPs used to generate the training data were improved as well.

Response:

We thank the reviewer for the detailed and insightful comments. Our training model inputs ionizable lipid structural information and outputs mRNA transfection potency predictions. Notably, particle size, PDI, and encapsulation efficiency are not explicit inputs but influence transfection potency as latent variables during model training. These physicochemical factors are learned indirectly by the AI through gradient backpropagation, as variations in these parameters can affect the observed transfection results. For example, LNPs with suboptimal physicochemical properties tend to show reduced mRNA transfection potency, which our AI model learns to associate with less suitable lipid structures, even without explicit physicochemical data input. Therefore, the outcomes in our dataset inherently reflect these physicochemical variations, allowing indirect learning about their impacts on transfection potency.

Meanwhile, we acknowledge that incorporating direct physicochemical data (size, PDI, etc.) of purified LNPs into the model might further improve its precision and and would like to investigate this in our future studies. Regarding this, we have revised our manuscript (page 10) to discuss potential enhancements: "Additionally, including a filtration or dialysis step after the synthesis of LNPs using the automated liquid handler and incorporating the physicochemical characterization data of all purified LNPs in a high throughput manner might also enhance the wet-lab dataset quality, consequently boosting the accuracy of model predictions." Thank you again for your feedback.

Comment 3b:

Comparing the new and previous Supplementary figures, I don't believe the authors made any changes to S16 and S28. The typos in Figure S16 and S28 are still present – "Kidney" is spelt as "Kindey" in the labelled images. I believe the IVIS imaging quantification units are still incorrect. The scale legend units should be "radiance", not "total radiance efficiency"; and the quantification graph should have the units [p/s]/[µW/cm2] since this is presumably derived from the ROI. Please refer to this technical note from PerkinElmer for details: https://resources.perkinelmer.com/corporate/cmsresources/images/44171013tch_012007_01_ivis-2d_3d_imaging.pdf

Response:

Thank you for pinpointing our mistakes. We have revised the Figure S16 and S28 in the manuscript based on your suggestions.

Point-by-point response to reviewer comments

Reviewer #4 (Remarks to the Author):
The manuscript „AGILE Platform: A Deep Learning-Powered Approach to Accelerate LNP Development for mRNA Delivery" describes a deep learning approach to discover novel LNP. The authors describe a state-of-the-art model building pipeline starting with pre-training a GNN model followed by a fine-tuning step. Finetuning is done on sub-library of 1200 molecules. The authors present some validation data on the method based on 80-10-10 split of the data derived from the sub-library. The general strategy is a promising strategy which combines an experimental with an in-siiico approach to accelerate learning and to identify relevant molecular features. In that respect, the use of various concepts to explain model prediction (feature importance and counterfactuals) is a highly relevant concept. Nevertheless, model validation and results require more discussion on the contribution of the deep learning model to compound selection.

Response:
We sincerely thank the reviewer for the thoughtful and constructive feedback on our manuscript. Your insightful comments regarding the validation and model explanation have provided us with valuable perspectives to refine our discussion and strengthen the manuscript.

Comment 1:
The validation study only shows a model of mediocre predictivity (correlation coefficients, precision matrix). Explain, why you have selected 6 different classes for the precision matrix? How does a different partition influence the performance metric?

Response:
We thank the reviewers for their insightful comments and the opportunity to elaborate on the performance visualization and metrics:
Choice and Rationalization of Classes for the Precision Matrix:
Our decision to employ a precision matrix was driven by the need to transparently and comprehensibly evaluate the model's performance. The precision matrix is particularly adept at demonstrating how well the model discriminates between classes, which is critical for our application. The selection of six classes was based on considerations to enhance our results' interpretative clarity. Given the inherent variability in experimental conditions, we observed that minor ranking differences among lipids (e.g., a lipid ranked at 300 versus another at 310 out of 1200) do not always indicate their relative transfection potency superiority to one another, but rather indicate that they are performing similarly. Taking the experiment shown in Figure 4e as an example, the average standard deviation of the mTP among the replicates, ⅓ (std_H9 + std_MC3 + std_R6), is 0.49 on RAW 264.7 and 0.34 on Hela cells. Both numbers are much larger than the mTP difference among similarly ranked lipids in the 1200 experimental data. This observation led us to categorize the lipids into subgroups, thereby reducing the noise from minimal ranking variances in visualization and assessment. We found that using six classes highlights significant distinctions while filtering out irrelevant data. It can be shown in this six-scale evaluation that AGILE demonstrates an advantage by correctly identifying a top 16% performer 41% of the time, compared to the otherwise much lower probability of random selection (16%). This underscores AGILE's improvement in lipid selection. From another perspective, a 6-scale rating also aligns with commonly used star rating systems (0-5 stars) when granularity and reliability are balanced.
Influence of Different Partitions on Performance Metrics:

Point-by-point response to reviewer comments

The choice of six classes was intended to balance granularity and interpretability. To test the robustness of this choice, we conducted additional assessments with five and seven classes, as shown in the appended figure. These results demonstrated consistent performance across different class configurations, indicating that our model effectively discriminates between high and low performers under various assessment schemes. This consistency supports the validity of our initial evaluation setup.

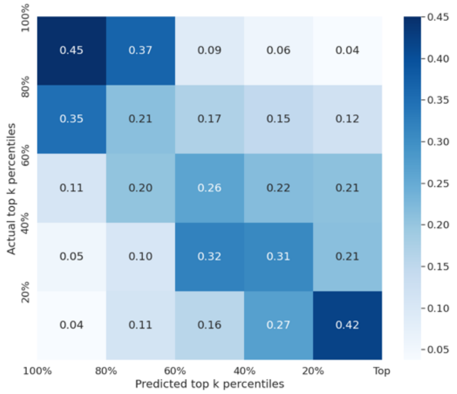

Five-class

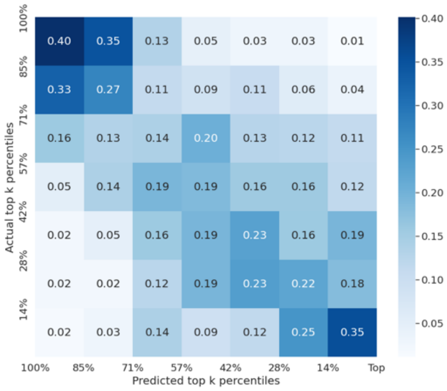

Seven-class

Comment 2:
Results for the 30 molecules have a significant number of lower ranked molecules with decent performance according to thresholds of fig. S5. Please elaborate in more detail, what the predictivity of the model contributes to the selection in comparison to the diversity-based approach. I am not sure to understand Figure S36 in that context. Also, explain what the statistical basis of the threshold is depicted in Figure S5 – half of the of the screening set of 1200 molecules is marked as acceptable or outstanding.

Response:
Thank you for your insightful comments. To clarify, the total library that the model ranks contains around 20,000 lipid structures. Therefore, the molecules ranked from 31 to 45, although lower in rank than the top-ranked molecules from 1 to 15, still maintain a relatively high position within the overall rankings. This explains their decent performance. To clarify, Figure S36 was referenced in Methods 1.6 on page 16 to justify our head and tail-wise reranking scheme. As discussed in Methods 1.6, the raw predictions of the model would give similar mTP values for lipids with the same head group and similar tails. Although this phenomenon is computationally rational, it reduces diversity among top-ranked candidates. This is visualized in Figure S36 by the high similarity within the top lipids when ranked by raw scores. For this reason, we introduced the reranking in the following context in Methods 1.6 and Figure S37.

Comment 3:
According to Figure S38, both H9 and R6 appear to be at the edge of the training space. Comment on the extrapolative aspect of your model.

Response:

Point-by-point response to reviewer comments

Thank you for your query regarding Figure S38 and the extrapolative power of our model. To clarify, Figure S38 is not a depiction of the training space but an interpretation plot generated using the ExMol Python package. As described in the figure caption, "counterfactual molecules are generated by small modifications on the base molecule structure, and each dot represents a counterfactual molecule." This figure intends to visualize molecular counterfactuals that clarify how specific structural modifications affect predictions. The ExMol usage is also detailed in the online documentation (https://ur-whitelab.github.io/exmol/#counterfactual-generation), which helps identify critical regions in the lipid structure by generating counterfactuals that maintain similarity with the input molecule while altering the predicted mTP. We believe the phenomenon of base molecules locating at the edge of the low-dimension mapping is mostly because of the counterfactual generation process: counterfactual examples are generated by gradually altering the atoms in base molecules, for example, first adding one carbon along the carbon chain and then adding another carbon atom. So this will naturally make the embeddings of these altered molecules gradually differ from the base molecule along some "axis" in the low-dimensional visualization, and push the base toward one end of the axis. In summary, we believe this is a visualization effect that occurred during the counterfactual explanation process. Similar effects would also be generally observed regardless of which method is being explained, exemplified by this use case in the online documentation of Exmol https://ur-whitelab.github.io/exmol/#usage.

Regarding the extrapolative aspect, training data is foundational for the accuracy and generalizability of the AGILE model. We have discussed the generalizability of both the pretraining and fine-tuning data in the discussion section on page 11. To summarize, (1) We acknowledge that the current AGILE model, fine-tuned with data from 1200 lipids synthesized via the 3-CR method, may have limitations in accurately predicting the performance of lipid structures beyond those in its training set. This highlights the importance of ensuring that the training data encompass a broad representation of possible molecular configurations to maintain accuracy across diverse test scenarios. (2) The pretraining phase is also introduced specifically in consideration to relieve the above constraint to an extent: by the self-supervised learning over vast amounts of lipid-like structures not limited to the experimented ones, the model is expected to learn more generalizable features. We indeed observed the higher accuracy of the pre-trained, fine-tuned model in comparison to the model without pretraining in our ablation experiments. For future direction, we expect the expansion of both pretraining and fine-tuning data to address this challenge more thoroughly. Hence, we have added a comment on the extrapolative aspect of the model on pages 11-12 "In addition, we acknowledge that the current AGILE model, fine-tuned with data from 1200 lipids synthesized via the 3-CR method, may have limitations in accurately predicting the performance of lipid structures beyond those in its training set. This highlights the importance of ensuring that the training data encompass a broad representation of possible molecular configurations to maintain accuracy across diverse test scenarios. (2) The pretraining phase is also introduced specifically in consideration to relieve the above constraint to an extent: by the self-supervised learning over vast amounts of lipid-like structures not limited to the experimented ones, the model is expected to learn more generalizable features. We indeed observed the higher accuracy of the pre-trained, fine-tuned model in comparison to the model without pretraining in our ablation experiments. For future direction, we expect the expansion of both pretraining and fine-tuning data to address this challenge more thoroughly."

Point-by-point response to reviewer comments

Comment 4:
How is the scaffold split done? How different is the validation data set from the training data? Overall, the manuscript presents an interesting approach with an integrated in-silico and experimental approach to identify novel LNPs. Nevertheless, before publication the contribution of the predictive model to the selection should be discussed more thoroughly.

Response:
Thank you for the comment. The dataset is split based on Murcko scaffolds (Bemis, Guy W., and Mark A. Murcko. 1996) to ensure a robust validation of our model. This is achieved by extracting the core scaffold of each molecule in the dataset using the Murcko Scaffold method in RDkit, which can optionally include chiral centers. Specifically, this strategy results in splitting lipids with the two headgroups, "A9" and "A14", exclusively in the validation set. Similarly, lipids with headgroups "A7" and "A11" are exclusively in the test set. This method ensures that molecules within any given training, validation set and testing set share a minimal structural similarity, thereby providing a stringent test of the model's ability to generalize to new molecular frameworks. The validation and testing set is specifically composed of different scaffolds from those in the training set, ensuring that the model's performance is assessed on completely novel structures. In response to the comment, we have introduced a discussion in the revised manuscript on page 13 "The dataset is split based on Murcko scaffolds (Bemis, Guy W., and Mark A. Murcko. 1996) to ensure a robust validation of our model. This is achieved by extracting the core scaffold of each molecule in the dataset using the Murcko Scaffold method in RDkit, which can optionally include chiral centers."

Comment 5:
Minor things.
plots missing or wrong annotation in Figure S6 and S23. Typos: "Person" table S5

Response:
Thank you for pointing out the typos and we have fixed them in the revised manuscript.

REVIEWERS' COMMENTS

Reviewer #1 (Remarks to the Author):

The authors have adequately addressed my concerns. I have no further questions.

Reviewer #3 (Remarks to the Author):

The authors have addressed all of my concerns. I have no further comments.

Reviewer #4 (Remarks to the Author):

The authors have addressed comments and clarified questions. I amm supportive of publishing the manuscript.
